# mTORC1 is a key mediator of RON-dependent breast cancer metastasis with therapeutic potential

**DOI:** 10.1038/s41523-018-0091-5

**Published:** 2018-11-09

**Authors:** Najme Faham, Ling Zhao, Alana L. Welm

**Affiliations:** Department of Oncological Sciences, Huntsman Cancer Institute, University of Utah, Salt Lake City, UT USA

## Abstract

Metastasis is the biggest challenge in treating breast cancer, and it kills >40,000 breast cancer patients annually in the US. Aberrant expression of the RON receptor tyrosine kinase in breast tumors correlates with poor prognosis and has been shown to promote metastasis. However, the molecular mechanisms that govern how RON promotes metastasis, and how to block it, are still largely unknown. We sought to determine critical effectors of RON using a combination of mutational and pharmacologic strategies. High-throughput proteomic analysis of breast cancer cells upon activation of RON showed robust phosphorylation of ribosomal protein S6. Further analysis revealed that RON strongly signals through mTORC1/p70S6K, which is mediated predominantly by the PI3K pathway. A targeted mutation approach to modulate RON signaling validated the importance of PI3K/mTORC1 pathway for spontaneous metastasis in vivo. Finally, inhibition of mTORC1 with an FDA-approved drug, everolimus, resulted in transient shrinkage of established RON-dependent metastases, and combined blockade of mTORC1 and RON delayed progression. These studies have identified a key downstream mediator of RON-dependent metastasis in breast cancer cells and revealed that inhibition of mTORC1, or combined inhibition of mTORC1 and RON, may be effective for treatment of metastatic breast cancers with elevated expression of RON.

## Metastasis: Druggable target mediates metastasis-promoting signal

A surface protein linked to metastasis in various cancers promotes the spread and migration of breast tumors by activating a druggable signaling pathway. Alana Welm and colleagues from the University of Utah, Salt Lake City, USA, engineered human breast cancer cells to conditionally overexpress RON, a receptor known to drive metastatic disease. They searched for proteins turned on by high RON levels, and found that one, called ribosomal protein S6 (rpS6), became highly decorated with phosphate groups, a sign of activation. Further analyses showed that an intermediary—the mechanistic target of rapamycin complex 1 (mTORC1)—relayed the activation signal to rpS6. The researchers thus inhibited mTORC1, both genetically and with an approved drug called everolimus, and observed transient shrinkage of metastatic breast tumors in mice, while combined blockade of mTORC1 and RON delayed metastatic progression.

## Introduction

Despite improvements in 5-year survival rates, breast cancer is still the second leading cause of cancer death among women. 90% of breast cancer deaths are due to the development of metastasis, which is still considered incurable even with the newest treatment options. Therefore, there is a clear need for a deeper understanding of the molecular mechanisms responsible for the development and progression of metastasis, and an urgent need for translation of that information to the development of effective therapies.

One promising therapeutic target that has emerged in recent years is the RON receptor tyrosine kinase. RON is a transmembrane tyrosine kinase that belongs to the MET proto-oncogene family.^1^ We previously reported that aberrant expression of RON kinase and its ligand, macrophage stimulating protein (MSP), correlates with poor prognosis in breast cancer patients, portending worse metastasis-free and overall survival.^2^ Multiple studies have also documented that RON overexpression strongly correlates with poor outcome in other cancers including lung, prostate, gastric, pancreas, and colon.^3–7^ Accordingly, expression of RON often increases in metastatic disease, which further points to an important role in late-stage cancer.^8^

The tumor progression phenotypes caused by RON activation, such as cell adhesion, spreading, survival, migration, and epithelial-to-mesenchymal transition (EMT), are the result of activation of complex downstream signaling networks including the PI3K, MAPK, JNK, β-catenin, and STAT pathways.^4,9^ However, different cancers appear to rely on different signaling pathways downstream of RON. For example, overexpression of RON in mouse mammary epithelium induced a tumorigenic phenotype and metastatic progression in lung and liver, which was associated with increased phosphorylation of MAPK and β-catenin.^10^ Further mechanistic studies in this model revealed a contributing, but not essential, role of β-catenin downstream of RON for mammary tumorigenesis.^11^ In leukemia and multiple myeloma, RON-induced IL-6 secretion seemed to underlie constitutive activation of the Jak/Stat3 pathway and poor prognosis.^9^ In gastroesophageal adenocarcinoma cell lines, RON was shown to signal through STAT3; inhibition of STAT3 was synergistic in decreasing viability in combination with a RON inhibitor.^6^ In an in vitro setting using noncancerous MDCK cells, activation of RON by MSP functioned in collaboration with TGF-β to enhance migration and cell motility through activation of MAPK/RSK2.^12–14^ In a separate study, despite simultaneous activation of MAPK, FAK, and c-Src pathways in RON overexpressing MDCK cells, MSP exerted its anti-anoikis effect via the PI3K pathway.^15^ Finally, in MCF-10A immortalized breast epithelial cells and in an MSP-independent setting, RON mediated cell migration, spreading, and survival through activation of c-Src signaling.^16^

Although they are less commonly expressed than full-length RON, alternative isoforms of RON have also been shown to mediate activation of different signaling pathways in several epithelial cancers.^17^ An example of a constitutively active variant of RON is short-form RON (sfRON). We have previously shown that overexpression of sfRON in nonmetastatic MCF7 breast cancer cells was sufficient to convert them into fast-growing, metastatic tumors. In vitro mechanistic studies revealed that sfRON promoted EMT and invasion through strong activation of PI3K, while MAPK signaling was decreased.^18^ Oncogenic signaling of sfRON in acute myeloid leukemia, however, functions through activation of the Src family kinase protein Lyn as well as Bcl-2, without affecting the PI3K pathway.^19^ In T47D breast cancer cells, loss of E-cadherin and increased motility induced by sfRON overexpression was mediated by the transcriptional factor SLUG.^20^ Additional isoforms of RON (e.g., splice variants) also induce activation of different signaling pathways, such as β-catenin in the case of RONΔ160, and AKT and MAPK in the case of RON-P5P6.^21,22^

Therefore, RON and its alternative isoforms signal in a cell context-dependent manner to mediate important tumorigenic phenotypes through various signaling pathways in vitro. However, an understanding of how RON mediates its metastatic function in the context of specific cancers in vivo is largely lacking, and is a critical gap in successfully developing strategies to block RON signaling during metastatic progression.

In human breast cancers, we showed that overexpression of either sfRON or RON alone, or RON and MSP together, was sufficient to drive spontaneous metastasis in cell line xenograft models.^18,23^ Furthermore, pharmacologic inhibition of RON significantly inhibited both orthotopic primary tumor growth and metastatic outgrowth of human breast cancer patient-derived xenografts overexpressing endogenous RON.^23,24^ However, despite clear evidence for elevated expression of RON in 100% of metastatic breast cancer samples examined,^8^ and its causal role in metastasis in breast cancer models, the molecular mechanisms that regulate the ability of RON to promote metastasis of human breast tumors have not yet been determined.

In the present study, we used a combination of mutational and pharmacologic approaches to interrogate the mechanism by which RON drives breast cancer metastasis. In addition, because some studies have pointed to different RON-mediated phenotypes in the presence and absence of its ligand, MSP,^15,16,25^ we sought to determine the requirement for MSP in RON-dependent human breast cancer metastasis. Using a detailed dissection of cellular signaling pathways in vitro and in vivo, we report that RON promotes both ligand-dependent and -independent breast cancer metastasis through activation of the mTORC1/p70S6K/rpS6 signaling axis.

## Results

### Tet-inducible expression of RON in T47D cells allows dissection of ligand-dependent and ligand-independent signaling

**Fig. 1:**
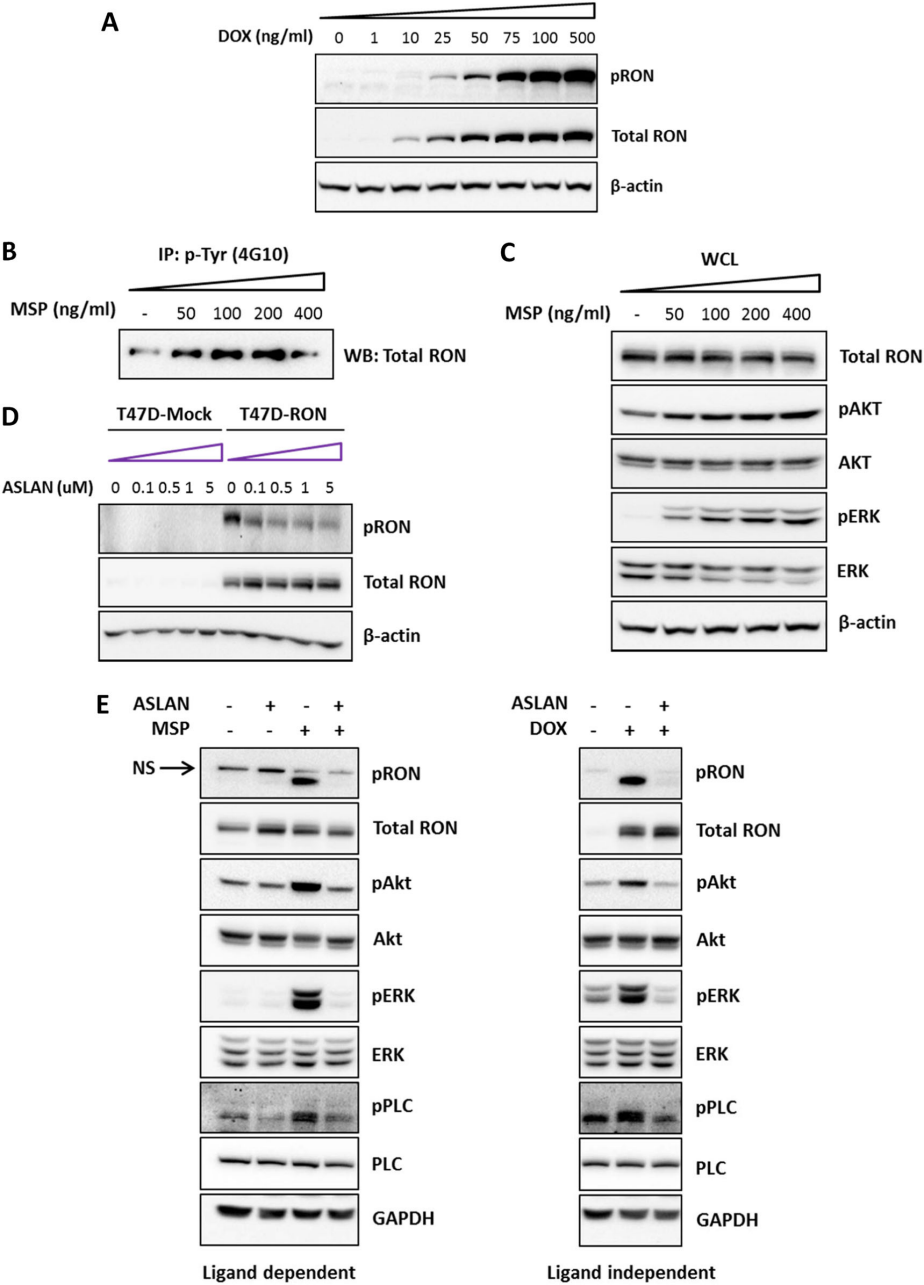
Tet-inducible expression of RON in T47D cells allows dissection of ligand-dependent and ligand-independent signaling. **a** Titration of RON expression by doxycycline in T47D cells. T47D-RON cells were treated with increasing concentrations of doxycycline for 48 h. Robust ligand-independent activation of RON is detected at higher concentrations of doxycycline, as reflected by increased pRON. **b** Dose-response stimulation of RON with MSP. T47D-RON cells were treated with doxycycline (100 ng/ml) for 48 h, and cultured in serum-starved media for 24 h. Cells were then stimulated with increasing concentrations of MSP for 15 min. Lysates were immune-precipitated with anti-4G10 phospho-tyrosine antibody, and subjected to Western blot analysis. **c** Whole cell lysates (WCL) of the same samples were analyzed for pAKT and pERK as readouts for activation of PI3K and MAPK pathways. β-actin was used as loading control. **d** Western blot shows the efficacy of a small molecule RON inhibitor, ASLAN002, at various concentrations on decreasing ligand-independent RON phosphorylation. Mock-infected T47D cells were used as a negative control. **e** Effect of ASLAN002 on two types of RON activation: MSP-dependent vs MSP-independent. T47D-RON cells were treated with low-level doxycycline (50 ng/ml) for 48 h. Cells were then serum starved for 24 h and treated with either RON inhibitor (ASLAN002, 1 μM) alone for 4 h, or RON inhibitor followed by MSP stimulation for 30 min. Cell lysates were tested for inhibition of RON and downstream signaling pathways (left panel). For ligand-independent RON activation, T47D-RON cells were treated with high dose of doxycycline (500 ng/ml) for 48 h in normal medium. Cells were then treated with RON inhibitor ASLAN002 (1 μM) for 4 h and analyzed for downstream signaling activity (right panel). GAPDH was used as loading control. Note that the left and right panels are from the same gel, separated for clarity. See also Supplementary Figure S1 and S2. NS non-specific band

RON activation in cancer can occur through MSP-mediated stimulation of the receptor and/or by overexpression of the receptor independent of its ligand.^4,17^ In order to examine both ligand-dependent and ligand-independent RON activation, we engineered human estrogen receptor positive breast cancer cells (T47D cells) to conditionally overexpress RON upon addition of doxycycline. This strategy enabled us to titer the expression level of RON by increasing the concentration of doxycycline. Higher concentration of doxycycline resulted in overexpression of RON, which caused robust ligand-independent activation, as reflected by increased pRON. Lower concentrations of doxycycline allowed RON expression, with enhanced activation by MSP (Fig. 1a, b). Dose-response analysis of MSP stimulation showed that 100 ng/ml of MSP induced robust phosphorylation of RON, as well as activation of downstream PI3K and MAPK signaling pathways (Fig. 1b, c). A time course analysis of MSP stimulation revealed that different signaling pathways showed different kinetics of activation upon RON phosphorylation. While activation of some signaling pathways occurred as very early events (PI3K and MAPK), phosphorylation of other downstream signal mediators occurred at slightly later time points (Src and PLC-γ; Supplementary Figure S1A-B). Similar results were obtained with a complementary approach, using the selective RON inhibitor ASLAN002. ASLAN002 (also known as BMS-777607) is an ATP-competitive small molecule kinase inhibitor of RON, MET, and AXL, with some activity also toward other TAM kinases.^26^ In T47D-mock-infected cells not expressing RON (T47D-Mock cells), we detected no off-target effect on the activity of downstream signaling pathways (Fig. 1d and Supplementary Figure S2), indicating that ASLAN002 could be used to dissect RON-specific activity in these cells. Under conditions in which doxycycline dosing was titrated to achieve equal levels of RON activation in MSP-dependent vs -independent conditions, we found that treatment of T47D-RON cells with 1 µM ASLAN002 inhibited RON phosphorylation and resulted in a profound reduction of downstream signaling activity, regardless of the type of RON activation (Fig. 1e). These studies revealed that RON activation in our experimental system reliably caused downstream activation of its known effectors, indicating that this system could be used to achieve our goal of discovering the critical downstream mediator(s) of RON signaling for breast cancer metastasis.

### Reverse-phase protein array identifies rpS6 as the most phosphorylated downstream target in response to RON activation in T47D breast cancer cells

**Fig. 2:**
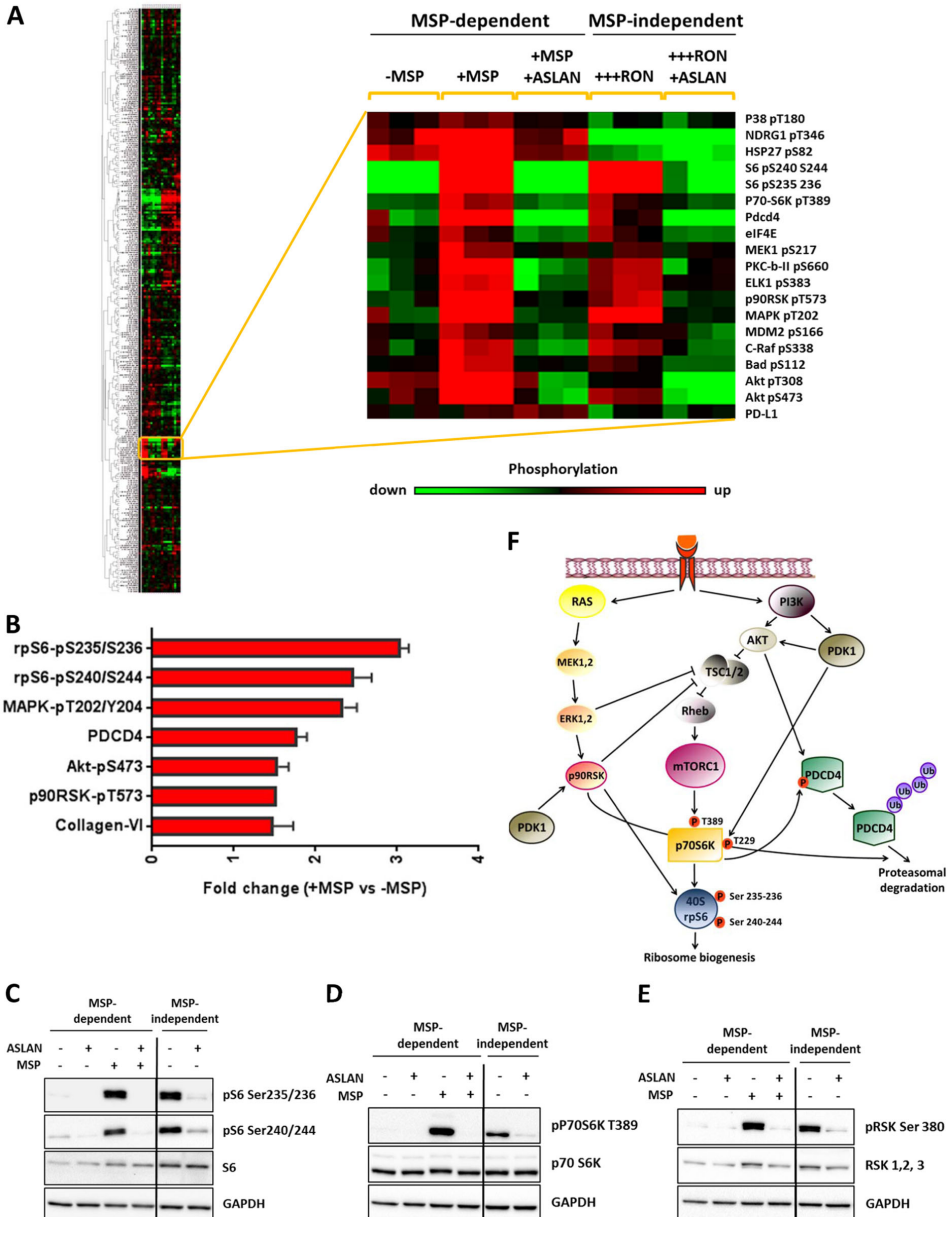
RPPA analysis of T47D-RON cells shows robust phosphorylation of rpS6 in response to RON activation. **a** Clustered heatmap of expression of 305 proteins is shown for T47D-RON cells in MSP-dependent and -independent conditions and with or without the RON inhibitor ASLAN002. For MSP-dependent analysis, T47D-RON cells were treated with a low level of doxycycline (50 ng/ml) for 48 h, serum-starved for 24 h, and stimulated with MSP (100 ng/ml) for 30 min. For MSP-independent analysis, T47D-RON cells were treated with a high level of doxycycline (500 ng/ml) for 48 h ± RON inhibitor (ASLAN002) for 4 h. Lysates from three biological replicates for each group were sent for RPPA analysis. A cluster of proteins that are particularly RON sensitive are enlarged on the right. A second group of proteins differentially present in the “MSP-dependent” vs “MSP-independent” conditions was not affected by the presence of MSP or RON inhibitor, was enriched in proliferation and cell-cycle regulation genes, and was likely due to differences in serum levels between the conditions. These proteins are shown in Supplementary Figure 18. **b** Graph shows candidates whose phosphorylation or expression changed >1.5 fold in response to MSP. **c**–**e** Validation of RPPA results by Western blot analysis. RON activation, whether through MSP stimulation or RON overexpression, causes strong phosphorylation of rpS6 (both serines), p70S6K, and RSK, which can be reversed by RON inhibition using ASLAN002. GAPDH was used as loading control. Line indicates the separation between lanes on the same Western blots. **f** Schematic diagram showing differentially phosphorylated proteins upon RON activation in the context of the cellular signaling network^31–34^

To gain further mechanistic insight into RON signaling beyond previously reported pathways, we measured the RON-dependent activity of hundreds of proteins within the cellular signaling network, using high-throughput reverse-phase protein array (RPPA) analysis. We again utilized T47D-RON cells with equal RON phosphorylation levels, in either MSP-dependent or -independent conditions (Fig. 1e), and with or without ASLAN002 treatment. A complete list of proteins assessed by RPPA is provided in the Supplemental Material. Hierarchical clustering of 305 proteins is shown in Fig. 2a (left panel), with the cluster of proteins whose phosphorylation specifically changed upon RON activation enlarged on the right. We quantified fold change for differentially phosphorylated proteins, and proteins whose expression changed upon MSP stimulation. Candidates that changed >1.5 fold are listed in Fig. 2b, which shows rpS6 was the highest differentially phosphorylated protein in response to MSP stimulation. Absolute values of significantly changed proteins or phosphoproteins with >1.5 fold change in response to MSP treatment are shown in Supplementary Table S1. We validated RPPA results by Western blotting for the proteins whose phosphorylation had significantly changed upon RON activation (Fig. 2c–e, and Supplementary Figure S3). Collectively, RPPA and immunoblotting data showed that RON activation, whether through MSP stimulation or RON overexpression, causes strong phosphorylation of rpS6, which can be reversed by the RON inhibitor ASLAN002.

### PI3K/mTORC1 is the predominant kinase responsible for phosphorylation of rpS6 downstream of RON

**Fig. 3:**
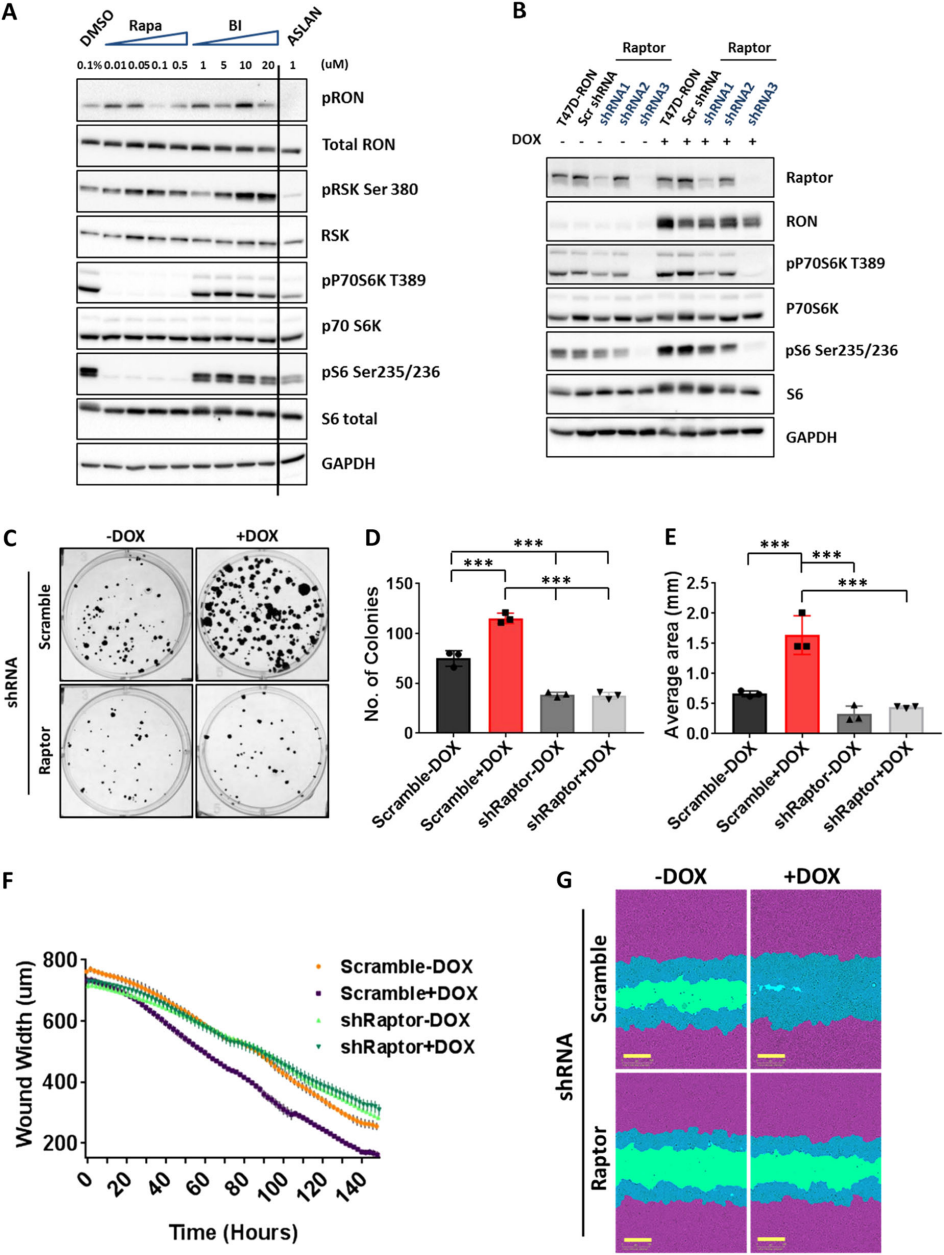
Signaling through mTORC1 is required for RON-mediated colony formation and migration in T47D-RON cells. **a** Representative Western blots for analysis of potential kinases upstream of rpS6 using specific inhibitors of pan-RSK (BI-D1870), mTORC1 (Rapamycin), and RON (ASLAN002). Treatment of T47D-RON cells in ligand-independent conditions with various doses of the inhibitors for 4 h shows almost complete inhibition of phospho-rpS6 by rapamycin, which also showed inhibition of p70S6K activity. See also Supplementary Figure S4A for signaling analysis using specific inhibitors of p70S6K. **b** Effect of Raptor knockdown on phosphorylation of rpS6 in T47D-RON cells. Cell lysates derived from T47D-RON cells stably expressing three different shRNA against Raptor or control shRNA (scramble), in the absence and presence of 500 ng/ml doxycycline, were subjected to immunoblotting. **c**–**e** Effect of mTORC1 knockdown on colony formation of T47D-RON cells. T47D-RON cells infected with scramble or Raptor shRNA construct # 3 (see panel **b**) were seeded at a very low density in the presence and absence of doxycycline and were allowed to form colonies, followed by crystal violet staining. Representative images are shown in panel **c**, whereas number and average area per colony are shown in panels **d** and **e**. Error bars indicate SD, *n* = 3. *P < 0.05, **P < 0.005, ***P < 0.0005 (one-way ANOVA, multiple comparisons). **f**, **g** Wound healing assays were performed to assess the effect of mTORC1 knockdown on the migration of T47D-RON cells. Doxycycline-treated (500 ng/ml) and untreated T47D-RON-sh*Raptor* and T47D-RON-sh-Scramble cells were seeded at high density the day before wounding. The rate of wound closure in each group over the course of treatment is shown in panel **f**. Representative images at day 6 are shown in panel **g**. The initial wound in each group is shown in blue/green; migrating cells from the initial wound are shown in blue. Scale bars represent 300 μm. Data are shown as mean ± SEM, *n* = 7

Phosphorylation of several proteins was found to be significantly upregulated upon RON activation in T47D-RON cells, including rpS6, p70S6K, PDCD4, p90RSK, AKT, and MAPK. Figure 2f shows a schematic diagram of these differentially phosphorylated proteins in the context of a cellular signaling network. As rpS6 was shown to be the highest differentially phosphorylated protein downstream of RON, we sought to determine the upstream potential kinases that could be responsible for its phosphorylation. p70S6K and p90RSK are the two bona fide rpS6 kinases,^27–29^ and both were shown to be differentially phosphorylated upon RON activation. To determine if either of these proteins, or both, were responsible for rpS6 activation, we performed a dose-response analysis with commercially available inhibitors of p70S6K and p90RSK, as well as mTORC1. These analyses, in the settings of either MSP-dependent or MSP-independent RON activation, showed almost complete inhibition of phospho-rpS6 by the mTORC1 inhibitor rapamycin, which also blocked p70S6K activity (Fig. 3a, and Supplementary Figures S4A and S5A). Despite some reduction in rpS6 phosphorylation, inhibition of p70S6K using two available inhibitors, LY2584702 and PF-4708671, did not give conclusive results due to confounding indirect effects on RON phosphorylation status (Supplementary Figure S4A). However, lower doses of PF-4708671 (an inhibitor of p70S6K1), in the setting of MSP-induced RON activation, reduced rpS6 phosphorylation with no effect on RON phosphorylation (Supplementary Figure S5A). These data suggested that activation of the mTORC1-p70S6K pathway was responsible for phosphorylation of rpS6, the major signaling output downstream of RON in T47D breast cancer cells.

To further investigate whether mTORC1 is the main kinase upstream of p70S6K and responsible for rpS6 phosphorylation downstream of RON, we took a genetic approach. We established three different stable Raptor knockdown T47D-RON lines, and found that depletion of Raptor strongly reduced the levels of phospho-rpS6 in the context of RON expression, with one Raptor shRNA showing complete knockdown and clear rpS6 inhibition (Fig. 3b).

Two of the best-studied regulators of mTORC1 are the PI3K/AKT and RAS/MAPK pathways,^30–34^ the effectors of which were shown to be among the differentially phosphorylated proteins downstream of RON in our RPPA data. To delineate the proximal signaling mediator(s) downstream of RON responsible for activation of the mTORC1/p70S6K/rpS6 axis, we tested inhibitors of PI3K and MAPK pathways in the context of RON activation. While successful MAPK inhibition in T47D-RON cells (shown by reduction in pERK and pRSK) only caused a slight decrease in phospho-rpS6, PI3K inhibition resulted in complete abrogation of p70S6K and rpS6 phosphorylation, in both MSP-dependent and MSP-independent RON activation settings (Supplementary Figure S4B and Supplementary Figure S5B).

To determine whether our findings of how RON signals in estrogen receptor positive (ER+) T47D cells would extend to other types of breast cancer cells, we engineered SUM-159PT cells (a cell line that is “triple negative” for ER, progesterone receptor, and the human epidermal growth factor receptor HER2) for conditional expression of RON using the same doxycycline-regulated system. RON overexpression in these cells was again accompanied by increased activation of mTORC1 pathway components, as reflected by enhanced phospho-p70S6K and phospho-rpS6 (Supplementary Figure S6A). Further analysis of these cells in MSP-independent conditions using inhibitors of mTORC1, PI3K, and MAPK pathways also indicated that mTORC1 signaling downstream of RON is fed strongly by PI3K, inhibition of which caused complete loss of phosphorylation of p70S6K and rpS6, similar to our results in T47D-RON cells (Supplementary Figure S6B). Signaling analysis in another triple negative breast cancer cell line, HCC-1143, with a high endogenous expression of RON, also confirmed strong upregulation of AKT, p70S6K, and rpS6 phosphorylation as a result of ligand-dependent RON activation, which could be reversed using the selective RON inhibitor ASLAN002 (Supplementary Figure S6C). These analyses showed that our observations on RON signaling is not restricted to ER+ breast cancer cells, and revealed that RON kinase signals strongly through PI3K/mTORC1/p70S6K/rpS6 in multiple types of breast cancer cells.

### mTORC1 suppression inhibits RON-mediated colony formation and migration in T47D-RON cells

RON kinase activity is known to promote metastatic features of cancer cells,^4^ but the mechanistic pathways linked to these phenotypes in breast cancer have not been well characterized. To elucidate the functional role of mTORC1 signaling downstream of RON, we performed colony formation assays in T47D-RON and T47D-RON-sh*Raptor* cells, in the presence and absence of doxycycline. T47D-RON-sh-Scramble was used as a negative control for off-target effects of shRNA expression. The results showed that overexpression of RON significantly increased colony formation. However, suppression of mTORC1, either through pharmacologic inhibition (rapamycin) or genetic depletion (shRaptor), significantly inhibited RON-mediated colony formation, as measured by colony number and total colony area (Fig. 3c–e, and Supplementary Figure S7A-C).

We next investigated whether mTORC1 regulates RON-mediated cell migration in T47D-RON cells. Wound healing scratch assays were performed in the presence and absence of doxycycline in T47D-RON and T47D-RON-sh*Raptor* (or sh-Scramble) cells, and migration of the cells was tracked over time to measure the rate of wound closure. Again, RON overexpression was sufficient to promote faster cell migration and wound closure. On the other hand, inhibition of mTORC1 signaling, either by treatment with rapamycin or through genetic knockdown, strongly inhibited RON-mediated migration of T47D-RON cells (Fig. 3f, g, and Supplementary Figure S7D-E). Collectively, these data indicate that RON is unable to promote its colony formation and cell migration functions in the absence of mTORC1 activity in T47D-RON cells.

### Targeted mutation of the RON kinase confirms RON-dependent PI3K/mTORC1/p70S6K/rpS6 signaling in T47D cells

**Fig. 4:**
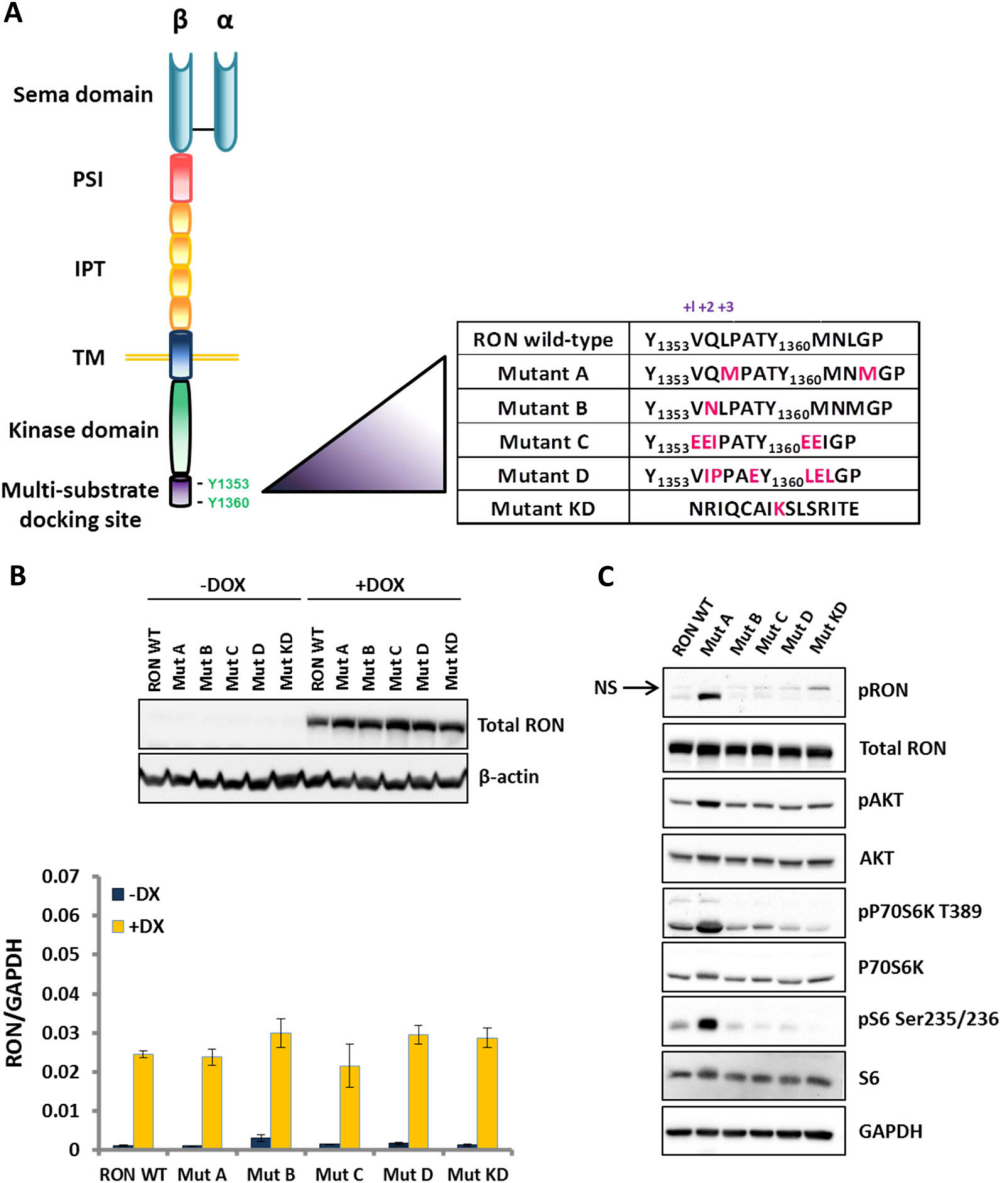
Inducible expression of different RON mutants in T47D cells and analysis of downstream PI3K/mTORC1 signaling. **a** Schematic presentation of RON kinase structure is shown in the left panel. Mutations that were introduced to RON are shown in the right panel. Mutated residues are highlighted in pink. **b** Inducible expression of RON mutants in T47D cells at the protein (upper panel) and mRNA level (lower panel). T47D cells transduced with different RON mutants in a Tet-inducible lentiviral plasmid were treated with 500 ng/ml doxycycline for 48 h, and analyzed by Western blot. Equal level of RON expression is apparent among the mutants, which is comparable to RON wild type (WT). Graph shows qRT-PCR analysis of T47D cells expressing RON mutants in the presence and absence of doxycycline (500 ng/ml) for 48 h with primers specific to RON. GAPDH was used as the internal control. **c** Signaling analysis of the PI3K/mTORC1 pathway in T47D cells expressing RON WT and mutants. T47D-RON WT and mutants were treated with 100 ng/ml doxycycline for 48 h, followed by serum starvation for 24 h. Western blot shows robust activation of AKT, P70S6K, and rpS6, as readouts for activation of PI3K and mTORC1 kinase pathways, in Mutant A compared to the other mutants. GAPDH was used as the internal control. See also Supplementary Figure S8 for signaling analysis of mutants, and Supplementary Figure S9 for the detailed signaling analysis of RON-Mut A in the context of MSP-dependent and -independent RON activation. Line indicates the separation between lanes on the same Western blots. PSI plexin–semaphorin–integrin, IPT immunoglobulin–plexin–transcription, TM transmembrane

**Fig. 5:**
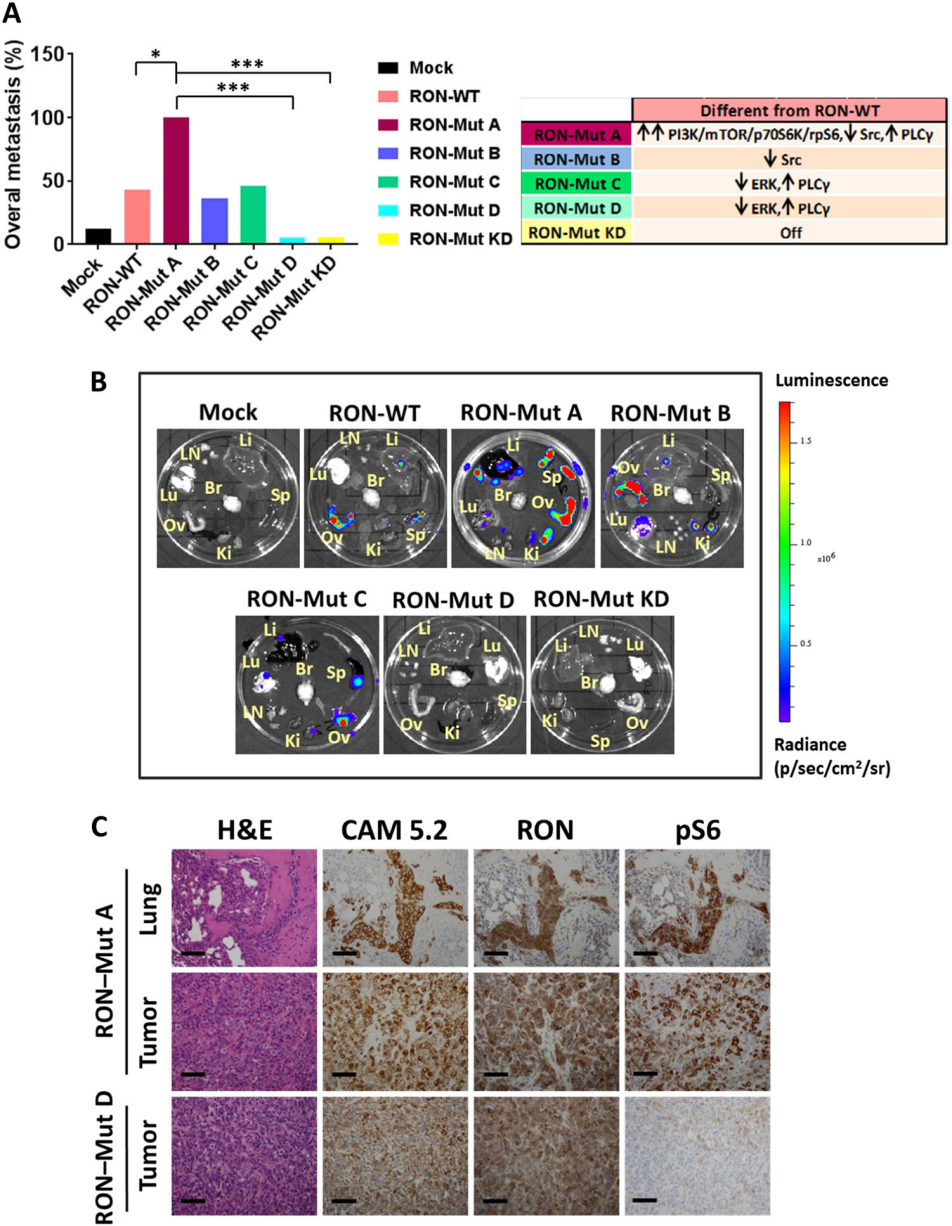
RON mutants show differential spontaneous metastatic potential in vivo. **a** Graph shows quantification of overall spontaneous metastasis in NOD/SCID mice following transplantation of T47D cells expressing different RON mutants. *P < 0.05, ***P < 0.0005 (Chi-square analysis, Fisher’s exact test). The table on the right summarizes differential activation of signaling pathways in different RON mutants compared to the RON-WT based on in vitro biochemical characterization. **b** Representative bioluminescence ex-vivo images of different organs from NOD/SCID mice bearing tumors from RON mutants. T47D-Luc-RON WT and mutants were orthotopically injected (5 × 10^6^ cells) into NOD/SCID mice. Tumors were harvested when they reached approximately 1300 mm^3^, and different organs were analyzed for metastasis by IVIS imaging. (See Supplementary Table S2 for animal numbers, tumor size and tissue tropism of metastasis in each group.) **c** Representative IHC staining for metastatic RON-Mut A and non-metastatic RON-Mut D. Staining for H&E, human-specific pan-cytokeratin CAM5.2, RON and pS6 (Ser 235/236) is shown for metastatic lung and primary tumor of RON-Mut A, and primary tumor of RON-Mut D. Scale bars represent 100 μm. See also Supplementary Figures S8 and S9. Li liver, Lu lung, Sp spleen, Ov ovary, Ki kidney, Br brain, LN lymph nodes

To investigate RON signaling with a complementary approach, we took advantage of a mutational screening strategy that has previously been used to dissect signaling of other receptor tyrosine kinases such as MET.^35^ This technique uses site-directed mutagenesis to create new receptor mutants as tools that alter the recruitment or activity of signaling adaptor proteins, thereby modulating downstream signaling and allowing assessment of the effect on particular phenotypes (in this case, metastasis). We introduced specific mutations in the first three residues flanking the two essential tyrosines in the C-terminal docking site of RON. As a negative control, we also generated a kinase-dead mutant (KD) by mutating a single amino acid in the kinase domain (Fig. 4a, right panel). Inducible expression of RON mutants in T47D cells in the presence of doxycycline showed equal levels of RON expression among the mutants, comparable to wild type RON (WT), both at the protein and mRNA level (Fig. 4b). We next screened each mutant in vitro for activation of downstream signaling pathways. Our data revealed that RON mutants signal differentially to downstream signaling pathways. While mutant A increased AKT activity more than the other mutants (Fig. 4c), mutants C and D showed stronger signaling through the PLC-γ and Src pathways (Supplementary Figure S8). A summary of the differential activation of signaling pathways in RON mutants compared to RON WT is provided in Fig. 5a, right panel. Further analysis of RON mutants for effectors of PI3K/mTORC1 signaling revealed strong activation of AKT, p70S6K, and rpS6 in mutant A compared to the other mutants (Fig. 4c). A dose-response inhibitor analysis in T47D-RON mutant A cells revealed that PI3K was again the dominant activator of mTORC1/p70S6K downstream of RON mutant A, while the MAPK pathway only partially contributed to rpS6 phosphorylation (Supplementary Figure S9A-B). We found no difference in p85 binding amongst RON mutants (Supplementary Figure S10), so mutant A augments PI3K signaling through an unknown mechanism that is not simply due to increased recruitment of p85. Activation of PI3K signaling by mutant A was consistent in both MSP-dependent and MSP-independent types of RON activation, similar to our observations in T47D cells expressing RON WT.

### RON mutants show differential spontaneous metastatic potential in vivo

We have previously shown that RON promotes spontaneous metastasis of breast tumors arising from orthotopic T47D, MCF7, and human patient-derived xenograft tumors,^18,23^ but it is still mostly unknown how RON mediates metastasis. The differential activation of signaling pathways by various RON mutants gave us the opportunity to analyze their metastatic potential in vivo. We injected T47D-Luc cells conditionally expressing different RON mutants into mammary fat pads of NOD/SCID mice. The mice were then given chow containing doxycycline to induce RON expression. Tumors were allowed to grow to approximately 1300 mm^3^, at which time tumors were harvested and organs were subjected to bioluminescence ex-vivo imaging. We found that, although tumors expressing different RON mutants grew at almost the same rate in the primary site (Supplementary Figure S11), they showed profoundly differential metastatic potential. While parental T47D tumors are known to be poorly metastatic in NOD/SCID mice (metastases were detected in only 12% of mice in our experiments), expression of WT RON increased metastatic frequency to 43% (Supplementary Table S2). RON mutant A increased metastasis further, to 100% of the mice, whereas mutants D and KD were incapable of promoting metastasis. Mutants B and C had a metastatic potential similar to that of WT RON (Fig. 5a, b, and Supplementary Table S2). These data indicate that RON mutants that are differentially able to activate downstream signaling pathways have different metastatic potential.

Based on our in vitro data, the most metastatic variant of RON, mutant A, increases signaling through the PI3K/mTORC1/p70S6K/rpS6 pathway. On the other hand, the least metastatic mutants, mutants D and KD, failed to activate this pathway. Thus, we next examined the status of cellular signaling in each cohort of tumors in vivo. IHC analysis of the primary tumors and metastatic lesions from RON WT and RON mutant A indicated strong phosphorylation of rpS6 compared to the non-metastatic tumors from RON mutant D or RON mutant KD (Fig. 5c and Supplementary Figure S12A). Indeed, Western blots on lysates from primary tumors expressing RON WT and mutant A showed robust phosphorylation of PI3K/mTORC1 pathway components AKT, p70S6K, and rpS6 when compared to tumors expressing RON mutant D or KD (Supplementary Figure S12B). Thus, our in vivo data combined with in vitro analysis of RON mutants revealed that signaling through the mTORC1/p70S6K/rpS6 axis is important for the metastatic function of RON.

Our in vitro data suggested that ligand-dependent and ligand-independent RON signaling is similar. Therefore, we also investigated whether ligand-dependent activation of RON would alter metastatic frequencies of RON mutants. Since mouse MSP does not activate human RON, we used mice that express human MSP, knocked into the endogenous mouse MSP locus (hMSP-NOD/SCID), for these experiments. We orthotopically implanted T47D-RON WT, RON mutant A, mutant D, and mutant KD into these mice, and measured tumor growth and metastasis. Our results showed that although RON mutant A grew in hMSP-NOD/SCID mice at a faster rate on average compared to the other groups, the difference was not statistically significant (Supplementary Figure S13). Bioluminescence ex-vivo imaging revealed that RON mutant A was still the most metastatic group, similar to our findings in the ligand-independent setting (100% of mice). Although there was a trend toward higher frequency of metastasis of RON WT tumors in hMSP-NOD/SCID mice compared to NOD-SCID mice (ligand-dependent vs ligand-independent settings), the difference was not statistically significant (*P* = 0.2, Chi-square analysis, Fisher’s exact test). Interestingly, mutant D and KD, which fail to activate PI3K and mTORC1 pathway components in vitro and in vivo, were still unable to promote metastasis, even in the presence of hMSP (Supplementary Figure S14A-B). These data suggest that the PI3K/mTORC1 pathway is critical for inducing metastasis downstream of RON, either in the absence or presence of hMSP.

### Pharmacologic abrogation of mTORC1 signaling diminishes outgrowth of established metastasis induced by T47D-RON cells

**Fig. 6:**
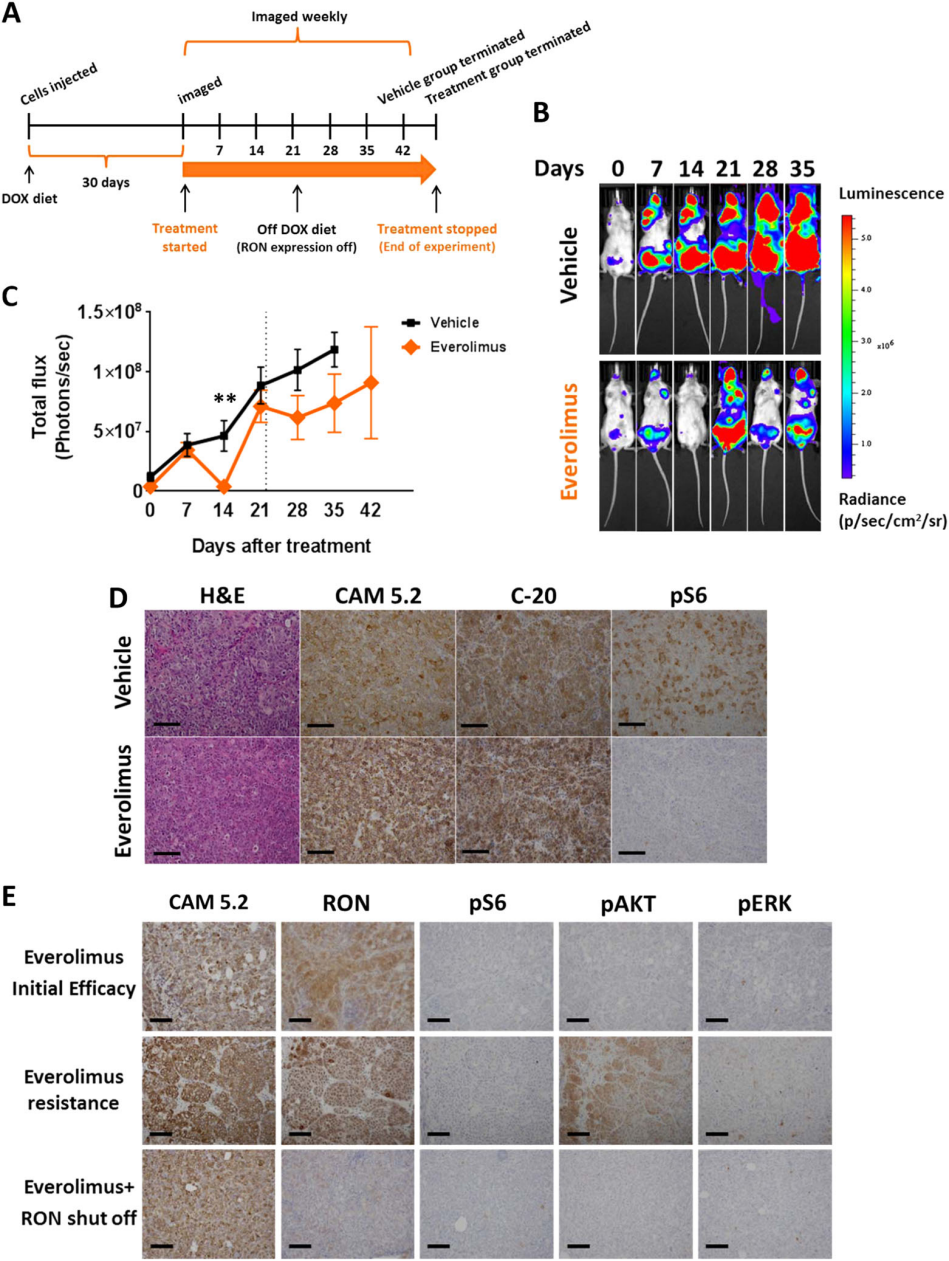
Effect of mTORC1 inhibition on established metastasis induced by T47D-RON cells. **a** Experimental scheme for established metastasis induced by T47D-RON cells and treatment with mTORC1 inhibitor, everolimus. T47D-RON cells were tail vein injected to NSG mice and mice were put on Doxy diet (50 ppm) to induce expression of RON. Treatment with everolimus or vehicle started at day 30 following cell injection, when metastatic lesions were detectable with in vivo IVIS imaging. Once mice developed recurrence of metastasis due to everolimus resistance, RON expression was blocked by removing doxy diet, and mice continued to be followed. **b** Representative IVIS images of mice over the course of treatment with everolimus or vehicle and with or without active RON expression, controlled by doxy diet. Mice were treated with everolimus (5 mg/kg) five times a week (5 days on, 2 days off), and were imaged weekly. **c** Graph showing quantification of metastasis in the everolimus (*n* = 11) vs vehicle-treated group (*n* = 12). Dashed line indicates the time when doxy diet was stopped to block RON expression. Data are shown as mean ± SEM, **P < 0.005 (unpaired *t*-test). **d** Representative images showing H&E, CAM5.2, RON, and pS6 (Ser 235/236) staining for metastatic lesions in the ovaries of mice treated with everolimus vs vehicle. **e** Status of pAKT and pERK as readouts for activation of PI3K and MAPK pathways, respectively, in metastatic lesions of ovaries through different stages of treatment with everolimus. Scale bars represent 100 μm. See also Supplementary Figure S15 and S16

Our in vitro and in vivo results collectively demonstrated that RON profoundly signals through mTORC1, downstream of PI3K. Our data from mutational studies in vivo also indicated that signaling through PI3K/mTORC1/p70S6k/rpS6 is critical to induce metastasis in the context of RON. We have previously tested the pan PI3K inhibitor BKM120, alone and in combination with RON inhibition. We found that it reduced tumor growth as a single agent, but the combination gave a more durable response than either inhibitor alone, albeit not curative.^24^ PI3K inhibitors are not currently approved for breast cancer, but an FDA-approved inhibitor for mTORC1 does exist. To test the effect of mTORC1 inhibition on RON-driven metastatic breast cancer, we established a model wherein T47D-RON cells were injected into the tail vein of NSG mice and then, upon clear detection of metastatic lesions by in vivo IVIS imaging, randomized to receive either an FDA-approved mTORC1 inhibitor, everolimus, or vehicle control treatment (see Fig. 6a for the experimental scheme, and Supplementary Figure S15 for tissue tropism of metastasis following injection of T47D-RON cells). This was intended to test the situation in which a new therapeutic regimen would be most likely to be tested in breast cancer patients (the established metastatic setting). After initiating therapy, mice were imaged weekly over the course of treatment. As shown in Fig. 6b–d, treatment with everolimus significantly shrunk metastases within the first 14 days of treatment, which was accompanied by effective blockade of its target, rpS6, in the metastatic lesions of mice treated with everolimus, compared to the vehicle group. However, resistance developed and the metastatic signal rebounded robustly by day 21, even though everolimus was still effectively inhibiting its target (Fig. 6e). We hypothesized that the mechanism of resistance could be due to the feedback loops within the RON kinase signaling network, and that inhibiting RON would reverse resistance. To test this hypothesis, we replaced the Doxy diet with normal diet to shut off RON expression in T47D-RON Tet-inducible tumors. Indeed, turning off RON decreased metastatic burden short term, but the metastases again rebounded (Fig. 6b, c) despite the loss of RON protein (Fig. 6e).

We next examined signaling effectors that might be responsible for resistance to mTORC1 inhibition in the context of RON signaling. Two of the most studied mechanisms of resistance to mTORC1 inhibitors are through feedback activation of PI3K/AKT, or overstimulation of the Ras/Raf cascade.^36–38^ We investigated pAKT and pERK levels as readouts for activation of PI3K and MAPK signaling, respectively, in the therapy-resistant metastatic lesions of mice treated with everolimus. IHC staining of the metastatic lesions from the resistance stage showed strong phosphorylation of AKT, as opposed to ERK, while phospho-rpS6 was still off (Fig. 6e; see also Supplementary Figure S16 for the status of phospho-S6, phospho-AKT, and phospho-ERK in metastatic lesions of mice in the vehicle group for comparison). Taken together, our data demonstrated that inhibition of mTORC1 could transiently shrink established RON-dependent metastases, but that the disease eventually progressed. Resistance to everolimus was accompanied by an increase in pAKT in the context of RON signaling network. When RON was subsequently shut off, pAKT was decreased and metastasis was again reduced. Unfortunately, the combined inhibition of mTORC1 and RON downregulation was not curative; metastases eventually continued to grow, albeit at a slower rate than control mice: combined inhibition of mTORC1 and RON did increase the overall survival time of mice compared to the control group.

### Analysis of human breast tumors reveals a high percentage of cases with RON expression and mTORC1 pathway activation

We examined archived primary and metastatic breast tumors for expression of RON and evidence of mTORC1 pathway activation using Western blot analysis. Metastases were obtained from lymph node or pleural effusions. Although we were unable to detect phosphorylated RON on frozen samples due to poor antibody sensitivity, we found that all tumor-containing samples (17/17; assessed by detection of the epithelial marker EpCAM) expressed RON, and all except one of those (16/17) also displayed active (phosphorylated) mTORC1 and/or rpS6 (Figure S17A). However, there was a wide range of RON expression, and levels of total RON protein did not appear to correlate with levels of activation of mTORC1/rpS6 proteins as assessed by phospho-specific antibodies. Interestingly, levels of mTORC1 and rpS6 activation also did not necessarily correlate with each other, despite being in the same pathway. We also measured RON and mTORC1 pathway activation in five pairs of matched primary and metastatic lymph node samples. We again detected RON protein frequently, in all except one of the tumor-containing samples, at varying levels (Figure S17B). We assessed the ratio of RON, phosphorylated mTORC1, and phosphorylated rpS6 to the epithelial marker EpCAM and did not see a direct correlation between high levels of total RON protein and levels of activated mTORC1 or rpS6 in primary or metastatic disease. Also, 2/5 cases showed higher levels of RON and mTORC1 pathway activation in metastatic vs primary samples from the same patient (Figure S17C). Thus, our compiled data on a limited number of archived clinical specimens show that RON is expressed at relatively high levels in about half of samples, which is consistent with what is already reported in the literature.^39,40^ Nearly all samples express detectable RON and also display activated mTORC1/rpS6. These data suggest that a large subset of breast cancers could be potential candidates for combined RON/mTOR therapy, although it is likely that other factors contribute to mTORC1 pathway regulation and metastasis in the context of human tumors.

## Discussion

Metastatic breast cancer, regardless of clinical subtype, is a major problem due to the lack of curative therapies. Aberrant expression of the RON receptor tyrosine kinase in breast cancer is associated with poor prognosis, and has a causal role in the development of metastasis,^2,18,23,24^ but the mechanisms by which RON promotes metastasis are largely unknown. In this study, we dissected RON signaling in human breast cancer cells and discovered, for the first time, that mTORC1 is the key downstream mediator of RON-driven metastasis: blocking the mTORC1 pathway using inhibitors, and/or novel RON mutants, prevent RON-dependent colony formation, migration, and metastasis. Also, mutants of RON that hyperactivate the mTORC1 pathway are more metastatic in vivo. Our data also suggests that combining mTORC1 inhibition with RON blockade in the metastatic setting can delay metastatic progression and prolong survival. Interestingly, we previously showed that a truncated isoform of RON, sfRON, promoted metastasis of breast cancer cells through the PI3K pathway, but that this did not depend on AKT or mTOR,^18^ revealing that distinct RON isoforms promote metastasis through different mechanisms. This current work is especially important because many cancers overexpress RON, rather than sfRON.

mTORC1 activity is intimately linked to regulation of cell growth and survival.^34,41^ However, there are few in vitro or in vivo studies that have investigated the causal role of mTORC1/rpS6K in cancer cell migration and/or metastasis.^42–45^ In hepatocellular carcinoma patients, increased activity of mTORC1 has been associated with metastasis and worse outcome. This study, together with another investigation focused on patients with brain metastatic lung adenocarcinoma, showed increased phosphorylation of downstream components of the mTORC1 pathway, such as rpS6, in metastatic tumors compared to the non-metastatic counterparts.^46,47^ In another study, increased incidence of PIK3CA mutations and activation of the PI3K-AKT-mTOR signaling network was reported in breast cancer patients with liver metastasis compared to other metastatic sites.^48^ Our mutation data showed more rpS6 phosphorylation in primary tumors and metastatic lesions from RON mutant A, compared to the non-metastatic RON mutant D or mutant KD, but metastasis was not restricted to the liver.

To our knowledge, there has been no evidence linking RON and mTORC1 pathway components (p70S6K and rpS6) to metastasis. One in vitro study has shown an indirect link between RON and rpS6 in sarcoma cells, but only upon the development of resistance to an IGF1R inhibitor. In that case, RON knockdown was able to restore sensitivity to the IGF1R inhibitor along with a blockade of rpS6 phosphorylation.^49^

mTORC1 has been shown to exert its various functions in cancer through activation by upstream molecules including, but not limited to, TGFβ, MAPK, Src, phospholipase D, and PI3K (the best characterized activator of mTORC1).^31–34,42,50–52^ Our efforts to find the main kinase connecting RON and mTORC1 in human breast cancer revealed PI3K, rather than MAPK, as the dominant upstream kinase. Our RPPA data showed MAPK as the second highest differentially phosphorylated protein upon RON activation, however, we did not find any compelling evidence that MAPK contributes to rpS6 phosphorylation, or that MAPK signaling is important in RON-dependent metastasis in vivo. For example, RON mutant A showed poor activation of ERK and RSK (data not shown), but robust activation of PI3K and mTORC1 pathway components, and was the most metastatic mutant we investigated. In contrast, mutant B clearly activated the MAPK pathway better than mutant A, but was no more metastatic than RON WT. The critical role of PI3K/mTORC1 downstream of RON in metastasis was further supported by the compensatory upregulation of pAKT, as opposed to pERK, in metastatic tumors that developed resistance to everolimus. Indeed, mTORC1 inhibition by rapamycin or rapalogs has been reported to cause activation of PI3K/AKT through suppression of negative feedback loops mediated by mTORC1/p70S6K axis, or activation of ERK through PI3K-mediated stimulation of Ras/Raf cascade. This event, which can happen in the context of any receptor tyrosine kinase, eventually leads to drug resistance.^36–38,53,54^ Our data support feedback activation of AKT concomitant with resistance to everolimus in RON-expressing tumors. Shutting off RON kinase at that point diminished pAKT and slowed metastasis progression.

Rapalogs are currently approved for treatment of ER+ advanced breast cancer, metastatic renal cell carcinoma, subependymal giant cell astrocytoma, and progressive neuroendocrine tumors of pancreatic origin.^55,56^ Although everolimus and other mTORC1 inhibitors have demonstrated statistically significant responses with improved progression-free survival, these responses are usually short-lived and rarely induce sustained disease remission, due to the emergence of resistance. Our findings indicate that, in RON-expressing breast cancers, abrogation of RON signaling upon resistance to everolimus may prolong survival. The majority of breast cancers we examined expressed RON and displayed activated mTORC1/rpS6. Given our data showing ligand-independent activation of RON upon overexpression, it is possible that high RON expression in metastatic breast cancer would stratify patients that may have greater benefit from everolimus and/or RON inhibitor treatment.

In summary, we have made important strides toward determining the key mechanism by which RON signals to promote the metastasis of human breast tumors. Using in vitro and in vivo models, we demonstrated that mTORC1 is the major kinase signaling downstream of RON in both ER+ and TNBC models, and that combined blockade of RON and mTORC1 may offer benefit to breast cancer patients whose tumors overexpress RON and are either at high risk of recurrence or have already progressed to the metastatic stage. This is important because everolimus is already approved in advanced ER+ breast cancer, and because several RON kinase inhibitors have been tested in Phase I clinical trials. So far, RON inhibitors have been well tolerated in healthy subjects (NCT02779738), and in patients with various types of cancer (reference ^57^ and NCT01721148), so this hypothesis could be tested clinically in the near future.

## Methods

### General cell culture

T47D cells stably expressing pHIV-Luc-ZsGreen lentiviral plasmid (gift from Bryan Welm, Huntsman Cancer Institute, Salt Lake City, UT) were previously described.^23^ T47D cells were cultured in RPMI-164 supplemented with 10% heat-inactivated FBS, 1% penicillin/streptomycin, 10 mM HEPES, and 1 mM sodium pyruvate. SUM-159PT cells were cultured in Ham’s F12 containing 5% heat-inactivated FBS, 1% penicillin/streptomycin, 5 μg/ml insulin, and 1 μg/ml hydrocortisone. We used SUM-159PT cells that we previously infected with a lentivirus expressing ZsGreen and luciferase, and sorted for green fluorescence. HCC-1143 cells were purchased from ATCC, and were cultured in RPMI-164 supplemented with 10% heat-inactivated FBS and 1% penicillin/streptomycin. T47D-RON WT and mutants, as well as SUM-159PT-RON stable cell lines, were cultured in the aforementioned media supplemented with 20 μg/ml of blasticidin for selection. T47D-RON cells transduced with shRNA against Raptor were cultured in T47D media in the presence of 3 μg/ml of puromycin for selection. All cell lines were maintained at 37 °C in a humidified incubator with 5% CO_2_.

### Reagents

ASLAN002 (previously described as BMS-777607)^26^ was either provided by ASLAN Pharmaceuticals or purchased from Selleckchem. All other inhibitors (LY2584702, PF-4708671, BI-D1870, Rapamycin, NVP-BKM120, and PD0325901) were purchased from Selleckchem. All drugs were dissolved in DMSO as a 10 mM stock solution and aliquots were stored at −80 °C. Doxycycline was purchased from Sigma-Aldrich and dissolved in sterile Nano-water at a stock concentration of 1 mg/ml. Everolimus was purchased from Selleckchem as lyophilized powder. For in vivo experiments, everolimus was dissolved at 5 mg/ml in ddH_2_O-based solvent consisting of 30% propylene glycol and 5% Tween 80, followed by sonication for a couple of hours until complete dissolving, and stored at −80 °C.

### Site-directed mutagenesis

To generate different RON mutants, specific mutations were introduced at the residues flanking the two essential tyrosines in the multi-substrate docking site (Y1353 and Y1360), using the Quik-Change Site-Directed Mutagenesis Kit (Stratagene). Mutant kinase dead (KD) was generated by generating a K1114M point mutation^16,25^ in the kinase domain (refer to Fig. 4a right panel, for the list of mutations generated from RON wild type). *RON* WT was used as template DNA, and mutations were verified by DNA sequencing. Primers and their complementary strands used to introduce the desired mutations were:

RON-Mut A forward 5′-CATTATGTGCAGATGCCAGCAACCTACATGAACATGGGCCCCAG-3′, RON-Mut A reverse 5′-CTGGGGCCCATGTTCATGTAGGTTGCTGGCATCTGCACATAATG-3′; RON-Mut B forward 5′-CCATTATGTGAATCTGCCGGCGACCTATATGAACCTGGGCCCG-3′, RON-Mut B reverse 5′-CGGGCCCAGGTTCATATAGGTCGCCGGCAGATTCACATAATGG-3′; RON-Mut C forward 5′-ATTATGAGGAGATCCCAGCAACCTACGAGGAGATCGGCCCCAGCAC-3′, RON-Mut C reverse 5′-GTGCTGGGGCCGATCTCCTCGTAGGTTGCTGGGATCTCCTCATAATG-3′; RON-Mut D forward 5′-GACCATTATGTGATCCCGCCAGCAGAGTACCTGGAGTTGGGCCCCA-3′, RON-Mut D reverse 5′-TGGGGCCCAACTCCAGGTACTCTGCTGGCGGGATCACATAATGGTC-3′; RON-Mut KD forward 5′-CCAATGTGCCATCATGTCACTAAGTCGCATCACAG-3′, RON-Mut KD reverse 5′-CTGTGATGCGACTTAGTGACATGATGGCACATTGG-3′

### Generation of lentiviruses, cell transduction and selection, and inducible RON expression

To conditionally express RON WT or mutants under the regulation of doxycycline, each construct was subcloned into pLenti-TRE/rtTA, a tetracycline-inducible (Tet-On) lentiviral expression vector, which was kindly provided by Trudy Oliver, Huntsman Cancer Institute, Salt Lake City, UT. RON WT and mutants were PCR amplified using the following primers including restriction sites for the HpaI and PacI:

SC forward 5′-CGCCTGGAGGTTAACGTCGACGCCACCATGGAGCTC-3′, SC reverse 5′-GGGTGGGTTAATTAAGTCGACTCAAGTGGGCCGAGGAGGCT-3′

PCR products were then subcloned into pLenti-TRE/rtTA vector linearized with HpaI and PacI restriction enzymes, using the Cold Fusion Cloning Kit (System Biosciences), according to the manufacturer’s instructions.

Lentiviral particles were generated by co-transfecting each RON-expressing construct with lentiviral packaging plasmids (pMDLg/pRRE, pCMV-VSV-G, pRSV-Rev) into 293T cells using X-tremeGENE 9 DNA Transfection Reagent. For generation of viral particles from RON WT or different mutants, all constructs were co-transfected with lentiviral packaging plasmids into 293T cells using X-tremeGENE 9 DNA Transfection Reagent. Viral supernatants were harvested 48 and 72 h later, filtered through a 0.45 μm PES filter, and added to T47D-Luc or SUM-159PT cells in the presence of 5 μg/μl polybrene. Cells were incubated overnight. After 2 rounds of infection, cells were selected with blasticidin (20 μg/ml) to generate polyclonal cell lines. Following selection, expression of RON or RON mutants was induced by addition of 25–500 ng/ml of doxycycline for 48 h (depending on the experiment). See Fig. 1a for detailed analysis of inducible expression with doxycycline.

To knock down Raptor in T47D-RON cells, six shRNA constructs in the pLKO.1 lentiviral backbone were purchased from Dharmacon, three of which were chosen to transduce T47D-RON cells following initial screening for effective shRNA activity: sh*Raptor1* (TRCN0000010416), sh*Raptor2* (TRCN0000018342), and sh*Raptor3* (TRCN0000039772). Lentiviral transduction of shRNA constructs into T47D-RON cells was performed as mentioned above, followed by selection with puromycin (3 μg/ml).

### qRT-PCR

Total RNA from T47D-RON WT and mutants was extracted with the Qiagen RNeasy Mini kit (Qiagen), according to the manufacturer’s instructions. 500 ng of purified RNA from each sample was reverse transcribed using SuperScript first-strand synthesis system for RT-PCR (Invitrogen). About 50 ng of cDNA was used in a 20 µl reaction including specific primers for target genes and subjected to real-time PCR in triplicates, using the LightCycler 96 System. The following primer sets were used to amplify specific target genes: RON forward 5′-GAGTCATTGGCAAAGGCCAC-3′, reverse 5′-ATCTCTGTGATGCGACTTAGT-3′; GAPDH forward 5′-ATCATCCCTGCCTCTACTGG, reverse 5′-GTCAGGTCCACCACTGACAC.

### Inhibition of signaling pathways

For in vitro evaluation of signaling pathways, RON or RON-mutant-expressing cells were cultured in the presence of doxycycline for 48 h, followed by treatment with different inhibitors for 4 h. MSP stimulation was done in serum-starved conditions, where cells were cultured in media with 0% serum supplemented with 1% BSA for 24 h, before MSP stimulation for 30 min.

### Clonogenic assays

T47D-RON, T47D-RON-sh-Scramble, and T47D-RON-sh-Raptor (construct #3) cells were seeded at a density of 270 cells per 6-well plate, in triplicate, in the presence and absence of 500 ng/ml doxycycline. To evaluate the effect of pharmacologic mTORC1 inhibition on colony formation, T47D-RON cells were treated with Rapamycin (50 nM) every other day in the presence of doxycycline. Cells were allowed to form colonies for 4 weeks. Colonies were stained with 0.5% crystal violet, and number and total areas of colonies were determined by ImageJ software (NIH), version 1.46r.^58,59^

### Cell migration/wound healing assays

Real-time cell migration assays were performed using an Incucyte instrument (Essen BioScience, MI, USA). T47D-RON, T47D-RON-sh-Scramble, and T47D-RON-sh-Raptor (construct #3) were grown to confluence in 96-well ImageLock microplates (Essen BioScience) in the presence and absence of 500 ng/ml doxycycline. A scratch was made using the 96-pin WoundMaker™ the next day, followed by washing with PBS to remove any cell debris. To assess the effect of pharmacologic mTORC1 inhibition on migration, T47D-RON cells were treated with 50 nM Rapamycin every other day in the presence of doxycycline. Phase images were automatically taken every 2 h by the IncuCyte™ software. Scratch wound data were analyzed by the IncuCyte software and results were reported as wound width vs time.

### Immunoblotting and immunoprecipitation

For Western blotting, cells or tumors were lysed in RPPA lysis buffer (1% Triton X-100, 50 mM HEPES, pH 7.4, 10% glycerol, 150 mM NaCl, 1.5 mM MgCl_2_, and 1 mM EGTA), which was freshly supplemented with 100 mM NaF, 10 mM Na pyrophosphate, 1 mM Na_3_VO_4_, 2 mM DTT, 1× protease inhibitor (Sigma-Aldrich, 4693124001), and 1× phosphatase inhibitor cocktails 2 and 3 (Sigma-Aldrich, P5726 and P0044) for 30 min on ice. Tumors were lysed using an electric homogenizer in this buffer. Lysates were centrifuged at 4 °C for 20 min at 14,000 rpm, followed by protein quantification (BCA assay, Bio-Rad Laboratories).

For immunoprecipitations, 200 µg of whole-cell lysate was diluted in IP buffer from Pierce (25 mM Tris–HCl, pH 7.4, 150 mM NaCl, 1 mM EDTA, 1% NP40, and 5% glycerol) with freshly added protease and phosphatase inhibitor cocktails (see above). Immunoprecipitation was done using 30 µl of clone 4G10 anti-phosphotyrosine agarose conjugate (EMD Millipore, 16-101) in total IP volume of 200 µl. This mixture was incubated with rocking overnight at 4 °C, followed by centrifugation and washing with IP buffer 3 times.

For Western blot analysis, equal amounts of protein (generally 100 µg) were loaded and separated with 10–12% SDS-PAGE under reducing conditions, and transferred to PVDF membrane (EMD Millipore, IPVH00010). The membranes were incubated with antibodies in 5% milk (in TBST buffer) overnight at 4 °C. The following antibodies were purchased from Cell Signaling Technology and used at specified dilutions: phospho Akt Ser473 (1:1000, #9271); pan Akt (1:1000, #4691); phospho ERK Thr202/Tyr204 (1:2000, #4370); pan ERK (1:2000, #4695); phospho Src Tyr416 (1:1000, #2113); Src (1:1500, #2110); phospho PLCγ1 Tyr783 (1:500, #2821); PLCγ1 (1:1000, #2822); phospho S6 Ser235/236 (1:1000, #2211); S6 (1:500, #2317); phospho P70S6K Thr 389 (1:1000, #9205); P70S6K (1:1000, #2708); phospho RSK Ser 380 (1:1000, #11989); RSK (1:1000, #9355); phospho mTOR Ser 2448 (1:1000, #2971); mTOR (1:1000, #2983); EpCAM (1:1000, #14452); and Raptor (1:1000, #2280). Phospho PDCD4 Ser 457 (Abcam, ab74141, 1:1000) and PDCD4 (Cell Signaling Technology, 1:1000, #9535) were kind gifts from Katharine Ullman, Huntsman Cancer Institute, Salt Lake City, UT. Other antibodies used were phospho RON Tyr1238/1239 (1:200, R&D #AF1947); RON (1:400, Santa Cruz #sc-322); and GAPDH (1:1000, Santa Cruz #sc-32233). Anti-rabbit IgG (1:5000, #sc-2305) or anti-mouse IgG (1:5000, #sc-2314) secondary antibodies conjugated with horse radish peroxidase were from Santa Cruz Biotechnology. Targeted proteins were developed with Clarity™ Western ECL Substrate (Bio-Rad), and chemiluminescence signals were captured with the ChemiDoc XRS system using Image Lab Software. A vertical line indicates if lanes were not adjacent on the original image (e.g., if another sample or marker lane was in between and cropped out).

### RPPA

RPPA experiments were done in accordance with instructions from the RPPA Core facility at the MD Anderson Cancer Center. Cells grown under the specified conditions were washed twice with cold PBS and lysed in RPPA lysis buffer for 30 min on ice. Protein concentrations were determined by BCA assay and adjusted to 1.5 µg/µl with 4× SDS sample buffer (40% glycerol, 8% SDS, 0.25 M Tris–HCl, pH 6.8, with freshly added 2-mercaptoethanol at 1:10 of the volume). Samples from three separate replicates for each group were submitted to the RPPA core facility at MD Anderson Cancer Center, University of Texas, to be analyzed for 305 validated targets. Linear values of normalized RPPA data were used to determine fold changes.

### Animal studies

All animal procedures were reviewed and approved by the University of Utah IACUC committee, and performed in accordance with established guidelines. All animals were handled in strict accordance with good animal practice, and maintained according to the standards of pathogen-free conditions at Huntsman Cancer Institute. To examine spontaneous metastasis of mammary tumors arising from T47D-Luc-RON WT and RON mutants, 5 × 10^6^ cells from each group were suspended in 25 μl matrigel and implanted into the cleared right inguinal mammary fat pad of 4–6-week-old female NOD/SCID mice (Jackson Laboratory stock #1303) using our routine procedures.^60^ To examine the effect of hMSP on metastasis, 5 × 10^6^ cells from T47D-Luc-RON WT and selected mutants were injected orthotopically into 4–6-week-old female NOD/SCID mice expressing hMSP knocked in to the endogenous mouse MSP locus (a kind gift from AVEO Pharmaceuticals).

For all experiments, estrogen pellets^60^ were simultaneously implanted into the contralateral mammary fat pad during surgery, and mice were put on 50 ppm γ-irradiated Doxy diet (Mod LabDiet 5053, 1816292-203) to induce expression of RON WT or mutants. Tumor volume was measured with Vernier calipers, until the study endpoint when tumors reached approximately 1300 mm^3^. To analyze metastasis at the endpoint, mice were anesthetized and given 150 mg/kg of D-luciferin (Gold Biotechnology, LUCK-1G) by IP injection. Five minutes later, mice were euthanized, organs were harvested, and ex vivo luciferase activity was measured by IVIS imaging and LivingImage analysis software (IVIS Spectrum, Caliper LifeSciences). Organs that were positive for luciferase were further validated by histology. Primary tumors were snap-frozen in liquid nitrogen and/or fixed in 10% neutral-buffered formalin (NBF) (Thermo Fisher Scientific, 22-050-105) for further molecular and histologic analysis respectively.

For experimental metastasis studies, 2 × 10^6^ luciferase expressing T47D-RON cells were resuspended in HBSS, and injected into the lateral tail vein of ~6-week-old female NSG mice (Jackson Laboratory stock #5557) which, as described above, also received estrogen pellets during the same procedure. Mice were put on Doxy diet to induce expression of RON and imaged weekly for luciferase activity. Treatment with everolimus (5 mg/kg, once daily in a repeating cycle of 5 days on, 2 days off) or vehicle was via oral gavage and started at day 30 following cell injection, when metastatic lesions were detectable with in vivo IVIS imaging. Mice were monitored for body weight on a regular basis. Animals were imaged weekly (3 min exposure for max bioluminescence detection) over the course of treatment with everolimus or vehicle, and luciferase activity was quantified as total flux (photons/s) for further analysis. At the end of the experiment, mice were anesthetized and injected with D-luciferin as described above, and organs were subjected to ex-vivo IVIS imaging. Positive organs for metastasis were collected and fixed in 10% neutral-buffered formalin for further H&E staining and IHC analysis.

### Immunohistochemistry (IHC)

Harvested tumors or tissues were fixed in 10% NBF upon necropsy and embedded in paraffin. In order to detect metastatic foci, we performed hematoxylin–eosin staining according to our standard protocols.^61^ To characterize metastatic lesions in cohorts of mice, IHC was performed. For most antibodies, antigen retrieval was performed by boiling in 10 mM sodium citrate buffer pH 6.0 for 20 min. Pan-cytokeratin required antigen retrieval with 0.1% Trypsin for 15 min. After blocking endogenous peroxidases, the sections were incubated with blocking solution (10% goat serum with 5% BSA for rabbit antibodies, or 10% normal human serum with 5% BSA and MOM kit reagents (mouse IgG blocking reagent and Avidin) for mouse antibodies) followed by incubation with primary antibodies for either 1 h at room temperature or overnight at 4 °C. The following antibodies were used: human-specific pan-cytokeratin, clone CAM5.2 (1:50, BD Biosciences #349205), RON (1:50, Santa Cruz #sc-322), phospho S6 Ser235/236 (1:400, Cell Signaling Technology #2211), phospho AKT Ser473 (1:100, Cell Signaling Technology #4060), phospho ERK Thr202/Tyr204 (1:100, Cell Signaling Technology #9661). Sections were then washed in 0.5% Tween-PBS and incubated with secondary antibody (biotinylated anti-mouse IgG (M.O.M., Vector Laboratories), or EnVision+System-HRP anti-Rabbit (DAKO)) for 30 min at room temperature. Staining was visualized by 3,3′-diaminobenzidine, with hematoxylin as a counter-stain. Slides were imaged on an Olympus Bx50 microscope with a Canon EOS Rebel XSI camera using EOS imaging software.

### Analysis of human breast cancer samples

Human specimens were collected from patients with informed consent under approved University of Utah IRB protocols #89989 and #10924 and were de-identified. Tissues were homogenized in RPPA lysis buffer to isolate protein. 100 μg of protein was analyzed by Western blotting with specific antibodies as described above, as well as anti-EpCAM to control for epithelial cell content.

### Statistics

All in vitro experiments were performed three separate times and in triplicate when applicable. Statistical analysis was performed using 1-way ANOVA when more than 2 groups were compared, and unpaired 2-tailed Student’s *t* test when only 2 groups of data were concerned. The details of each statistical test used for each experiment is described in the figure legends. All statistical analysis was performed using GraphPad Prism 7.0 Software. *P* < 0.05 was considered statistically significant.

## Electronic supplementary material

Supplemental Figures

Raw RPPA data

## Data Availability

The entire set of RPPA data is available in Supplementary Information. All additional data is available from the corresponding author upon reasonable request.

## Electronic supplementary material

**Supplementary information** accompanies the paper on the *npj Breast Cancer* website (10.1038/s41523-018-0091-5).
