# Supplementary material for: mTORC1 is a key mediator of RON-dependent breast cancer metastasis with therapeutic potential
Source: NPJ Breast Cancer. 2018 Nov 9;4:36. doi: 10.1038/s41523-018-0091-5 (PMC6226524; doi:10.1038/s41523-018-0091-5)
Supplement: Supplementary file 1 — Supplemental Figures [file 41523_2018_91_MOESM1_ESM.pdf]

Supplementary Figure S1

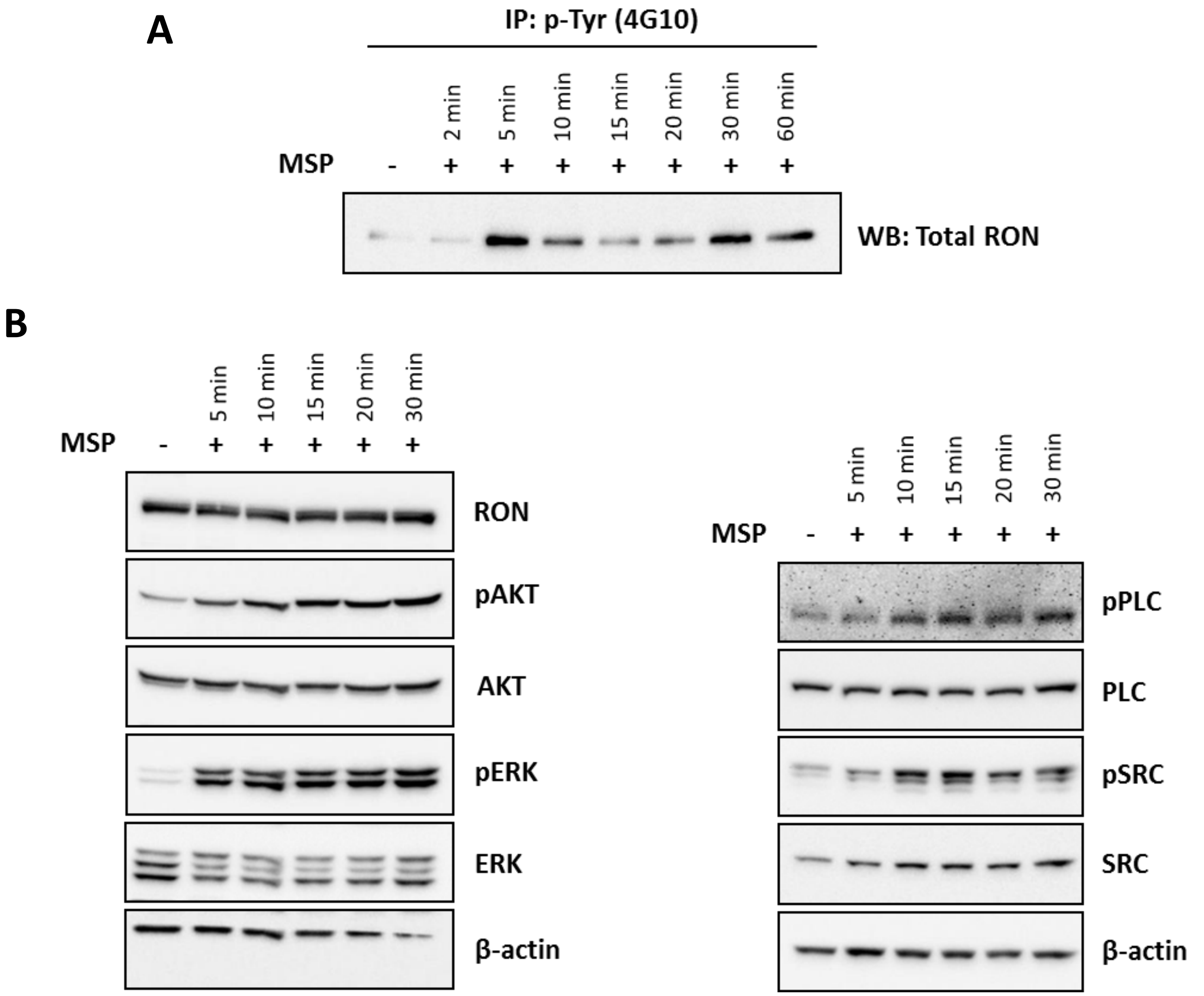

Supplementary Figure S1. kinetic analysis of RON activation following MSP stimulation and the effect on downstream signaling. A) Immunoprecipitation (IP)–Western blot analysis showing kinetic activation of RON by MSP. T47D-RON cells were treated with 100 ng/ml of doxycycline for 48 h, and were serum starved for 24 h followed by MSP stimulation (100 ng/ml) for different time points. Cell lysates from each time point were immunoprecipitated with 4G10 antibody and analyzed by Western blot. B) Whole cell lysates of the same samples were analyzed for activation of different signaling pathways downstream of RON. β-actin was used as loading control.

Supplementary Figure S2

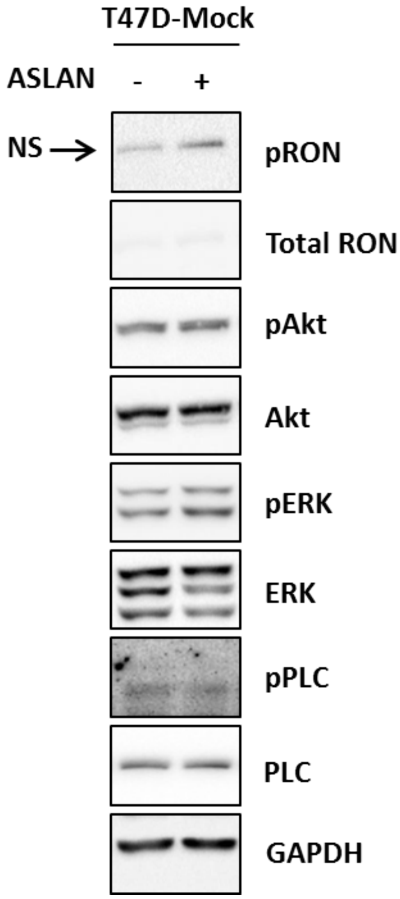

Supplementary Figure S2. Effect of small molecule RON inhibitor, ASLAN002, on T47D-Mock and downstream signaling. Western blot showing status of different signaling pathways in T47D-Mock cells in the absence and presence of ASLAN002 for 4 h. As is indicated, 1 uM of ASLAN002 used to inhibit RON activity in T47D-RON cells shows no off-target effect on activity status of different signaling pathways when RON is not present. GAPDH was used as loading control. NS, non-specific band.

Supplementary Figure S3

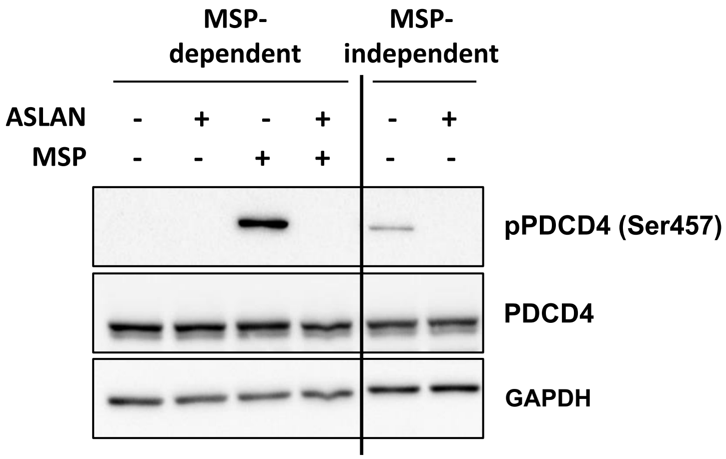

Supplementary Figure S3. Validation of RPPA analysis in T47D-RON cells in MSP-dependent and –independent conditions. Western blot shows phosphorylation of PDCD4 tumor suppressor in response to MSP stimulation or RON overexpression. GAPDH was used as loading control. Line indicates separation between lanes on the same Western blots.

Supplementary Figure S4

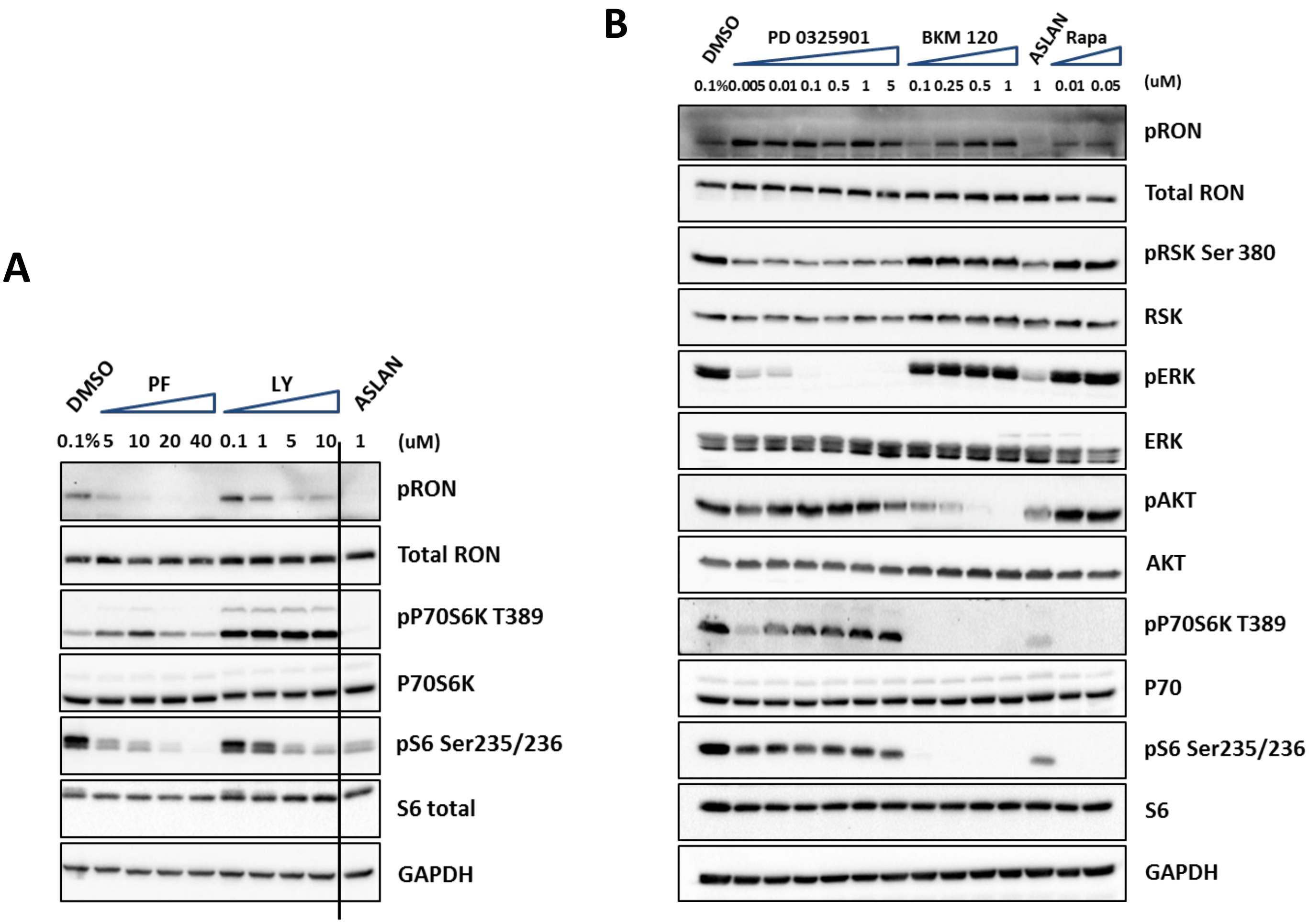

Supplementary Figure S4. Analysis of MSP-independent RON signaling using inhibitors of p70S6K, PI3K, and MAPK pathways. A) Representative Western blots for analysis of p70S6K as a potential kinase upstream of rpS6 using two inhibitors: pan-p70S6K (LY2584702) and p70S6K1 (PF-4708671). B) Western blot analysis showing PI3K, rather than MAPK, is the dominant kinase downstream of RON and upstream of mTORC1 responsible for phosphorylation of rpS6 in T47D-RON cells. Inhibition of PI3K or MAPK signaling for 4 h using different doses of NVP-BKM120 and PD0325901 respectively, shows that PI3K inhibition causes complete abrogation of rpS6 phosphorylation downstream of RON. See also Supplementary Figure S5 for signaling analysis using these inhibitors in the context of MSP-induced RON activation in T47D-RON cells. Line indicates separation between lanes on the same Western blots.

Supplementary Figure S5

B

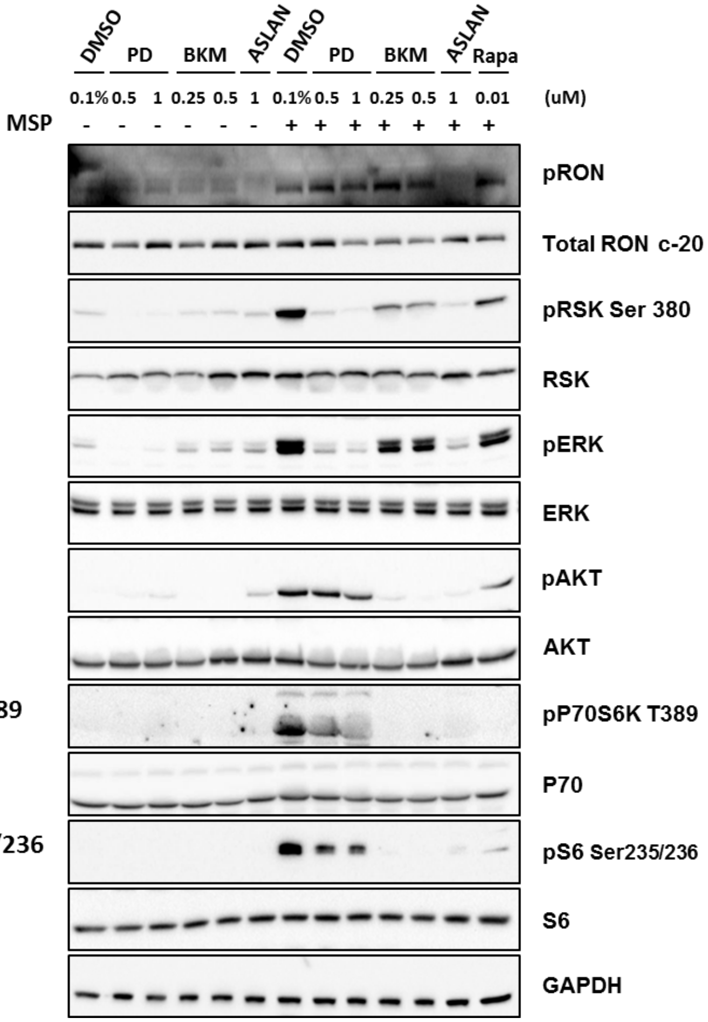

A

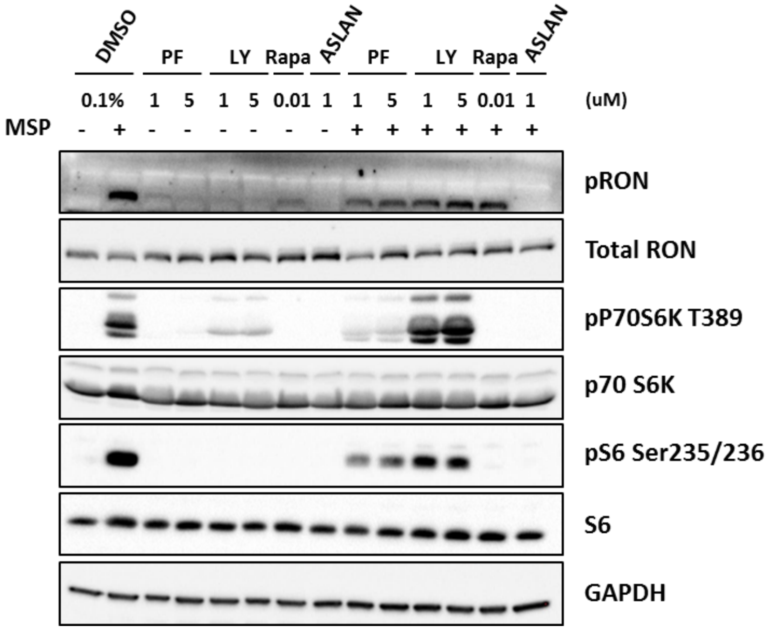

Supplementary Figure S5. Analysis of MSP-induced RON signaling using inhibitors of p70S6K, RSK, mTORC1, PI3K, and MAPK pathways. A) Representative Western blot showing effect of different kinase inhibitors on phosphorylation of rpS6 in the context of MSP-induced RON activation. T47D-RON cells were cultured in the presence of doxycycline (50 ng/ml) for 48 h and in serum starved media for 24 h, followed by 4 h treatment with different inhibitors. Cells were then stimulated with MSP (100 ng/ml) for 30 min. Inhibition of mTORC1 by rapamycin caused complete inhibition of phospho-rpS6, which is mediated through inhibition of p70S6K. PF-4708671, as a specific inhibitor of p70S6K1, shows partial efficacy on inhibiting rpS6 phosphorylation downstream of RON. B) Western blot analysis showing PI3K as the dominant kinase downstream of RON and upstream of mTORC1 responsible for phosphorylation of rpS6. Doxycycline-treated T47D-RON cells (50 ng/ml, for 48 h) were serum starved for 24 h and treated with NVP-BKM120 (PI3K inhibitor) and PD0325901 (MAPK inhibitor) for 4 h, followed by MSP stimulation (100 ng/ml) for 30 min. GAPDH was used as loading control.

Supplementary Figure S6

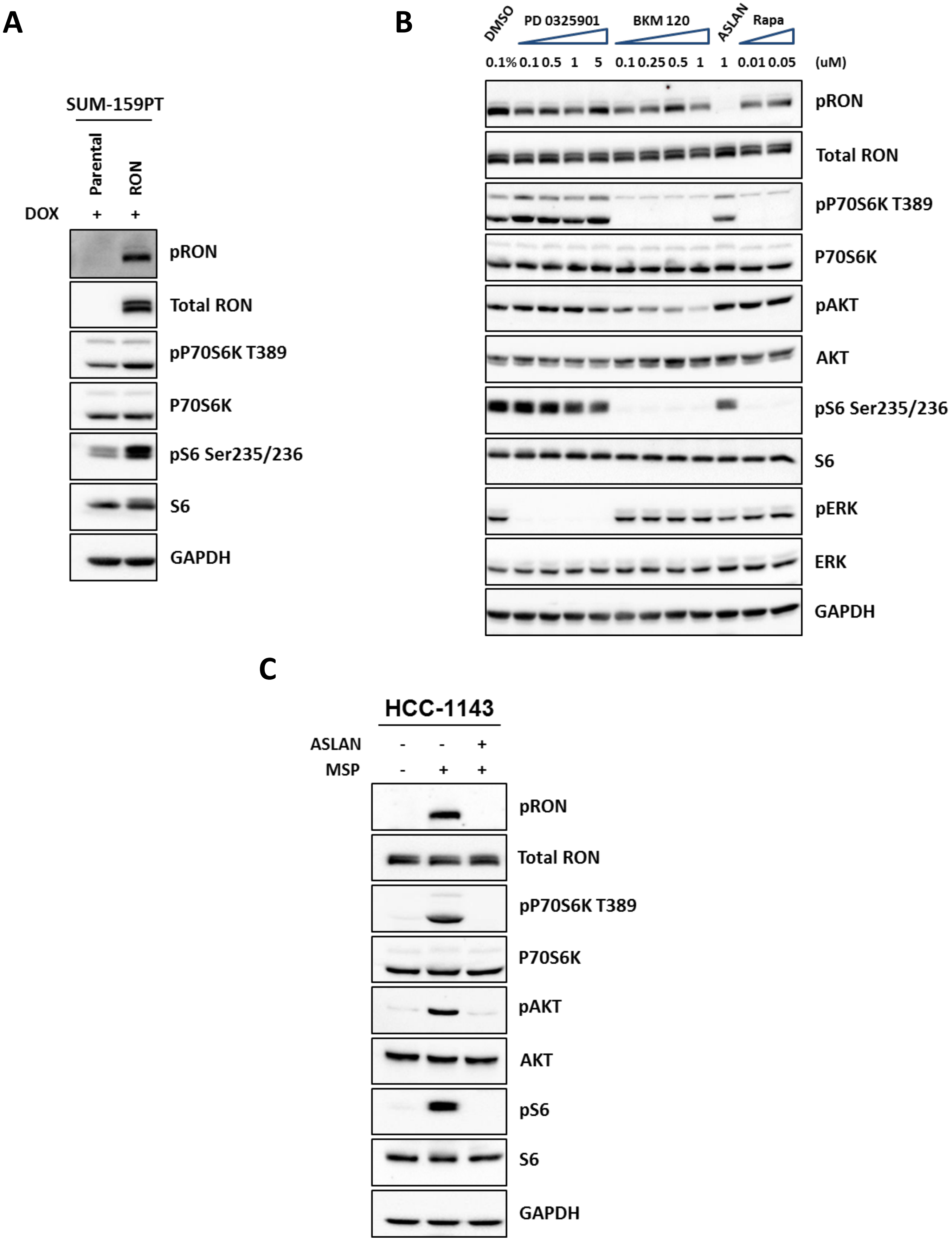

Supplementary Figure S6. RON signals through the PI3K/mTORC1 pathway in TNBC cell lines regardless of type of activation, similar to T47D ER+ cell line. A) Representative Western blots showing status of mTORC1 pathway components in SUM-159PT cells engineered for RON overexpression. SUM-159PT cells were infected with RON in the Tet-inducible lentiviral backbone, and treated with 500 ng/ml of doxycycline for 48 h. Western blot shows increased phosphorylation of p70S6K and rpS6 upon overexpression of RON. B) Signaling analysis of SUM-159PT-RON cells using inhibitors of PI3K, MAPK, and mTORC1 pathway. SUM-159PT-RON cells were cultured in the presence of doxycycline (500 ng/ml) for 48 h, followed by 4 h treatment with NVP-BKM120, PD0325901, and rapamycin, as inhibitors of PI3K, MAPK, and mTORC1 pathways respectively. Results show PI3K is the main kinase upstream of mTORC1, responsible for phosphorylation of rpS6 in a MSP-independent condition, similar to T47D-RON cells. C) Signaling analysis of HCC-1143 TNBC cells with high endogenous level of RON in the presence or absence of MSP. HCC-1143 cells were serum starved for 24 h, and treated with RON inhibitor ASLAN002 for 4 h followed by MSP stimulation (100 ng/ml) for 30 min. Western blot shows robust activation of AKT, p70S6K, and rpS6 as a result of RON activation, which is reversed by ASLAN002. GAPDH was used as loading control.

Supplementary Figure S7

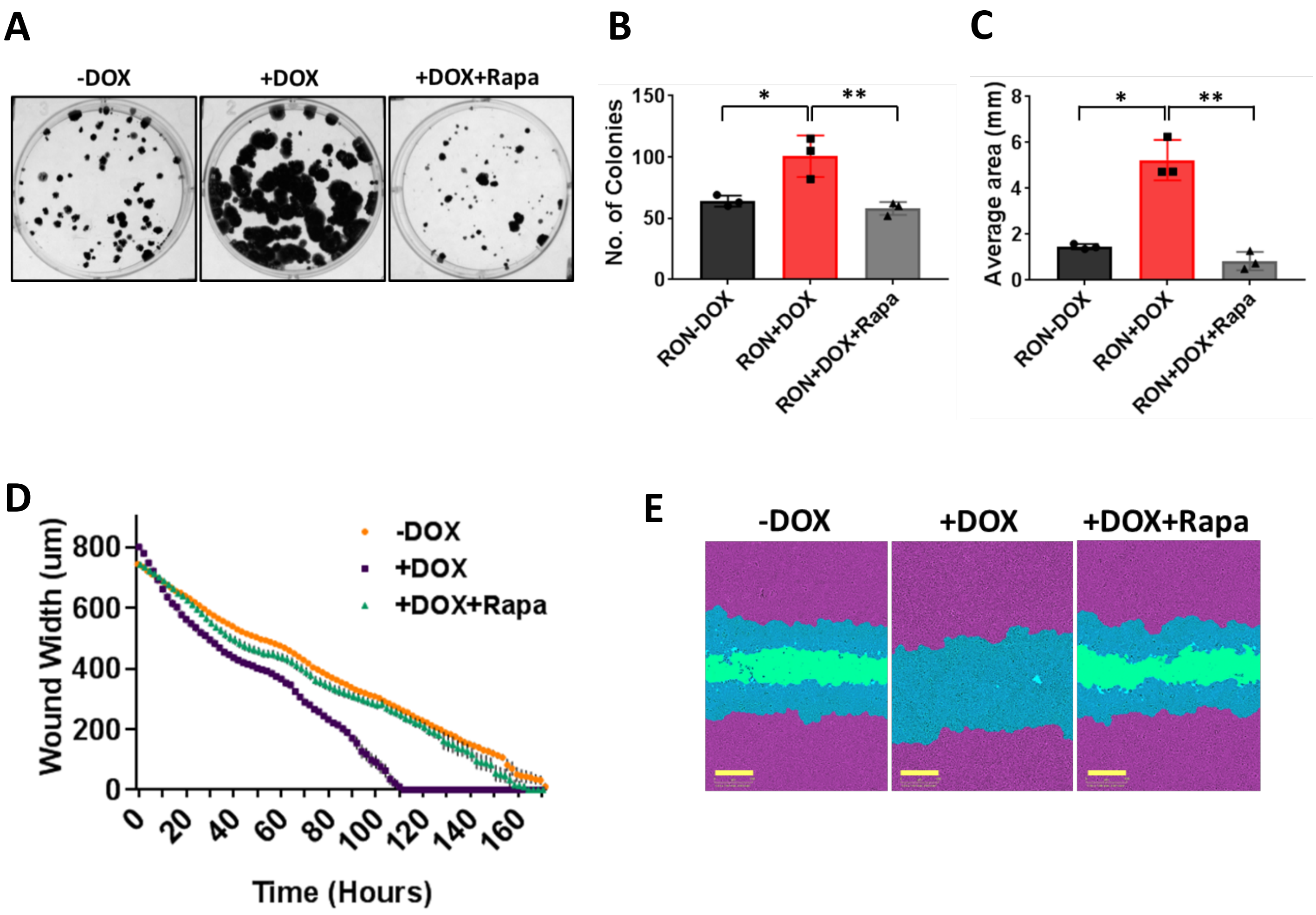

Supplementary Figure S7. mTORC1 is required for RON-mediated colony formation and migration in T47D-RON cells. A-C) Effect of mTORC1 inhibition on colony formation of T47D-RON cells. Doxycycline-treated (500 ng/ml) T47D-RON cells were seeded at very low density, treated ± Rapamycin (50 nM) every other day and were allowed to form colonies, followed by Crystal Violet staining. Representative images are shown in panel A. Number and average area per colony, quantified by ImageJ are shown in panel B & C. D and E) Effect of mTORC1 inhibition on migration of T47D-RON cells. Doxycycline-treated (500 ng/ml) T47D-RON cells were seeded at high density the day before wounding, and were treated with Rapamycin (50 nM) every other day. The rate of wound closure in each group over the course of treatment is shown in panel D. Representative images at day 5 is shown in panel E. The initial wound in each group is shown in blue/green; migrating cells from the initial wound are shown in blue. Scale bars represent 300 µm. Data are shown as mean ± SEM, n=7.

Supplementary Figure S8

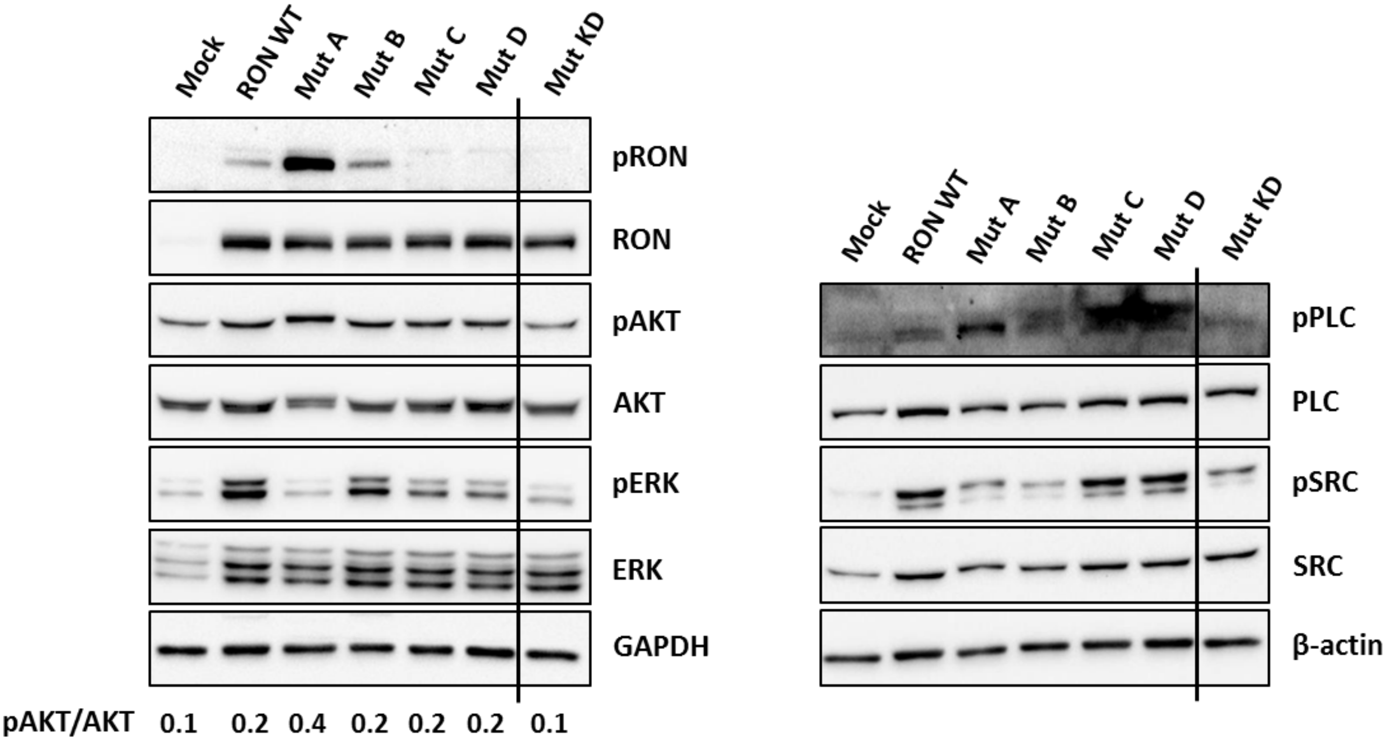

Supplementary Figure S8. Signaling analysis of RON wild type and mutants in T47D cells. T47D cells infected with RON WT and different mutants were treated with 100 ng/ml of doxycycline for 48 h and cultured in serum starved media for 24 h. Differential activation of downstream signaling pathways is shown among RON mutants. The ratio of pAKT to AKT is shown for different mutants, which indicates more AKT activation in Mut A compared to other RON mutants. GAPDH and β-actin were used as internal controls.

Supplementary Figure S9

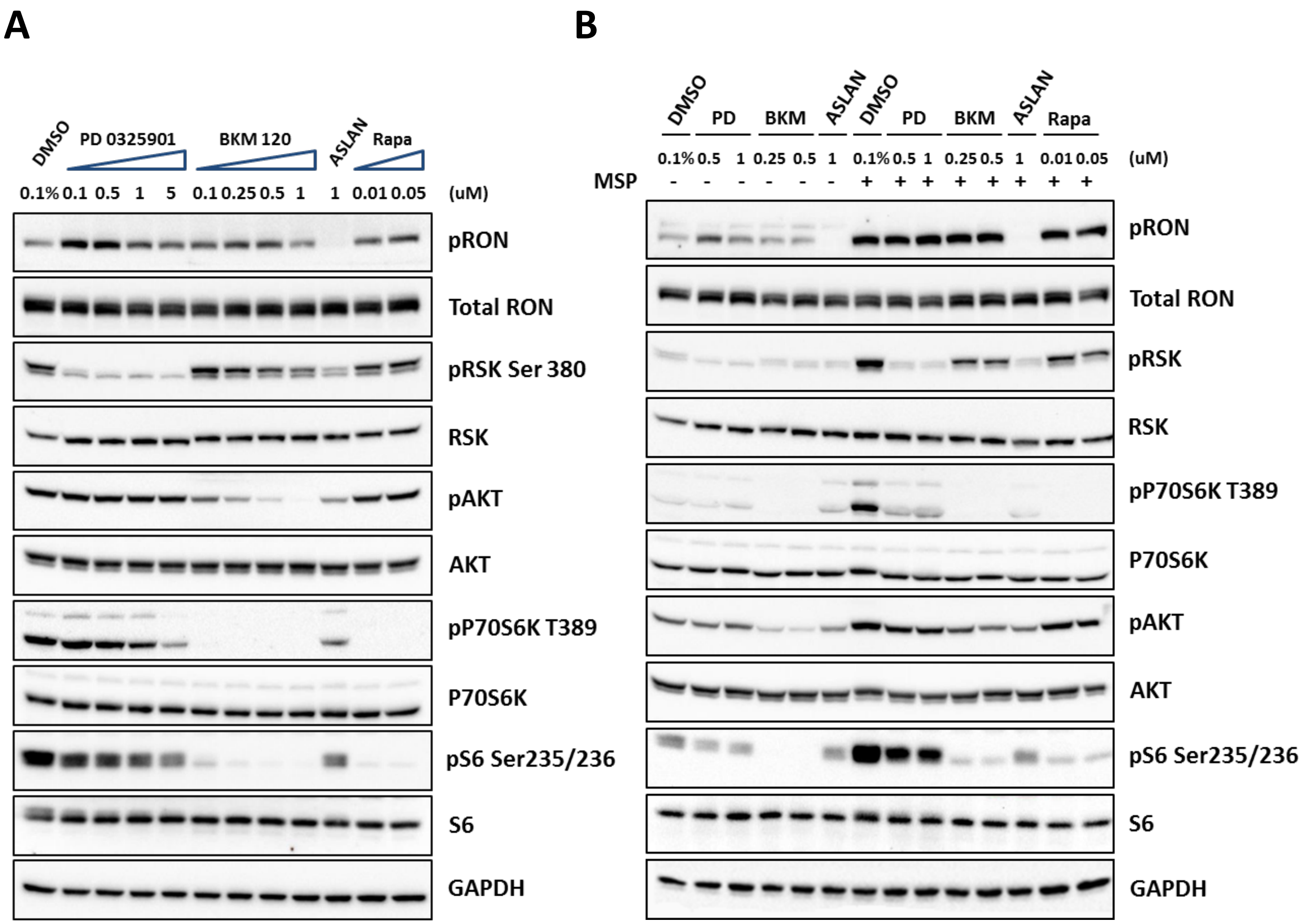

Supplementary Figure S9. RON-Mut A Signals strongly through mTORC1 downstream of PI3K in both MSP-dependent and –independent types of RON activation. A) T47D-RON-Mut A cells were cultured in the presence of doxycycline (500 ng/ml) for 48 h, followed by 4 h treatment with NVP-BKM120 and PD0325901, as inhibitors of PI3K and MAPK pathways respectively. As is shown, RON-Mut A signals strongly through p70S6K/rpS6 which is fed by mTORC1 downstream of PI3K. B) Representative Western blots showing PI3K inhibition in T47D-RON Mut A cells causes dramatic decrease of rpS6 phosphorylation downstream of mTORC1. T47D-RON Mut A cells were cultured in the presence of doxycycline (50 ng/ml) for 48 h, and in serum-starved media for 24 h. Cells were then treated with various doses of inhibitors for 4 h, followed by MSP stimulation (100 ng/ml) for 30 min. GAPDH was used as loading control.

Supplementary Figure S10

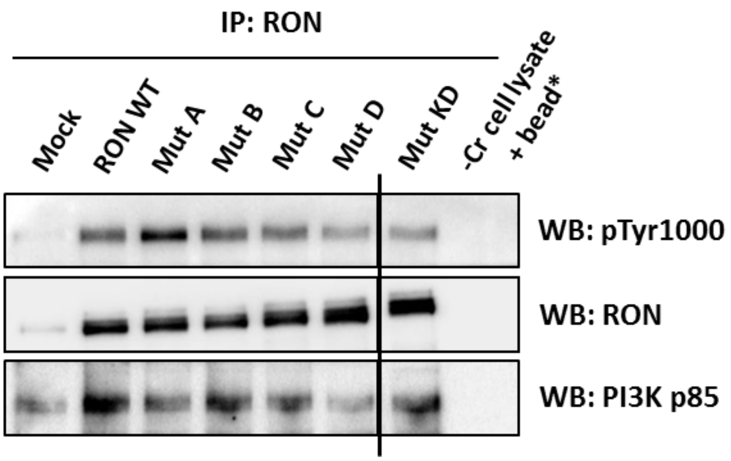

Supplementary Figure S10. Co-immunoprecipitation analysis of RON wild type and mutants in T47D cells. T47D cells infected with RON WT or different mutants were treated with 100 ng/ml of doxycycline for 48 h and cultured in serum starved media for 24 h. Lysates were immunoprecipitated with RON antibody and probed for PI3K p85, the regulatory subunit of PI3K. Phosphorylation and expression status of RON among the mutants are shown by pTyr1000 and RON antibodies.

Supplementary Figure S11

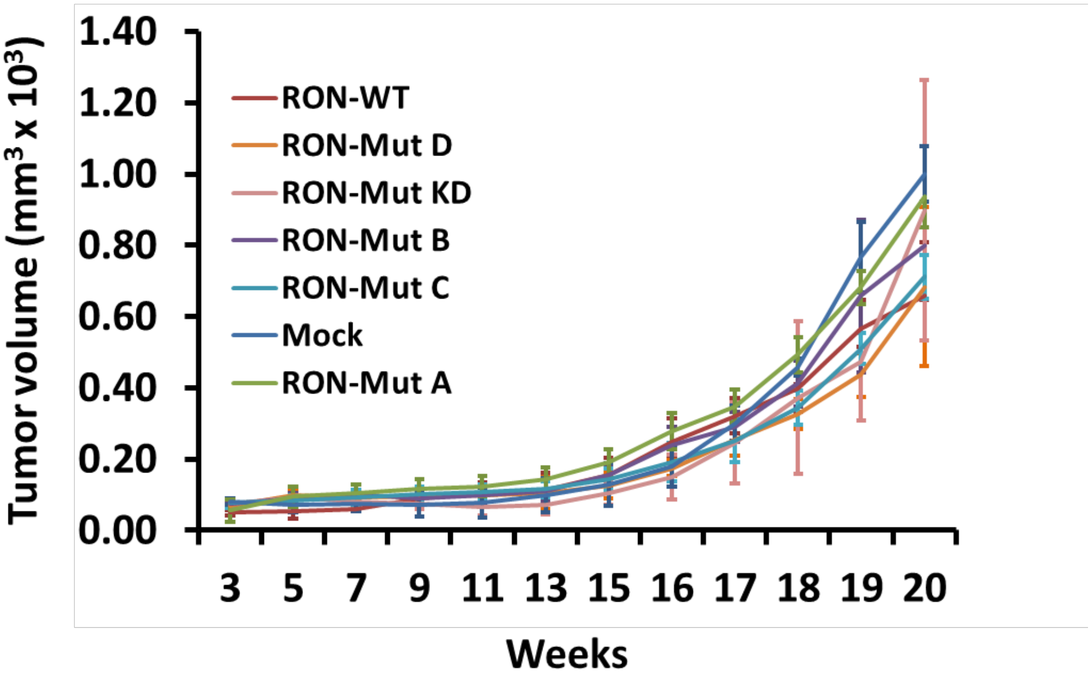

Supplementary Figure S11. Growth rate of T47D tumors expressing various RON mutants in NOD-SCID mice. Graph represents growth rate of tumors from T47D-RON WT and different mutants following orthotopic transplantation into NOD/SCID mice.

Supplementary Figure S12

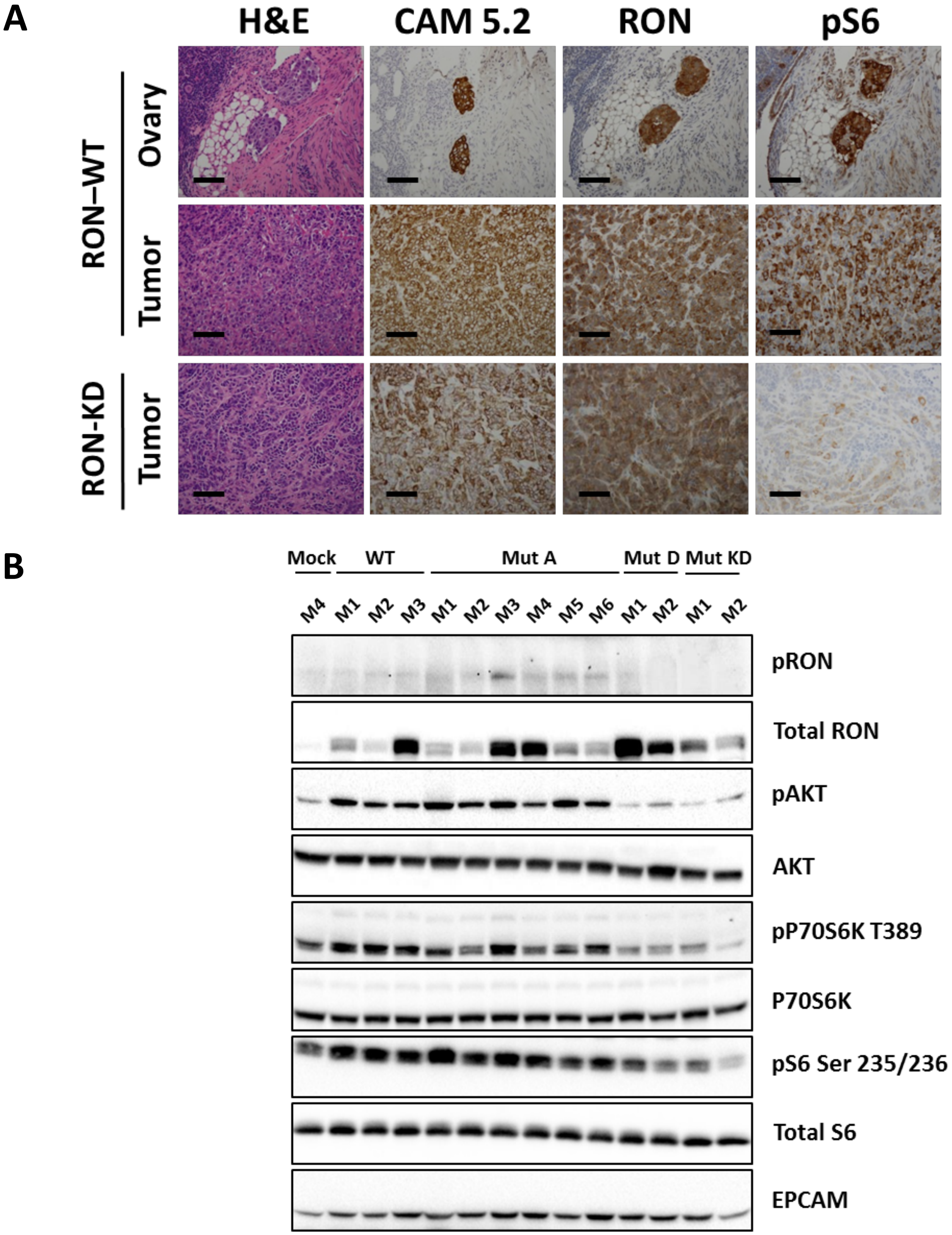

Supplementary Figure S12. Phosphorylation status of rpS6 and components of the PI3K/mTORC1 pathway in T47D tumors expressing RON WT or mutants. A) Representative images showing H&E, CAM5.2, RON and pS6 (Ser 235/236) staining for metastatic RON-WT (ovary metastasis and primary tumor) vs non-metastatic RON-Mut KD (primary tumor) following orthotopic transplantation into NOD/SCID mice. Scale bars represent 100 µm. B) Western blot shows status of pAKT, pP70 and pS6 in lysates from primary tumors of T47D-RON WT, RON- Mut A, Mut D and Mut KD. EPCAM was used as loading control, as an epithelial marker for human tumors.

Supplementary Figure S13

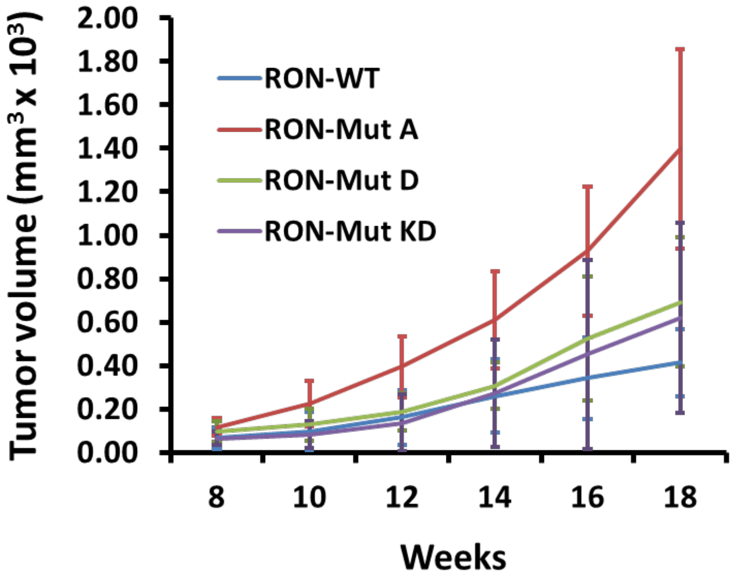

Supplementary Figure S13. Tumor growth rate of RON WT and mutants in hMSP-NOD-SCID mice. Graph represents growth rate of tumors from T47D-RON WT, RON Mut A, Mut D, and Mut KD following orthotopic transplantation into hMSP-NOD/SCID mice (n= 6-8 per group). The difference in tumor growth rate between different groups was not statistically significant (One-Way ANOVA, Multiple comparisons).

Supplementary Figure S14

A

	WT	Mut A	Mut D	Mut KD
Tumor volume average (mm^3)	1408.0	1487.0	1385.0	1493.0
Overall Metastasis	6/8 (75%)	8/8 (100%)	1/6 (16.7%)	1/6 (16.7%)

B

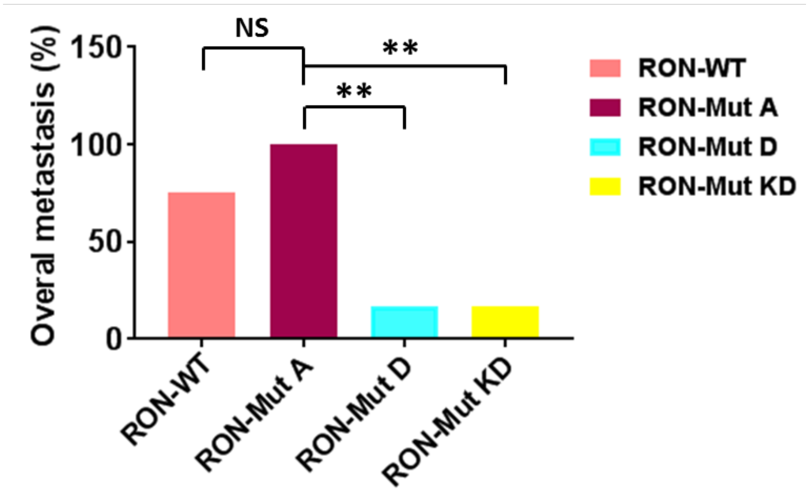

Supplementary Figure S14. Effect of hMSP on metastatic burden from tumors expressing RON WT or mutants. A) Average tumor volume at the endpoint, number of mice in each group, and overall metastasis frequency from tumors expressing different RON mutants in hMSP mice are provided. B) Quantification of overall spontaneous metastasis in hMSP-NOD/SCID mice following transplantation of RON WT, RON Mut A, Mut D, and Mut KD is shown. ***P*<0.005, NS, non-significant (Chi-square analysis, Fisher's exact test).

Supplementary Figure S15

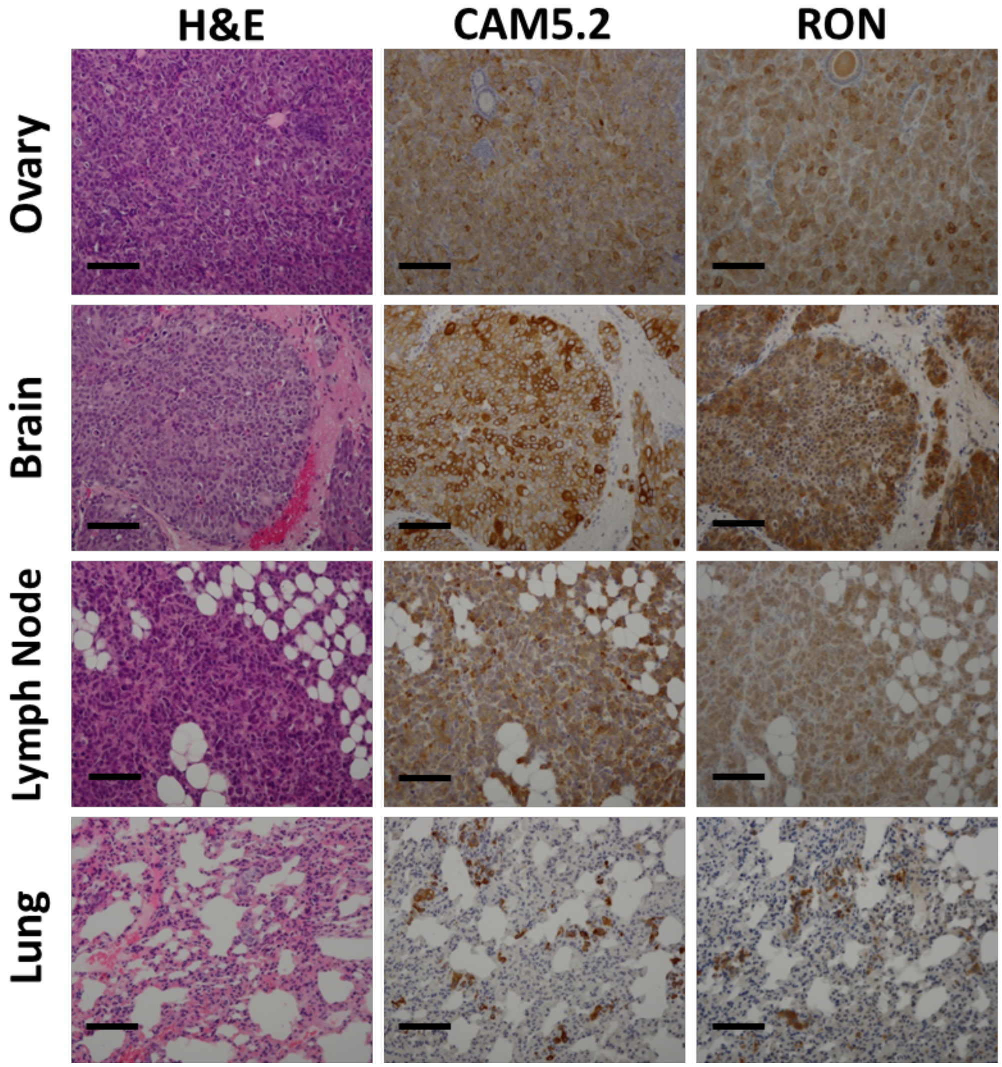

Supplementary Figure S15. Tissue tropism of metastasis in NSG mice following tail vein injection of T47D-RON cells as an experimental metastasis model. Representative images showing H&E, RON, and CAM5.2 staining on metastatic lesions in different organs of NSG mice following tail vein injection of T47D-RON cells. Mice were put on doxy diet (50 ppm) upon injection of cells to induce RON expression. Scale bars represent 100 µm.

Supplementary Figure S16

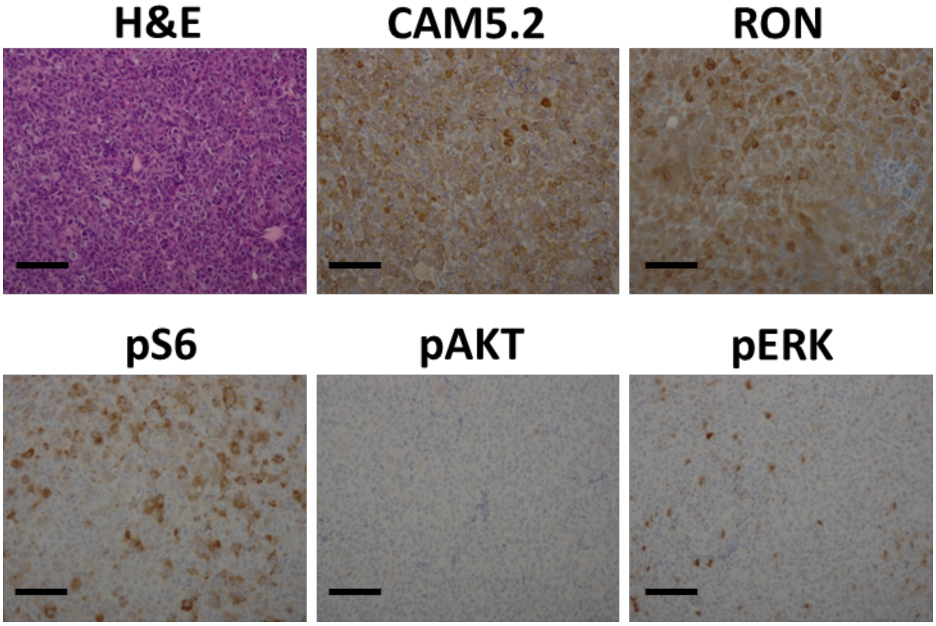

Supplementary Figure S16. Phosphorylation status of AKT and ERK in metastatic lesions induced by T47D-RON cells in vehicle-treated mice. Representative images indicate basal phosphorylation level of rpS6, AKT, and ERK in metastatic ovaries of mice treated with vehicle. Treatment with vehicle started at day 30 following tail vein injection of T47D-RON cells and continued until the end of the experiment. Representative images are from mice euthanized at week 4 of treatment. Scale bars represent 100 µm.

Supplementary Table S1.

Protein name	-MSP	+MSP	+MSP +ASLAN	*p* -value
rpS6-pS235/S236	0.52	1.58	0.42	0.00035
rpS6-pS240/S244	0.59	1.43	0.38	0.000447
MAPK-pT202/Y204	0.95	2.20	0.92	0.000447
PDCD4	0.95	1.67	0.60	0.010733
Akt-pS473	1.10	1.69	0.94	0.016835
p90RSK-pT573	0.95	1.46	0.98	0.000447
p70S6K-pT389	0.89	1.15	0.86	0.006453
Collagen-VI	1.00	1.47	1.44	0.086766

Supplementary Table S1. List of proteins whose phosphorylation or expression significantly changed upon MSP stimulation. Absolute values of significantly changed proteins or phosphoproteins with >1.5 fold change in response to MSP treatment in T47D-RON cells are shown. Data represents mean ± SEM (P values by unpaired, two-sided t-test).

Supplementary Table S2.

	Mock	WT	Mut A	Mut B	Mut C	Mut D	Mut KD
Tumor volume average (mm^3)	1394.00	1421.0	1582.0	1464.0	1285.0	1350.0	1323.0
**Overall Metastasis**	**1/8 (12%)**	**6/14 (43%)**	**8/8 (100%)**	**4/11 (36%)**	**6/13 (46%)**	**1/19 (5.3%)**	**1/17 (5.9%)**
Liver	1/1	2/6	6/8	2/4	3/6	1/1	1/1
Lung	0/1	2/6	6/8	3/4	4/6	1/1	0/1
Brain	0/1	0/6	1/8	1/4	0/6	0/1	0/1
Spleen	1/1	1/6	5/8	1/4	3/6	1/1	1/1
Ovary	1/1	4/6	6/8	3/4	5/6	1/1	1/1
Kidney	0/1	4/6	5/8	3/4	5/6	1/1	0/1
Lymph nodes	0/1	1/6	2/8	0/4	1/6	0/1	0/1

Supplementary Table S2. Gross analysis of metastasis in mice bearing RON mutant tumors. Average tumor volume at the endpoint, number of mice in each group, and tissue tropism of metastasis for different RON mutants are provided.

Supplementary Figure S17

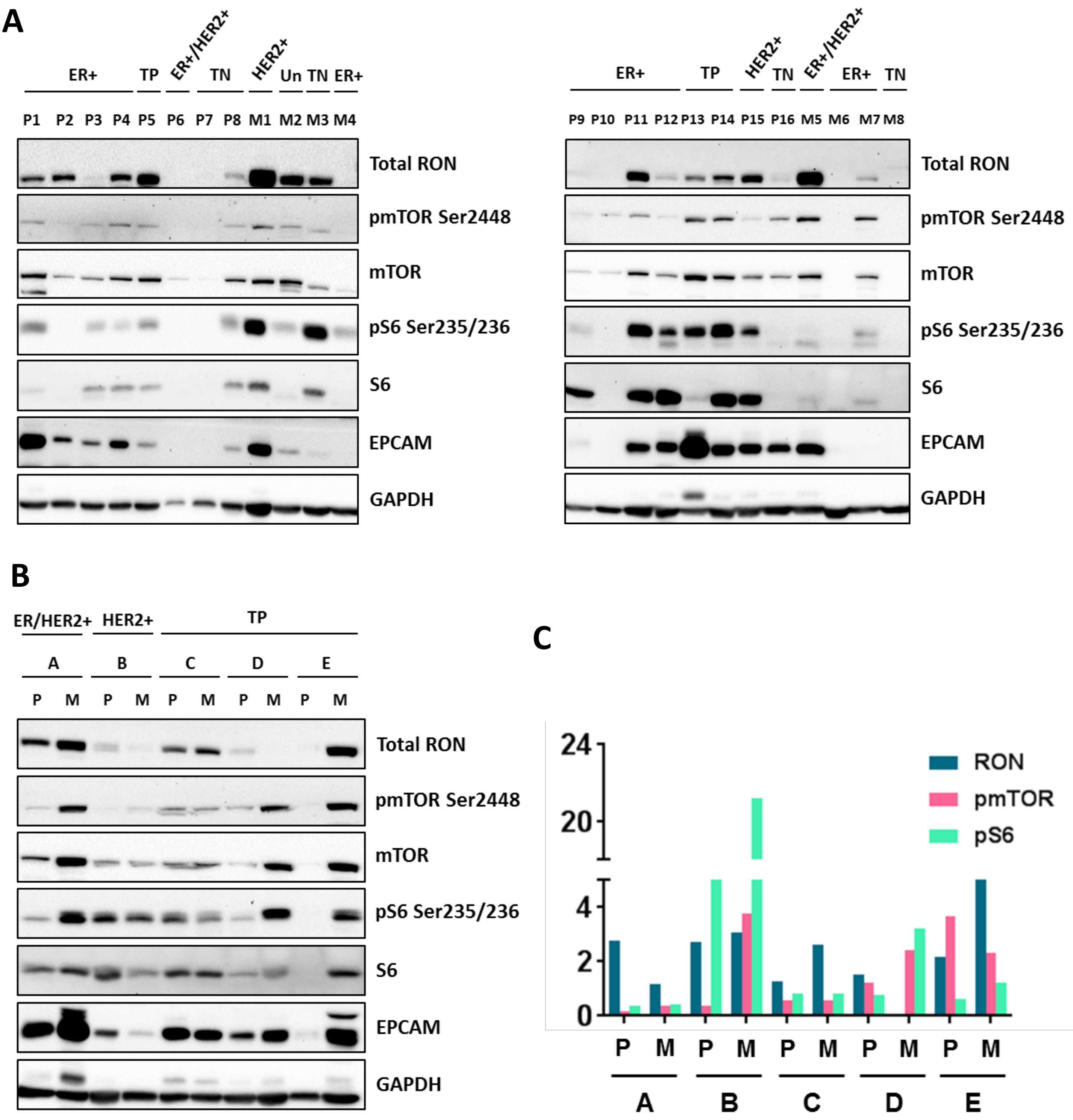

Supplementary Figure S17. Status of RON expression and mTORC1 signaling in human primary and metastatic breast tumors. A) Representative Western blots showing presence of RON and phosphorylated mTORC1 and mTORC1 substrate, rpS6, across different subtypes of unmatched primary and metastatic breast tumors. B) Status of RON and mTORC1 signaling in matched primary and metastatic tumors. EPCAM was used as an epithelial marker to indicate presence of cancer cells, and GAPDH was used as loading control. C) Quantification of RON, pmTOR, and pS6 levels normalized to EPCAM from samples in B. ER, Estrogen receptor, TP: Triple positive (ER/PR/HER2), TN: Triple negative, Un: Unknown.

Supplementary Figure S18

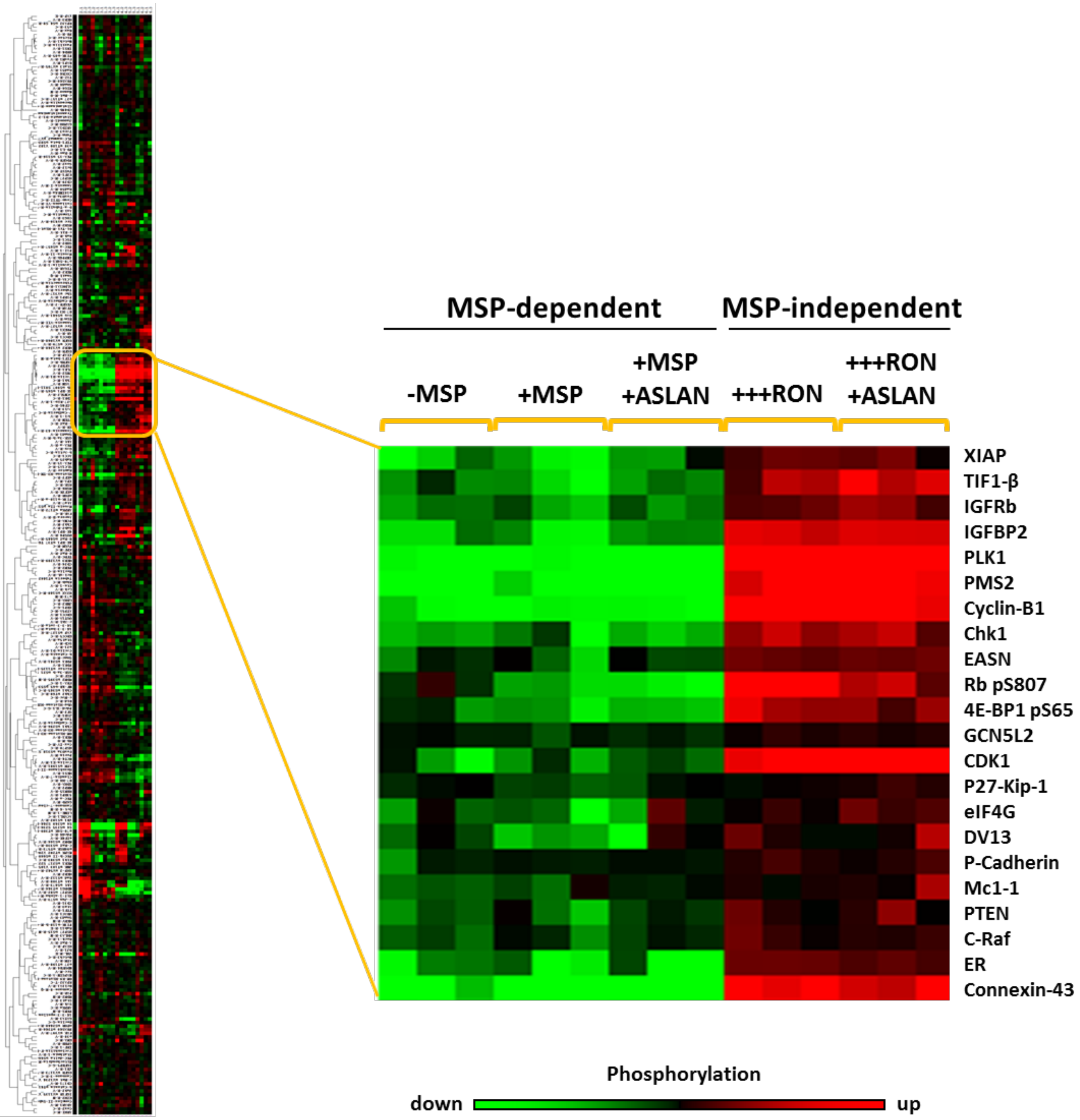

Supplementary Figure S18. Clustered heatmap of 305 proteins is shown for T47D-RON cells in MSP-dependent and –independent conditions and with or without the RON inhibitor ASLAN002. An enlarged cluster of proteins that are particularly changed due to presence or absence of serum are shown on the right.

raw RPPA data						Sample Name		-MSP	-MSP	-MSP	+MSP	+MSP	+MSP	+MSP+ASLAN	+MSP+ASLAN	+MSP+ASLAN	+++RON	+++RON	+++RON	+++RON+ASLA	+++RON+ASLA	+++RON+ASLA
						Sample description		1.1	1.2	1.3	2.1	2.2	2.3	3.1	3.2	3.3	4.1	4.2	4.3	5.1	5.2	5.3
						UUID		40675	40676	40677	40678	40679	40680	40681	40682	40683	40684	40685	40686	40687	40688	40689
						Sample Type		Human Cell L	Human Cell L	Human Cell L	Human Cell L	Human Cell L	Human Cell L	Human Cell L	Human Cell L	Human Cell L	Human Cell L	Human Cell L	Human Cell L	Human Cell L	Human Cell L	Human Cell L
						96-well PCR plate		Plate 8	Plate 8	Plate 8	Plate 8	Plate 8	Plate 8	Plate 8	Plate 8	Plate 8	Plate 8	Plate 8	Plate 8	Plate 8	Plate 8	Plate 8
Filename	Gene Name	Probabilities (	Antibody Nam	Antibody Orig	0 replicated a	96-well definition		C10	C11	C12	D1	D2	D3	D4	D5	D6	E10	E11	E12	F1	F2	F3
14-3-3-beta-R	YWHAB	0.9134788	.4-3-3-beta-R-\	R	1	14-3-3-beta-R	Normalized Li	1.0533008	1.21072818	1.04042771	1.02886327	1.00049031	0.99880076	1.02169833	0.97904127	0.99281768	1.0132657	1.00324934	0.97416481	1.03951048	0.9938228	1.01428088
14-3-3-epsilor	YWHAE	0.9036724	-3-3-epsilon-M	M	2	14-3-3-epsilor	Normalized Li	1.04334421	0.99893463	1.01005985	1.07250375	1	0.96571562	1.03086722	1.00615497	0.99589578	1.0616538	0.9859211	0.97910043	1.05148138	1.00953453	0.9898471
14-3-3-zeta-R-	YWHAZ	0.8909171	l4-3-3-zeta-R-\	R	3	14-3-3-zeta-R-	Normalized Li	1.07267933	1.12868117	1.04634639	1.05597627	0.98902397	1.00451463	1.02177748	0.98309449	1.01130333	1.01211275	0.97113884	0.94858709	0.99724188	0.9757678	0.95754716
4E-BP1-R-V_G	EIF4EBP1	0.9378303	4E-BP1-R-V	R	4	4E-BP1-R-V_G	Normalized Li	1.01643247	0.99836563	1.0036004	1.02992865	0.96947083	0.93022685	0.9466914	1.05177212	1.0631878	1.10110815	1.09084176	1.04721521	1.04403192	1.25220109	1.10446948
4E-BP1_pS65-	EIF4EBP1	0.9419051	E-BP1_pS65-R-	R	5	4E-BP1_pS65-	Normalized Li	0.85223254	0.85848231	0.82679469	0.93819313	0.94735812	0.84486411	0.78188483	0.82311721	0.81294995	1.21644448	1.13546045	1.11589457	1.12469026	1.11807999	1.03939667
4E-BP1_pT37_	EIF4EBP1	0.9397452	3P1_pT37_T46-	R	6	4E-BP1_pT37_	Normalized Li	1.07109265	0.95482111	0.9845774	1.09620583	1.1750435	1.08700633	0.91113578	0.94972476	0.95999066	1.16604593	1.20480088	1.16332455	0.99995102	1.09876423	1.10332479
53BP1-R-V_GI	TP53BP1	0.9365228	53BP1-R-V	R	7	53BP1-R-V_GI	Normalized Li	0.81911594	1.15006693	1.04764404	0.93128602	0.99716519	0.95594761	0.98621282	1.00834402	0.96667966	0.89170723	0.95674176	0.95878927	1.01628636	1.03417479	1.00756344
A-Raf-R-V_GB	ARAF	0.9570826	A-Raf-R-V	R	8	A-Raf-R-V_GB	Normalized Li	1.03076392	0.99269828	1.01449109	1.09713269	1.00103694	0.96405719	0.95580748	0.96174289	0.94683824	1.09167442	0.99407808	0.98188206	1.02120233	0.99795432	0.94623909
ACC_pS79-R-\	ACACA, ACACB	0.9364253	ACC_pS79-R-V	R	9	ACC_pS79-R-\	Normalized Li	1.11145755	0.90503399	0.93601436	1.11719794	0.82773904	0.91449943	0.95928318	1.01583898	0.99752229	0.77887287	0.99473813	0.96874064	0.77275961	0.97309775	1.04089731
ACC1-R-C_GBI	ACACA	0.9452505	ACC1-R-C	R	10	ACC1-R-C_GBI	Normalized Li	0.97108119	0.94811635	0.96785039	1.06224579	0.91020641	0.92881165	0.97614119	0.89817065	0.95316429	1.16459284	1.04282627	1.02646333	1.31158971	1.1682804	0.91554584
ACVRL1-R-C_(	ACVRL1	0.9148413	ACVRL1-R-C	R	11	ACVRL1-R-C_(	Normalized Li	0.97535435	0.99562412	1.01636179	0.95759539	0.96072472	0.99965341	1.04552205	0.97004293	1.00520942	1.0065591	1.01113174	0.98563636	0.95873511	0.97301119	0.99751459
ADAR1-M-V_(	ADAR	0.9162401	ADAR1-M-V	M	12	ADAR1-M-V_(	Normalized Li	1.03316866	1.04705461	1.08487717	0.98349402	0.99866139	0.97712223	0.98119113	0.92892014	0.95625233	1.07648743	1.05980016	1.07792841	1.06978287	1.03865839	0.99670214
Akt-R-V_GBL1	AKT1,2,3	0.8058237	Akt-R-V	R	13	Akt-R-V_GBL1	Normalized Li	1.03060928	1.011474	0.99180856	1.02115478	0.96217619	0.94851451	1.0067282	0.95846293	0.97287154	1.06879986	1.11947183	0.99745241	1.0623876	1.09206626	1.08353107
Akt_pS473-R-'	AKT1,2,3	0.9219374	Akt_pS473-R-V	R	14	Akt_pS473-R-'	Normalized Li	1.02523066	1.17534696	1.09061583	1.8246805	1.7815035	1.4613483	1.07774366	0.89442279	0.86147781	1.22532439	1.17557121	1.16099113	0.86027723	0.72594421	0.7349496
Akt_pT308-R-'	AKT1,2,3	0.928183	Akt_pT308-R-V	R	15	Akt_pT308-R-'	Normalized Li	1.17595845	1.14376071	1.13039772	1.838596	1.4209746	1.42066448	1.14582629	0.87016402	0.88215416	1.0210117	1.0332169	1.03406516	0.70242782	0.65866923	0.66451423
ALDH1A1-R-N	ALDH1A1	0.934665	ALDH1A1-R-N	R	16	ALDH1A1-R-N	Normalized Li	0.7890972	0.96815911	0.951644	0.88341304	0.97168822	0.93852128	1.0285817	1.03110089	1.03152997	1.04484982	1.00666544	0.98088748	1.05999953	0.98943551	0.98847994
AMPKa-R-C_G	PRKAA1	0.9238153	AMPKa-R-C	R	17	AMPKa-R-C_G	Normalized Li	1.02666642	1.02772692	0.99591658	1.09644953	1.01864668	0.96175879	0.99998929	1.02426157	0.98455787	1.05259849	1.01186428	0.97044221	1.06726788	1.00333358	0.96493653
AMPKa_pT17:	PRKAA1	0.9244111	\PKa_pT172-R	R	18	AMPKa_pT17:	Normalized Li	0.61117124	0.71173081	0.90876961	0.51830052	1.00084794	1.16039325	0.53337197	0.99858911	1.10233079	0.99085012	1.158487	1.1992913	0.89560385	1.1793592	1.16264917
Annexin-I-M-\	ANXA1	0.8680894	Annexin-I-M-V	M	19	Annexin-I-M-\	Normalized Li	1	0.94015658	1.01210492	0.97768418	1.03183893	1.14605855	0.98256767	1.02733138	1.08980749	0.98685407	0.95878247	1.03804383	0.93600999	1.00794789	1.0135532
Annexin-VII-N	ANXA7	0.9225792	nnexin-VII-M-'	M	20	Annexin-VII-N	Normalized Li	1.02178948	0.99800292	1.00250275	1.00670771	0.97948944	1.03041634	1.01895014	1.0520723	1.11371363	1.07950259	0.95827432	0.94420516	0.99564515	1.07199233	1.08186929
AR-R-V_GBL1:	AR	0.9039264	AR-R-V	R	21	AR-R-V_GBL1:	Normalized Li	1.00033763	0.94164398	0.90914758	1.0352827	0.9588095	0.94872464	1.01916055	1.01210513	1.02097407	1.02433816	0.9810939	0.95839778	1.03545628	0.99445522	1.00044673
ARHI-M-C_GB	DIRAS3	0.892297	ARHI-M-C	M	22	ARHI-M-C_GB	Normalized Li	1.05536306	1.00463961	1.0081971	0.97027496	0.97879853	0.95871955	1.02441109	0.99557166	1.00331282	1.0219035	0.9777455	0.97377127	1.02382769	1.00019763	0.969306
ARID1A-R-C_(	ARID1A	0.9555633	ARID1A-R-C	R	23	ARID1A-R-C_(	Normalized Li	0.95254859	0.99600567	0.98097191	0.83830568	0.96665488	1.00060661	1.00491085	1.04410787	1.02408848	0.92933299	0.98289731	0.98771032	0.98174394	1.00004405	0.99943804
Atg3-R-V_GBL	ATG3	0.880206	Atg3-R-V	R	24	Atg3-R-V_GBL	Normalized Li	0.98266793	0.93244798	1.03172752	1.00186794	1.01879671	1.02892072	1.01851044	0.99172679	1.02316875	1.01573519	0.98099933	0.98832201	0.98319613	1.01052618	0.99860396
Atg7-R-V_GBL	ATG7	0.9437925	Atg7-R-V	R	25	Atg7-R-V_GBL	Normalized Li	0.95141215	0.99880438	1.00017969	0.91029214	0.97321133	0.96813029	0.91364864	0.95929714	0.99858627	0.98485836	1.02633508	1.00711581	0.97494137	1.03611722	1.0222314
ATM-R-V_GBL	ATM	0.9430356	ATM-R-V	R	26	ATM-R-V_GBL	Normalized Li	1.06950273	0.97736365	0.96191156	1.06343914	1.00411888	0.99078444	1.05076168	1.03904897	1.03605986	1.02170022	0.97059839	0.94924414	1.06317969	0.92934415	0.96663472
ATM_pS1981-	ATM	0.8634538	TM_pS1981-R-	R	27	ATM_pS1981-	Normalized Li	1.01862826	1.04890157	1.00594636	0.98181852	0.99850965	1.0166954	1.03391761	1.00618516	1.00642943	0.94082568	0.97277285	0.98056637	0.9335014	0.93248519	0.96430044
ATP5A-M-C_G	ATP5A	0.9138529	ATP5A-M-C	M	28	ATP5A-M-C_G	Normalized Li	1.02629806	1.59975229	1.00979452	1.03413939	1.01107507	0.98519141	0.98693816	0.98119443	1.01314497	1.02719923	0.97671308	0.93820393	1.0150428	1.01530989	0.99151168
Aurora-B-R-V_	AIM1	0.8910837	Aurora-B-R-V	R	29	Aurora-B-R-V_	Normalized Li	0.94751242	1.03793806	1.00677179	0.92854337	1.02370692	1.01846807	0.95731591	1.00041687	0.98846351	0.99387635	1.10989622	1.14404472	0.98625812	1.13804316	1.13333879
Axl-R-V_GBL1	AXL	0.8784218	Axl-R-V	R	30	Axl-R-V_GBL1	Normalized Li	0.86125875	0.86888924	0.95523006	0.96691229	1.00588855	1.02189776	1.00555658	1.09572079	1.07050326	0.83469365	0.9394041	0.99992834	0.97706447	1.11245027	1.05808398
b-Actin-R-C_G	ACTB	0.9111305	b-Actin-R-C	R	31	b-Actin-R-C_G	Normalized Li	0.87962624	0.86528696	0.92789501	0.87887943	0.83875772	0.90521914	1.00919159	0.78874471	0.86491801	1.17335783	1.04125309	1.04027444	1.12900062	1.06703337	1.0479748
b-Catenin-R-V	CTNNB1	0.93975	b-Catenin-R-V	R	32	b-Catenin-R-V	Normalized Li	0.97874141	1.28468022	0.9825864	0.99125885	1.15624836	1.03690505	0.94577313	1.20624865	1.11208024	0.87635981	0.93080771	0.90642941	0.93785951	0.95410296	0.9808576
b-Catenin_pT4	CTNNB1	0.9345286	enin_pT41_S4!	R	33	b-Catenin_pT4	Normalized Li	1.00311235	0.96645904	0.990695	1.01076321	1.00647681	1.01702619	1.04592424	0.994042	1.0051726	0.98857391	0.96669351	1.0070157	1.02622926	1.02429549	0.9866034
B-Raf-R-V_GB	BRAF	0.94569	B-Raf-R-V	R	34	B-Raf-R-V_GB	Normalized Li	1.01924034	1.02644207	1.00318724	1.03283805	1.00393281	0.98155497	0.99704722	1.00769795	0.97850623	1.02368864	1.00061917	1.00385508	1.04048995	1.032147	1.02297265
B-Raf_pS445-I	BRAF	0.9494865	-Raf_pS445-R-'	R	35	B-Raf_pS445-I	Normalized Li	0.94477625	0.97603246	0.94590796	1.01880142	0.99404294	1.01289264	0.93309284	0.98062842	0.95531327	1.11725811	1.09607461	1.04413666	1.00345777	0.99036348	1.03672294
B7-H3-R-C_GE	CD276	0.8338371	B7-H3-R-C	R	36	B7-H3-R-C_GE	Normalized Li	0.92020093	0.9151923	0.99670346	0.90191102	0.98517355	0.98038789	0.98181988	0.9581563	1.03276418	0.96042229	0.99806105	0.96613479	0.89339696	1.03269112	0.97354869
B7-H4-R-C_GE	VTCN1	0.82379	B7-H4-R-C	R	37	B7-H4-R-C_GE	Normalized Li	1.10708969	0.99286691	1.07360335	1.07356058	1.05920739	1.10879611	1.17339236	1.05898514	1.07884849	0.8263558	0.92329642	0.93424384	0.83464433	0.91191054	0.93678378
Bad_pS112-R-	BAD	0.9339524	3ad_pS112-R-V	R	38	Bad_pS112-R-	Normalized Li	1.00208706	1.01922848	1.02446016	1.34836461	1.21770645	1.21701649	0.99665893	1.0163811	0.99546476	1.09892021	1.06402076	1.01468698	0.918316	0.92054043	0.92211863
Bak-R-C_GBL1	BAK1	0.9239455	Bak-R-C	R	39	Bak-R-C_GBL1	Normalized Li	0.97288826	1.03093262	1.03819237	1.03375664	1.00725388	1.02807633	0.99490284	1.0147258	0.99860828	1.01622474	1.04089584	1.03545824	1.04874663	1.04284076	1.03990909
BAP1-M-V_GE	BAP1	0.9153061	BAP1-M-V	M	40	BAP1-M-V_GE	Normalized Li	0.99404466	1.01809717	1.00695903	0.99999615	0.99438275	1.00531001	0.95697447	1.01013279	1.02372287	0.968024	1.01381389	0.99892854	1.00254252	1.03068662	1.02797652
Bax-R-V_GBL1	BAX	0.9419238	Bax-R-V	R	41	Bax-R-V_GBL1	Normalized Li	0.95207006	1.03797962	1.00734141	0.97021441	1.01201883	1	0.97540307	1.01262847	1.04961363	0.90318387	0.97412722	0.9652	0.9753299	0.99679379	0.95800797
Bcl-xL-R-V_GB	BCL2L1	0.9015682	Bcl-xL-R-V	R	42	Bcl-xL-R-V_GB	Normalized Li	1.02313859	1.16242484	0.99240559	1.04193035	1.00174009	0.93761531	0.98716503	1.05999503	1.07341312	1.04393541	0.95426278	0.94500413	1.04239387	0.99865329	0.93512255
Bcl2-M-V_GBI	BCL2	0.8322661	Bcl2-M-V	M	43	Bcl2-M-V_GBI	Normalized Li	0.93936118	0.9626835	1.05325729	0.92294195	1.0368743	1.08378904	0.95616344	1.01639335	1.03898449	0.86538644	0.98420592	0.954393	0.94984251	0.98013491	0.97572571
Bcl2A1-R-V_G	BCL2A1	0.8489524	Bcl2A1-R-V	R	44	Bcl2A1-R-V_G	Normalized Li	1.03597836	0.99918045	1.03254253	1.07704125	0.9721333	0.96166524	1.0659232	1	0.99741918	1.01788853	1.00233419	0.98359487	1.04666706	0.98118023	0.96166659
Beclin-G-C_GE	BECN1	0.8868744	Beclin-G-C	G	45	Beclin-G-C_GE	Normalized Li	1.02974955	0.98822566	0.99997955	1.0054123	0.95632638	0.97080988	1.03934235	1.02012453	1.01181643	1.04218975	0.99125091	0.98359784	1.054252	0.97835201	0.94994506
Bid-R-C_GBL1	BID	0.9440966	Bid-R-C	R	46	Bid-R-C_GBL1	Normalized Li	0.99493595	1.08734894	0.96870827	1.05501123	0.98144136	0.975167	1.02826516	1.03370621	0.9938696	0.97250898	1.0025171	0.95852179	0.99058352	0.99505765	1.01671837
Bim-R-V_GBL:	BCL2L11	0.9265852	Bim-R-V	R	47	Bim-R-V_GBL:	Normalized Li	1.00395904	1.00655438	0.99672115	0.89839407	0.96848637	0.96866648	1	1.07452124	1.06602488	0.86064575	0.86995396	0.87247632	0.97986116	0.9874481	1.01082389
BRD4-R-V_GB	BRD4	0.9494312	BRD4-R-V	R	48	BRD4-R-V_GB	Normalized Li	0.94629978	1.02480019	0.94282193	0.87933931	1.1106853	0.95695009	0.86450295	1.19593482	1.03168338	0.87886658	0.98425081	0.96834947	0.95467997	1.06061252	0.97236535
c-Abl-R-V_GBI	ABL	0.9270364	c-Abl-R-V	R	49	c-Abl-R-V_GBI	Normalized Li	0.99981117	1.10409156	1.01497578	1.02493374	1.01720274	1.0000397	1.0236648	0.96545576	0.9711658	0.9921352	0.97620873	0.98845911	1.00569355	0.98991965	1.02580599
c-Jun_pS73-R-	JUN	0.9245723	:-Jun_pS73-R-\	R	50	c-Jun_pS73-R-	Normalized Li	0.97807459	0.97919637	0.98928671	1.05609899	1.14619062	1.09786123	0.97138788	1.09072659	1.02596963	0.93014547	1.02832742	1.01030689	0.88774073	0.95884419	0.99593957
c-Kit-R-V_GBL	KIT	0.8710827	c-Kit-R-V	R	51	c-Kit-R-V_GBL	Normalized Li	1.00263315	0.96751711	1.00100702	1.05297906	1.00928112	1.02674913	1.04565231	1.01907834	1.03495748	1.00793554	1.03957558	1.0145359	1.08202467	1.02469495	1.05305244
c-Met-M-Q_G	MET	0.8819528	c-Met-M-Q	M	52	c-Met-M-Q_G	Normalized Li	0.99955267	1.00205607	1.00554097	0.93720921	0.97484507	1.0376245	1.03730162	0.98742179	0.99577898	0.96057277	1.00785331	1.00308722	0.97103534	0.98183194	1.00768389
c-Met_pY1234	MET	0.8514864	_pY1234_Y123	R	53	c-Met_pY1234	Normalized Li	1.00213167	0.9822767	0.99955372	1.07847155	1.00473712	1.05276922	1.03841441	1.00551494	0.98872818	1.04333359	1.01750007	1.01884469	1.05292258	1.07704319	1.081877
c-Myc-R-C_GE	MYC	0.9165627	c-Myc-R-C	R	54	c-Myc-R-C_GE	Normalized Li	1.03872805	1.14265314	0.97707754	1.07280233	1.01717868	0.99880662	1.09797326	1.02113501	0.98015938	0.97133285	1.02576217	0.96895302	0.98506895	0.92749642	0.99079411
c-Raf-R-C_GBI	RAF1	0.952963	c-Raf-R-C	R	55	c-Raf-R-C_GBI	Normalized Li	0.98543436	1.0111437	0.95905589	0.93475725	0.96498282	0.92279688	0.89357619	1.00305078	0.98465073	1.13175012	1.0728989	1.02961448	1.07591893	1.05777617	1.05008779
C-Raf_pS338-I	RAF1	0.8901179	-Raf_pS338-R-'	R	56	C-Raf_pS338-I	Normalized Li	1.0586055	0.91524171	1.01372825	1.37340414	1.20357972	1.22826117	0.93107679	0.8679813	0.87850697	1.23644347	1.18197107	1.15291541	1.02141168	0.91813728	0.91411089
Caspase-3-R-C	CASP3	0.9345255	Caspase-3-R-C	R	57	Caspase-3-R-C	Normalized Li	1.01039732	0.97615617	0.98515375	1.0489816	1.04169005	1.02416393	1.03974081	1.00981963	1.0090093	1.05097901	1.00570009	0.99729722	1.06413958	1.06029021	1.03847369
Caspase-7-cle:	CASP7	0.9157476	›ase-7-cleaved	R	58	Caspase-7-cle:	Normalized Li	0.95942391	1.03637975	0.99659728	1.01440731	1.00969409	1.02916028	1.02143183	1.02356846	1.00590958	0.92617464	1.02317976	0.99523489	0.98912302	0.96134456	1.01360681
Caspase-8-M-I	CASP8	0.9346964	Caspase-8-M-C	M	59	Caspase-8-M-I	Normalized Li	1.06376372	0.98837362	0.99750973	1.11518056	1.00070338	1.00558337	1.10188568	1.02263938	1.01625609	1.02882376	0.9566704	0.97368522	1.05249592	1.00301311	0.97808585
Caveolin-1-R-\	CAV1	0.9290555	Caveolin-1-R-V	R	60	Caveolin-1-R-\	Normalized Li	0.84648348	0.91362102	0.96214563	0.96267623	0.97676944	1.16942209	0.91311533	1.2591946	1.21761337	0.90659637	1.0780558	1.12616443	0.90634243	1.20360174	1.16351753
CD171-M-V_G	L1CAM	0.904802	CD171-M-V	M	61	CD171-M-V_G	Normalized Li	1.03883375	0.92311067	0.97498811	1.07709029	0.95105964	0.97833703	1.29289571	0.98609908	1.01170696	0.99875979	0.95478378	0.96072284	1.12672967	0.96105048	0.9609106
CD26-R-V_GBI	CD26	0.9476447	CD26-R-V	R	62	CD26-R-V_GBI	Normalized Li	0.98892209	1.06868845	0.99444317	1.00816114	1.01145738	0.97678189	0.96491731	0.99233399	1.0007103	0.99559606	1.02608043	0.97967444	1.01070739	1.00902942	1.01826196
CD29-M-V_GE	ITGB1	0.8582124	CD29-M-V	M	63	CD29-M-V_GE	Normalized Li	0.92608954	0.90610821	1.13106695	0.91134715	1.08676365	1.12434721	0.97812082	1.02666669	1.11089163	0.90117247	0.83565964	1.0957939	0.89373613	1.06611055	1.07224777
CD31-M-V_GE	PECAM1	0.8902243	CD31-M-V	M	64	CD31-M-V_GE	Normalized Li	0.97352017	0.94594665	1.0538404	1.05941263	1.0303125	1.02536854	1.03458774	0.99919843	0.99658743	0.99500966	0.97163678	0.97631002	1.00311787	0.99755738	0.97354086
CD49b-M-V_G	ITGA2	0.8678006	CD49b-M-V	M	65	CD49b-M-V_G	Normalized Li	0.97677339	0.99944088	0.98940712	0.92339816	0.98388674	1.01045264	1.02501626	1.028496	1.04278469	0.88622611	1.55476852	0.94125985	0.9069319	0.99007056	0.97592409
CDK1-R-C_GBI	CDK1	0.9439401	CDK1-R-C	R	66	CDK1-R-C_GBI	Normalized Li	0.97378609	0.88232706	0.93080186	0.9947021	0.8751997	0.80144336	0.85791785	0.9580281	0.91639572	1.25759959	1.64222956	1.63725376	1.79239829	1.65235877	1.5721853
Chk1-M-C_GB	CHEK1	0.9010838	Chk1-M-C	M	67	Chk1-M-C_GB	Normalized Li	0.99435579	0.93574468	0.89239147	0.8812185	0.90559418	0.89332374	0.79763104	0.86356992	0.89229901	1.19595389	1.26470558	1.19062126	1.13092119	1.22501019	1.25965586
Chk1_pS296-F	CHEK1	0.9445394	:hk1_pS296-R-'	R	68	Chk1_pS296-F	Normalized Li	1.0666093	1.06012515	1.04929663	1.07029804	0.97242628	0.96997855	1.01214761	1.00909763	1.00812796	1.0191533	1.02313736	1.00051707	0.97232993	0.93186045	0.96642151
Chk1_pS345-F	CHEK1	0.9540543	:hk1_pS345-R-(	R	69	Chk1_pS345-F	Normalized Li	1.12822542	1.18976172	1.10936632	1.14708988	1.11469711	1.0368776	1.02971197	1.14123255	1.11167607	0.94831354	0.88778508	0.92977091	0.96446857	0.93625576	0.92827138
Chk2-M-V_GB	CHEK2	0.9066806	Chk2-M-V	M	70	Chk2-M-V_GB	Normalized Li	0.98902303	1.08029749	1.01121533	0.99648576	1.01200951	0.99935927	0.95722698	0.96134983	1.00338486	1.02946369	1.06046618	1.05732181	1.01247521	1.05750181	1.09187588
Chk2_pT68-R-	CHEK2	0.91165	Chk2_pT68-R-C	R	71	Chk2_pT68-R-	Normalized Li	1.03831018	1.10472267	1.07828006	1.05456112	1.0086758	1.00633733	1.03113307	1.06579926	1.03629443	0.98468275	0.98683461	0.95718665	0.94220635	0.9222047	0.94416114
Claudin-7-R-V	CLDN7	0.9084938	Claudin-7-R-V	R	72	Claudin-7-R-V	Normalized Li	1.11741177	1.08438039	1.12134193	1.16278626	1.11251415	1.12729234	1.15440676	1.1486789	1.24320442	0.8268246	0.91235485	0.92913541	0.83698068	0.89726918	0.91538876
COG3-R-V_GB	COG3	0.9456965	COG3-R-V	R	73	COG3-R-V_GB	Normalized Li	1.06792269	1.00342205	0.99586148	1.02405652	1.01583458	0.97151262	0.92072915	1.04457315	1.083349	1.04964916	1.00643803	0.96587326	0.98572208	1.02141481	0.9876728
Collagen-VI-R-	COL6A1	0.8596365	Collagen-VI-R-\	R	74	Collagen-VI-R-	Normalized Li	1.06701796	0.93020894	1.0028389	1.0792913	1.56802366	1.77336828	1.05133759	1.54903794	1.70958527	0.97894783	0.95064011	0.93558515	1.0050427	1.60745653	1.46262814
Complex-II-Su	SDHB	0.8941403	plex-II-Subunit	M	75	Complex-II-Subunit-M-V_GBL1120985	Normalized Li	0.9667598	0.98401801	1.01429597	0.99162025	0.91060834	0.93980252	0.97725178	0.96841835	1.00066115	0.99295123	1.08258968	1.06328187	1.0799916	1.07605113	1.01245613
Connexin-43-I	CNST43	0.8213715	onnexin-43-R-(	R	76	Connexin-43-I	Normalized Li	0.6345642	0.61937399	0.71511839	0.71969373	0.74058592	0.81791672	0.7300439	0.74810222	0.75652619	1.2338388	1.20557774	1.26581006	1.35428929	1.18315115	1.16169243
Coup-TFII-R-C_	NR2F2	0.953506	Coup-TFII-R-C	R	77	Coup-TFII-R-C_	Normalized Li	0.98096713	0.94899741	1.00041694	1.02454966	1.14295545	1.13327239	1.0136176	1.10570245	1.10986658	0.88405228	0.98611068	0.96247328	0.95411297	1.00879219	1.02595815
Cox-IV-M-C_G	COX4I1	0.8858602	Cox-IV-M-C	M	78	Cox-IV-M-C_G	Normalized Li	1.08827	1.00475047	1.01515851	1.01207922	0.95736581	0.99692699	1.04774396	0.96792728	1.02872892	0.94729782	0.96150701	0.95461693	1.01799552	0.97769136	0.99107131
Cox2-R-C_GBL	PTGS2	0.9291299	Cox2-R-C	R	79	Cox2-R-C_GBL	Normalized Li	1.0265586	0.99884938	1.00280434	0.95464487	0.99003854	0.95980749	1.04324672	0.97073991	0.98077032	1.0430845	0.9756817	0.97546291	1.02245262	0.98851532	0.98461999
CXCR4-R-C_GI	CXCR4	0.8986678	CXCR4-R-C	R	80	CXCR4-R-C_GI	Normalized Li	0.93684916	1.02247622	1.02696859	0.91750128	0.99328186	1.01661734	0.96639764	0.97637417	0.98610513	0.8982196	1.00483239	1.01040334	0.93823345	0.99796944	0.99774926
Cyclin-B1-R-V_	CCNB1	0.9215507	Cyclin-B1-R-V	R	81	Cyclin-B1-R-V_	Normalized Li	0.8093228	0.77988674	0.8054458	0.84206501	0.80115994	0.78643825	0.80452194	0.80306724	0.78896138	1.28401579	1.44316165	1.39190593	1.25571805	1.54223517	1.53992615
Cyclin-D1-R-V_	CCND1	0.9306014	Cyclin-D1-R-V	R	82	Cyclin-D1-R-V_	Normalized Li	1.01391836	1.13258042	0.97790216	0.94141403	1.02696572	0.9699936	1.0060735	1.05111133	1.04151482	0.98800617	0.95917196	0.97250971	0.92636596	0.94196662	0.9381844
Cyclin-E1-M-V	CCNE1	0.8852886	Cyclin-E1-M-V	M	83	Cyclin-E1-M-V	Normalized Li	1.06356825	1.05421507	1.02550268	1.00875107	0.99686088	1.04457278	1.0312138	1.01052076	1.02782476	0.98168451	0.96855404	0.96172746	0.96704695	0.93911923	0.97022154
Cyclophilin-F-I	PPIF	0.9311531	yclophilin-F-M-	M	84	Cyclophilin-F-I	Normalized Li	1.00092803	1.00644018	0.99344164	1.02387075	0.94513286	0.94060191	0.96721478	1.00400644	1.01045368	1.02755662	0.96354427	0.95358535	1.02130646	1.00780426	0.97299283
D-a-Tubulin-R	TUBA1A	0.9229401	)-a-Tubulin-R-\	R	85	D-a-Tubulin-R	Normalized Li	0.97022358	0.93750138	0.99691465	0.99015716	1.01931325	1.04452146	1.05576364	1.02077527	1.0286117	0.93110329	0.95069822	0.97162951	0.94927173	1.00823862	1.03910481
Di-Tri-M-Hist-	HIST1H3A	0.9156967	'ri-M-Hist-H3-I\	M	86	Di-Tri-M-Hist-	Normalized Li	1.05089604	0.95572832	1.07288847	0.96791927	1.0335948	1.02412555	0.97333413	0.93035694	1.07856083	1.05754953	1.03338593	1.1470439	1.0218275	1.04262747	1.11069478
DJ1-R-V_GBL1	PARK7	0.9379048	DJ1-R-V	R	87	DJ1-R-V_GBL1	Normalized Li	1.04572866	0.99021356	1.00176796	1.07405241	0.97918208	0.98377021	1.05682077	0.95666729	0.98577931	1.06267172	0.98585434	0.97224365	1.08605162	1.04064959	1.0292184
DM-Histone-H	HISTH3	0.9089594	I-Histone-H3-F	R	88	DM-Histone-H	Normalized Li	1.05267624	1.04043421	1.05356639	1.05949059	0.96460348	0.98808296	1.09244519	0.97297278	1.00749704	0.93163255	0.96100807	0.97323034	0.99426044	0.93243171	0.94845035
DM-K9-Histon	H3K9ME2	0.9330006	K9-Histone-H3	R	89	DM-K9-Histon	Normalized Li	1.06637441	1.00341208	1.05144385	1.15283784	0.97823266	0.99964981	1.14945635	0.99207451	0.9826585	1.01639262	0.95026417	1.00400416	1.08581931	0.98449075	0.97772497
DUSP4-R-V_G	DUSP4	0.9250428	DUSP4-R-V	R	90	DUSP4-R-V_G	Normalized Li	0.68181535	0.96464155	1.04863192	0.66684355	0.9706202	0.98155944	0.81114978	0.74361734	0.54997785	2.36400311	2.83708417	2.81327184	0.76292122	1.24930862	1.32933324
Dvl3-R-V_GBL	DVL3	0.951818	Dvl3-R-V	R	91	Dvl3-R-V_GBL	Normalized Li	0.85070097	0.81273708	0.77040296	0.88578556	0.9790945	0.91604495	0.83345883	1.02118796	0.97255138	1.07596808	1.00379106	0.96664871	1.15589428	0.96667648	0.99255804
E-Cadherin-R-	CDH1	0.8932715	E-Cadherin-R-\	R	92	E-Cadherin-R-	Normalized Li	1.27287735	1.15233294	1.16949485	1.11560202	1.1081823	1.01572312	1.05664519	1.15085877	1.13400004	1.14323169	0.9236818	0.89015414	1.00608874	0.91210872	0.79624886
E2F1-M-V_GB	E2F1	0.8671758	E2F1-M-V	M	93	E2F1-M-V_GB	Normalized Li	0.93996326	0.88194837	1.08839647	0.94365639	0.96888734	1.11622681	1.00431424	1.03359833	1.07985001	0.88748132	0.97575568	1.00322663	0.89841221	1.0473787	1.05803237
eEF2-R-C_GBL	EEF2	0.9565503	eEF2-R-C	R	94	eEF2-R-C_GBL	Normalized Li	0.96393546	1.36151098	0.91116848	0.88506119	0.92867766	0.89549006	0.8294003	0.92450779	0.91407334	1.13346594	1.05882422	1.01427033	1.06533946	1.03992615	1.05828442
eEF2K-R-V_GE	EEF2K	0.9563823	eEF2K-R-V	R	95	eEF2K-R-V_GE	Normalized Li	0.9451667	1.03288335	0.93118273	0.94196328	0.99410602	0.92582016	0.88520052	1.0007364	0.9805668	1.01446782	1.0600356	1.03182071	1.00105415	1.1428879	1.11526326
EGFR-R-V_GBI	EGFR	0.9317842	EGFR-R-V	R	96	EGFR-R-V_GBI	Normalized Li	0.96037584	0.97352148	0.9953589	0.94138253	0.9955271	1.02627961	1.03349334	0.97887455	1.00296684	0.93205939	0.9670738	0.98666961	0.98744758	1.02875547	0.99043384
EGFR_pY1068	EGFR	0.8879428	GFR_pY1068-R-	R	97	EGFR_pY1068	Normalized Li	0.99243082	0.88304576	0.89684549	0.99688413	0.98081454	1.04425109	0.98515491	0.99269147	0.99450487	0.98507162	1.00037307	1.00026747	0.95897989	0.93751325	1.01623768
EGFR_pY1173	EGFR	0.8743686	GFR_pY1173-R-	R	98	EGFR_pY1173	Normalized Li	0.9639722	0.98608886	0.9482144	0.99689127	0.98632549	1.02088128	1.03446527	0.99638353	0.99670485	1.05826945	1.01948851	1.00716937	1.06722571	1.03861247	1.05739961
eIF4E-R-V_GB	EIF4E	0.8991871	eIF4E-R-V	R	99	eIF4E-R-V_GB	Normalized Li	1.09308146	0.97199532	0.99062534	1.21254446	1.07971212	1.04025288	0.87736347	0.91452993	0.9008453	1.17024926	1.05659254	1.00744371	0.90778657	0.89267667	0.90141639
eIF4G-R-C_GB	EIF4G1	0.948297	eIF4G-R-C	R	100	eIF4G-R-C_GB	Normalized Li	0.92402257	0.9398873	0.86616654	0.87733441	1.02626648	0.92630035	0.78514294	1.10533161	0.98833997	1.02087069	1.06804339	1.02418166	1.09217049	1.12172842	1.06901548
Elk1_pS383-R-	ELK1	0.8723763	Elk1_pS383-R-(	R	101	Elk1_pS383-R-	Normalized Li	0.87989768	0.98151463	1.02018413	1.28518333	1.25046966	1.28675444	0.81400969	0.95115204	0.95324777	1.14719865	1.23184045	1.21755905	1.03950216	1.0342564	1.04863369
EMA-M-C_GBI	EMA	0.8729423	EMA-M-C	M	102	EMA-M-C_GBI	Normalized Li	1.06308744	0.96706075	0.98180792	1.06968162	0.97273189	0.88543375	1.0727434	0.93640092	0.97564881	1.82722481	0.98823946	0.99242068	1.91554761	0.9397544	0.90111788
ER-R-V_GBL11	ESR1	0.9115945	ER-R-V	R	103	ER-R-V_GBL11	Normalized Li	0.80726373	0.94702701	0.96495929	0.81740009	0.91618693	0.92278124	0.8271483	0.80939557	0.80198787	1.13403461	1.13519611	1.13012848	1.09652734	1.10384955	1.1233622
ERCC1-M-V_G	ERCC1	0.9112591	ERCC1-M-V	M	104	ERCC1-M-V_G	Normalized Li	1.07091056	1.16096146	0.99919443	0.98477693	0.99885607	0.98326916	1.01563393	0.99733009	0.98903963	1.03189432	1.01029119	0.99467868	0.98758829	1.0178389	1.02559614
ERCC5-R-C_GE	ERCC5	0.936187	ERCC5-R-C	R	105	ERCC5-R-C_GE	Normalized Li	1.01276661	1.03543448	0.96780421	0.95249603	1.02900214	0.97373387	0.95429907	1.06049529	1.0204819	0.96819305	0.98532319	0.97410451	0.90744506	0.97174435	0.93059046
Ets-1-R-V_GBI	ETS1	0.8882157	Ets-1-R-V	R	106	Ets-1-R-V_GBI	Normalized Li	1.02842747	1.1781984	1.01551828	0.91168065	1.01891333	0.99754106	1.03599866	0.9904753	0.98907543	0.94653822	0.99930386	0.98362518	0.96864431	0.99019165	1.01049679
EZH2-R-N_GBI	EZH2	0.9537763	EZH2-R-N	R	107	EZH2-R-N_GBI	Normalized Li	1.028683	0.99422291	0.98832568	1.04239658	0.98899378	0.94225556	0.9902533	0.98653018	0.96436968	1.10919944	1.05181466	1.02403132	1.05673676	1.05966534	0.99856636
FAK-R-C_GBL1	PTK2	0.9023965	FAK-R-C	R	108	FAK-R-C_GBL1	Normalized Li	0.93651563	1.10506212	0.98958854	0.86214113	0.98441637	0.96568115	0.92959265	1.00180206	1.00557094	1.01838156	1.09351972	1.07529591	1.04432614	1.10527979	1.1331379
FAK_pY397-R-	PTK2	0.903995	FAK_pY397-R-\	R	109	FAK_pY397-R-	Normalized Li	1.05912478	1.01167091	1.01738021	1.10461589	1.09334001	1.06826115	1.06952701	0.95754691	0.96819056	0.99834929	0.95219788	0.96372644	0.96539061	0.86681601	0.86332911
FASN-R-V_GBI	FASN	0.9508446	FASN-R-V	R	110	FASN-R-V_GBI	Normalized Li	0.91334342	1.00231348	0.99836194	0.88275404	0.97966291	0.95727735	0.81969862	0.9346022	0.93753822	1.11009327	1.09577997	1.08222774	1.10740762	1.10127854	1.09142378
Fibronectin-R-	FN1	0.8536611	ibronectin-R-\	R	111	Fibronectin-R-	Normalized Li	0.9036515	0.94124282	0.99462521	0.94519386	0.98721051	1.02043828	0.95814994	1.01090319	1.03111841	1.0004658	1.01146068	1.01960228	1.06314037	1.00499643	1.00791454
FoxM1-R-V_G	FOXM1	0.9273971	FoxM1-R-V	R	112	FoxM1-R-V_G	Normalized Li	0.95260608	1.00454587	1.00269579	0.94462386	0.98470331	1.02186956	0.87002457	1.01735878	0.98584527	0.89118247	1.03873225	1.01401873	0.95194522	1.02553936	1.11252221
FoxO3a-R-C_(	FOXO3	0.8394615	FoxO3a-R-C	R	113	FoxO3a-R-C_(	Normalized Li	0.96961623	0.96379102	0.98113799	1.01809516	1.00519674	1.01364712	1.01818638	1.04801778	1.05536924	0.97866739	0.99720616	0.97896843	0.97808061	0.99290535	0.97334042
FoxO3a_pS31!	FOXO3	0.9058771	3a_pS318_S32	R	114	FoxO3a_pS31!	Normalized Li	1.06381973	1.05235123	1.01773017	1.04001947	1.01474374	1.05337169	0.98406092	1.00366603	0.99915626	1.02836956	0.98771316	0.9746173	1.0037626	0.99953563	1.00194695
FRA-1-R-C_GB	FRA1	0.9257219	FRA-1-R-C	R	115	FRA-1-R-C_GB	Normalized Li	1.07036132	1.09721227	1.02571948	1.04202427	1.01428975	0.98194389	1.02892427	1.0329013	1.02962655	0.99992041	0.9495728	0.94705416	0.99196839	0.93728194	0.95361368
G6PD-M-V_GI	G6PD	0.8503263	G6PD-M-V	M	116	G6PD-M-V_GI	Normalized Li	0.99578418	1.2056354	1.03658399	0.97077995	0.99546913	1.09576763	0.97561908	1.00417696	1.01202143	0.97922392	1.00726179	1.11587498	0.92195695	0.9694802	1.06594028
Gab2-R-V_GBI	GAB2	0.9541478	Gab2-R-V	R	117	Gab2-R-V_GBI	Normalized Li	0.99149063	1.0310173	1.00488205	1.01509175	1.01169382	0.92394109	0.90049584	1.09691438	0.99034071	1.05460239	1.29278987	1.27111052	1.01609895	1.36062495	1.26107399
GAPDH-M-C_(	GAPDH	0.9328298	GAPDH-M-C	M	118	GAPDH-M-C_(	Normalized Li	1.02105617	1.00264981	1.00356802	0.52945192	0.99609311	0.93068587	0.99858838	0.98324267	1.01259317	0.7365665	0.96193995	0.97405556	0.7206345	1.04005733	0.92821875
GATA3-M-V_(	GATA3	0.8619674	GATA3-M-V	M	119	GATA3-M-V_(	Normalized Li	1.05348896	1.10731793	1.09975612	1.03336734	1.09800373	1.06458852	1.02912921	1.05642291	1.00182107	1.16476028	0.8488421	0.82677048	1.20279713	0.82270641	0.77289651
GCN5L2-R-V_(	KAT2A	0.9285965	GCN5L2-R-V	R	120	GCN5L2-R-V_(	Normalized Li	0.94952989	0.99539062	0.98472905	1.00965389	1.01932195	0.99418163	1.00668374	1.00372423	0.98026596	1.03763364	1.0729476	1.05176055	1.06120373	1.06614812	1.04888919
Glutamate-D1	GLUD	0.9073117	utamate-D1-2-I	R	121	Glutamate-D1	Normalized Li	0.96614991	1.03422739	0.98986017	0.85545631	0.98823495	0.99234323	0.98583573	0.97218177	1.01554038	0.82832589	0.99521168	0.97690184	0.97784191	0.98912371	0.97291037
Glutaminase-I	GLS	0.9173903	lutaminase-R-(	R	122	Glutaminase-I	Normalized Li	0.96307177	1.04872102	1.04860547	0.90764963	0.97035782	0.99277767	1.03281959	0.95094971	0.96300489	0.90424998	0.99045877	1.01426716	1.00688588	1.00743007	1.04247379
GPBB-R-V_GB	PYGB	0.9487951	GPBB-R-V	R	123	GPBB-R-V_GB	Normalized Li	1.01393281	1.07874836	1.01479632	0.99142032	0.98939159	0.94249083	0.97038053	0.95085218	0.98581211	0.99189739	0.98972142	1.00139179	0.99397428	1.01976244	0.97288842
GSK-3a-b-M-\	GSK3A, GSK3B	0.9102799	GSK-3a-b-M-V	M	124	GSK-3a-b-M-\	Normalized Li	0.99737018	1.02159237	1.00680467	1.04237795	0.98045444	0.96474707	0.99063287	0.95172457	0.97552775	1.0219642	1.04671749	1.0544207	1.06935095	1.10486171	1.06792772
GSK-3a-b_pS2	GSK3A, GSK3B	0.950229	-3a-b_pS21_S9	R	125	GSK-3a-b_pS2	Normalized Li	1.1464146	1.32434248	1.1563419	1.24004159	1.09184313	1.09097529	1.14522656	1.02867785	0.97791968	0.94463314	0.93668134	0.91058724	0.94856483	0.8642599	0.9049083
Gys-R-V_GBL1	GYS1	0.9454421	Gys-R-V	R	126	Gys-R-V_GBL1	Normalized Li	1.03308969	0.87859998	0.94632301	1.00671855	0.91677787	0.87063303	0.94593379	0.94178229	1.01629225	1.17069961	1.10772905	1.02772625	1.06290323	1.15787101	1.03734417
Gys_pS641-R-	GYS1	0.9387068	Gys_pS641-R-\	R	127	Gys_pS641-R-	Normalized Li	1.05212116	1.11534042	0.99454465	0.86927567	0.84615551	0.84259018	0.97680938	1.06697526	1.04483935	1.0139751	0.95804337	0.95750388	1.02848864	1.00849916	1.02758369
H2AX_pS140-I	H2AX	0.8489842	2AX_pS140-M-	M	128	H2AX_pS140-I	Normalized Li	1.01435666	1.05137861	1.04365479	0.92337933	1.0080104	1.0015865	1.0128981	0.95957762	0.99407799	0.9664923	0.98671235	1.00115251	0.97068188	0.95918363	0.98472237
HER2-M-V_GE	ERBB2	0.8351647	HER2-M-V	M	129	HER2-M-V_GE	Normalized Li	1.01946206	0.96919712	1.00168629	1.05729347	1.01456956	1.09834204	0.99342052	1.01838668	1	1.00985304	0.96769156	0.96908083	0.98706694	0.95040212	0.92143215
HER2_pY1248	ERBB2	0.934989	:R2_pY1248-R-	R	130	HER2_pY1248	Normalized Li	0.917021	1.03160447	0.982127	0.94036889	1.07057827	1.03093597	0.94170718	1.00332911	0.99461324	0.91543579	0.96348918	0.99546331	0.89415048	1.02243062	0.98801558
HER3-R-V_GBI	ERBB3	0.9332283	HER3-R-V	R	131	HER3-R-V_GBI	Normalized Li	0.9510655	1.13102882	1.00815753	0.93487903	1.0104831	0.93618259	0.92278559	1.00866515	0.96291278	0.84984827	0.97125433	0.96144723	0.93549053	0.98686861	0.96051016
HER3_pY1289	ERBB3	0.9526471	:R3_pY1289-R-	R	132	HER3_pY1289	Normalized Li	0.97580227	1.08403517	0.93931392	0.9847681	1.02391209	0.92396042	0.93955713	1.02648372	0.98845062	1.00001467	0.98544135	0.95737972	0.95794644	0.98351965	0.96675693
Heregulin-R-V	NRG1	0.8988595	Heregulin-R-V	R	133	Heregulin-R-V	Normalized Li	0.96094589	1.03226595	1.00021357	0.94328796	0.97482636	1.04082555	1.01393196	0.99927509	1.00108749	0.9581583	1.04588504	1.06256646	1.00577959	1.04339659	1.07800373
HERV-R-N_GB	ERVK-18	0.8329626	HERV-R-N	R	134	HERV-R-N_GB	Normalized Li	1.03550683	1.07538187	0.99256676	1.08744121	0.98922809	0.94670402	1.08834941	0.9255908	0.9174862	1.05139646	1.04653704	1.03474311	1.07036009	0.98186394	1.00479638
HES1-R-V_GBI	HES1	0.9185211	HES1-R-V	R	135	HES1-R-V_GBI	Normalized Li	1.06886544	1.08228923	1.0833857	1.04687105	1.18941572	1.22219353	1.18723894	1.12420852	1.15291903	0.90722949	0.96399781	0.94357193	0.84003041	0.90413182	0.92093929
Hexokinase-II-	HK2	0.9243771	exokinase-II-R-	R	136	Hexokinase-II-	Normalized Li	0.9409786	1.00484232	1.0493888	0.98696072	1.02772244	1.04972467	1.03441905	1.05661298	1.07405929	0.84172985	0.95118569	0.96513781	0.84389746	0.95041608	0.97759859
HHLA3-R-N_G	HHLA3	0.8641111	HHLA3-R-N	R	137	HHLA3-R-N_G	Normalized Li	1.05377142	1.07656677	0.99695579	1.0904888	0.98602364	0.95242966	1.05770397	0.97283935	0.97485862	1.05828122	0.96763373	1.0140011	1.07474832	1.00457527	0.95862522
HIAP-R-C_GBL	BIRC2	0.9374353	HIAP-R-C	R	138	HIAP-R-C_GBL	Normalized Li	1.0518661	0.94597245	0.96554126	1.07517552	0.98985177	0.93155202	1.00966857	0.98753586	1.01718081	1.08915964	1.0046696	1.00686711	1.08628679	1.06130499	1.02042831
Hif-1-alpha-M	HIF1A	0.8108703	lif-1-alpha-M-(	M	139	Hif-1-alpha-M	Normalized Li	1.07187363	1.05320996	1.07924868	1.01466156	1.63022619	1.6174275	1.01666805	0.88478807	1.18705688	0.89568889	0.91240584	0.94930441	0.91669556	0.94013579	0.92141912
Histone-H3-R-	H3F3A, H3F3B	0.8624638	listone-H3-R-\	R	140	Histone-H3-R-	Normalized Li	1.06397492	1.14090108	1.05356733	1.23541433	0.98450132	0.96710173	1.21346442	1.01275491	0.98814316	0.96186435	0.91804644	0.9384341	1.08313731	0.94004823	0.90017518
Histone-H3-TI	HIST3H3	0.8804673	one-H3-TM-K4-	R	141	Histone-H3-TI	Normalized Li	0.81693046	1.52766844	0.77504057	0.76281034	1.1278771	0.77407777	0.75596958	0.85576257	0.80554882	1.20174864	1.24608804	1.19606022	0.98668274	1.15750964	1.27031003
HSP27-M-C_G	HSP27	0.8928767	HSP27-M-C	M	142	HSP27-M-C_G	Normalized Li	1.01062959	0.99407084	1.04683362	1.02851906	1.02214841	1.10958622	1.01337697	1.02758367	1.04605693	0.9534155	0.95441859	0.98595139	0.96309401	1.03951301	1.01707473
HSP27_pS15-F	HSPB1	0.9228663	SP27_pS15-R-I	R	143	HSP27_pS15-F	Normalized Li	1.08771756	1.06645948	1.00164932	1.1644328	1.00822897	0.94448578	1.05461678	0.96245948	0.95129941	1.08928626	0.95572457	0.94688286	1.06884451	1.02156123	0.92573749
HSP27_pS82-F	HSBP1	0.8768947	SP27_pS82-R-'	R	144	HSP27_pS82-F	Normalized Li	1.26317002	1.17546372	1.22503722	1.69942869	1.59748082	1.78707128	1.11827105	1.10966345	1.14924588	0.90988408	0.88827897	0.849086	0.64354578	0.76871407	0.82984668
HSP70-R-C_GI	HSP70	0.8803105	HSP70-R-C	R	145	HSP70-R-C_GI	Normalized Li	1.06030683	1.01056263	1.0279757	1.06764338	0.96879197	1.09133262	1.0470324	1.00147004	1.05026282	1.03193573	1.00274033	0.9879847	0.99823434	0.97768872	0.99986462
IGF1R_pY113!	IGF1R	0.9271817	_pY1135_Y113	R	146	IGF1R_pY113!	Normalized Li	1.00460916	0.98376618	1.02260703	1.05288646	1.01119124	0.97890333	1.03041328	0.99728527	0.99871686	1.00225324	1.00383586	1.02442323	1.0211291	1.01119952	0.97807437
IGFBP2-R-V_G	IGFBP2	0.8188251	IGFBP2-R-V	R	147	IGFBP2-R-V_G	Normalized Li	0.76152208	0.84362147	0.8325276	0.77636821	0.77627096	0.86324844	0.75927509	0.84970122	0.84731821	1.1817438	1.17293637	1.143355	1.16603637	1.1734916	1.16786984
IGFBP5-G-C_G	IGFBP5	0.8958787	IGFBP5-G-C	G	148	IGFBP5-G-C_G	Normalized Li	1.01289925	0.91223572	0.99961957	0.91907677	0.9950666	1.04201237	1.03949877	0.96415428	0.99556826	0.96954212	0.99160743	0.99415234	1.00590144	1.02588692	1.02216441
IGFRb-R-C_GB	INSR	0.9336832	IGFRb-R-C	R	149	IGFRb-R-C_GB	Normalized Li	0.88306306	0.96894512	0.96097943	0.88608068	0.93300485	0.92948718	0.85863256	0.89448997	0.92781995	1.10198231	1.10991422	1.13652197	1.09640705	1.19522721	1.17949612
INPP4b-R-V_G	INPP4B	0.9295444	INPP4b-R-V	R	150	INPP4b-R-V_G	Normalized Li	0.95417531	0.92223846	0.89848405	0.95930881	1.06907589	1.09148593	0.97212511	1.01985347	1.05990357	0.99510754	1.05547307	1.05049622	1.0263003	1.07492346	1.11000721
IRF-1-R-C_GBI	IRF1	0.8882111	IRF-1-R-C	R	151	IRF-1-R-C_GBI	Normalized Li	1.03679252	1.00509044	0.99080914	1.02035739	1.01308104	0.94596696	0.97231754	1.01637918	1.00329601	1.01269035	0.97659542	0.98841424	1.0061962	0.98996694	0.97047291
IRS1-R-V_GBL	IRS1	0.9271381	IRS1-R-V	R	152	IRS1-R-V_GBL	Normalized Li	0.88503148	1.01105329	0.95685553	0.90401921	1.04434917	1.00424387	0.90401901	1.03040135	0.98564196	0.91919686	1.00434515	1.01824865	0.92938716	1.06074519	1.0464941
JAB1-M-C_GB	COPS5	0.871194	JAB1-M-C	M	153	JAB1-M-C_GB	Normalized Li	1.02469142	0.9781581	1.03620259	1.01629205	0.97995419	0.9982321	1.03814654	1.01229355	1.0280815	0.99299751	1.00028775	1.01354302	0.98615165	0.99103155	0.96445446
Jagged1-R-V_(	JAG1	0.8710039	Jagged1-R-V	R	154	Jagged1-R-V_(	Normalized Li	0.94814764	0.97436634	0.99013529	0.87542733	0.98262728	1.01851536	1.02267158	1.01751788	1.02610518	0.90134294	0.98958374	0.98608181	0.92650319	0.95858319	0.9885334
Jak2-R-V_GBL	JAK2	0.9463828	Jak2-R-V	R	155	Jak2-R-V_GBL	Normalized Li	1.05560861	0.98824473	0.99985004	1.05503345	1.00448166	0.94317419	1.03133918	0.97416835	0.99533932	1.06286921	0.99086241	1.01965194	1.04100918	1.01371159	0.97717448

JNK_pT183_Y	MAPK8	0.9236522	_pT183_Y185-	R	156	JNK_pT183_Y	Normalized Li	0.9791302	1.01202383	0.99221152	1.08061113	1.01615749	1.04134928	1.02564682	1.01892305	0.99451354	0.99867641	1.01716431	1.01326347	1.00334159	0.99966641	1.0161676
JNK2-R-C_GBL	MAPK9	0.8910958	JNK2-R-C	R	157	JNK2-R-C_GBL	Normalized Li	0.97636166	1.35545088	0.97374464	0.98883815	1.06379989	1.03428361	0.94852463	1.06575386	1.03852316	0.94858609	1.03995323	1.01577045	0.97569086	0.95496578	1.06527866
LC3A-B-R-C_G	LC3AB	0.8582545	LC3A-B-R-C	R	158	LC3A-B-R-C_G	Normalized Li	0.94946157	0.94730961	1.00657967	0.9486818	0.97075041	1.00759029	1.03647159	1.00249634	1.02775058	1.03570933	1.00929854	1.01105549	1.017482	0.9787454	0.99393373
Lck-R-V_GBL1	LCK	0.8670948	Lck-R-V	R	159	Lck-R-V_GBL1	Normalized Li	1.03436267	1.14202259	1.04882215	0.92356384	1.00045769	1.0080395	0.97888871	1.00694987	1.00605403	0.96569372	1.05438416	1.02683814	1.02614446	0.96217248	0.99686182
LDHA-R-C_GB	LDHA	0.9109812	LDHA-R-C	R	160	LDHA-R-C_GB	Normalized Li	1.06734061	2.201377	1.12164803	1.02903401	1.09632724	1.11043531	1.124632	1.12273654	1.14877335	0.84865869	0.91927772	0.92722365	0.90646827	0.93122447	0.95499211
LINE-1-R-N_GI	L1RE1	0.9019935	LINE-1-R-N	R	161	LINE-1-R-N_GI	Normalized Li	1.02733451	0.99027687	1.07539627	0.99816684	0.99847494	1.03833443	1.02944146	0.96905334	1.00152169	0.99803112	1.01532882	1.0334253	0.99391215	1.01161602	0.99135158
MAPK_pT202	MAPK1, MAPK:	0.8617722	K_pT202_Y204	R	162	MAPK_pT202_	Normalized Li	1.08558347	0.88723585	0.88146397	2.20795365	2.16726981	2.26144404	0.89524033	0.92832404	0.9271617	1.50331461	1.64363794	1.63113771	0.94763137	0.92969995	0.94486618
Mcl-1-R-V_GB	MCL1	0.9449005	Mcl-1-R-V	R	163	Mcl-1-R-V_GB	Normalized Li	0.91115725	0.95150791	0.98273359	0.9121951	0.93465737	0.92515444	1.02938347	0.97686801	1.00048191	1.03954499	1.00036934	1.03260615	1.18108391	1.0395811	1.01911526
MCT4-R-V_GB	SLC16A4	0.9268923	MCT4-R-V	R	164	MCT4-R-V_GB	Normalized Li	1.23457725	1.230013	1.1686203	1.14825905	1.09450822	1.06875365	1.20445469	1.09633186	1.09328387	0.87782465	0.91076469	0.965707	0.85868058	0.83869651	0.84336185
MDM2-M-N_(	MDM2	0.8931682	MDM2-M-N	M	165	MDM2-M-N_(	Normalized Li	1.03257238	1.05201712	0.92886672	1.07254145	0.97895287	1.05003446	0.95877666	1.01768689	1.0154261	1.05643891	1.04855077	0.95318496	1.09447724	0.96159787	1.02428995
MDM2_pS166	MDM2	0.9427168	DM2_pS166-R	R	166	MDM2_pS166	Normalized Li	1.04065476	1	0.98603352	1.21865725	1.13080254	1.07122803	0.98094068	0.90782606	0.95090568	1.15779163	1.0262102	1.03743391	1.03784735	0.93190196	0.88812151
MDM2_pS395	MDM2	0.8263341	DM2_pS395-R	R	167	MDM2_pS395	Normalized Li	1.07077461	1.18497406	1.0440312	1.03775605	1.04242893	0.99492241	1.0353421	1.06116521	1.03381398	0.93613208	0.91796645	0.92173202	0.95566492	0.91766461	0.9167115
MEK1-R-V_GB	MAP2K1	0.9205973	MEK1-R-V	R	168	MEK1-R-V_GB	Normalized Li	1.07624523	0.98503991	0.94778196	1.0192019	0.96072945	1.02159977	1.07355107	1.04449955	1.00714238	0.95389112	0.98144705	0.9869651	1.03549759	0.9800998	1.02871175
MEK1_pS217	IAP2K1 MAP2K	0.9038856	1_pS217_S221	R	169	MEK1_pS217_	Normalized Li	0.94854701	0.95673119	0.98770387	1.27520783	1.12773524	1.12714281	0.98497261	1.01304889	1.04282914	0.97127736	1.0721238	1.06204758	0.95374172	1.01531515	1.00093454
MEK2-R-V_GB	MAP2K2	0.9278997	MEK2-R-V	R	170	MEK2-R-V_GB	Normalized Li	0.95737919	1.01129849	0.99638092	0.97817209	0.98729112	1.00195723	1.03465783	0.99177352	0.99652023	0.98901808	1.00350693	0.98731049	0.99811146	0.99723877	0.99557112
Merlin-R-C_GI	NF2	0.9478806	Merlin-R-C	R	171	Merlin-R-C_GI	Normalized Li	0.98708918	1.12489564	0.94877216	1.01001236	0.98417938	0.93966302	0.99477584	1.03562076	1.02358044	1.00636594	0.9553249	0.94875765	1.0044677	0.96280415	0.98051763
MIF-R-C_GBL1	MIF	0.8271377	MIF-R-C	R	172	MIF-R-C_GBL1	Normalized Li	1.04246873	1.0453748	1.0182362	1.0801866	0.96380682	1.00170721	1.08705826	1.06176673	1.02070964	0.9990542	0.94336037	0.94145566	0.97310655	0.94877808	0.9639443
MIG6-M-V_GI	ERRFI1	0.8755345	MIG6-M-V	M	173	MIG6-M-V_GI	Normalized Li	0.98694884	0.97046949	1.03432557	0.89050687	0.99221563	1.04206343	0.97674529	1.0075569	1.05405493	0.95316004	0.98419587	1.03060054	0.92296655	1.02222127	1.00583026
Mitochondria	MTCO2	0.8944486	itochondria-M	M	174	Mitochondria	Normalized Li	0.99709899	0.97794148	1.03229081	1.01163225	1.0067525	1.06125984	1.03684024	0.99702945	0.99442772	1.02307418	1.00884809	1.02119412	1.08039374	1.08411261	1.07233604
MMP2-R-V_G	MMP2	0.9307455	MMP2-R-V	R	175	MMP2-R-V_GI	Normalized Li	1.04612253	0.99955959	0.98009999	1.02323581	0.98660568	0.99282908	1.02602046	1.00987301	0.99704309	1.00096194	0.98139039	0.9826553	1.02390002	0.98670999	1.00324364
MSH2-M-V_G	MSH2	0.8370518	MSH2-M-V	M	176	MSH2-M-V_G	Normalized Li	1.01230495	0.95442068	1.04039068	0.97565263	1.00022757	1.08913075	0.98529053	0.99923359	1.06369124	1.00146668	1.08426705	1.04874607	0.97823912	1.05914899	1.10244964
MSH6-R-C_GB	MSH6	0.9487521	MSH6-R-C	R	177	MSH6-R-C_GB	Normalized Li	0.92471244	1.07720212	0.91807503	0.96499484	0.96095663	0.95002371	0.93065744	0.97622233	0.97353631	1.03807546	1.10441003	1.09044085	1.00594812	1.13265805	1.1383223
mTOR-R-V_GI	MTOR	0.959323	mTOR-R-V	R	178	mTOR-R-V_GI	Normalized Li	0.94553495	0.97583083	0.91281984	0.98800181	0.97191783	0.95665677	0.92783934	1.00191943	1.00352162	1.00520708	0.99288092	0.96783795	0.95596092	1.08351882	1.13277722
mTOR_pS244	MTOR	0.9541078	TOR_pS2448-R	R	179	mTOR_pS244	Normalized Li	1.00073371	1.2416994	0.93170672	1.16873115	1.2220769	1.13895186	0.89789494	0.95163688	0.9178491	1.02299114	1.0656048	1.04194265	0.95365716	0.89136566	0.91072871
Myosin-11-R-	MYH11	0.8632441	Myosin-11-R-V	R	180	Myosin-11-R-	Normalized Li	0.7933634	0.9414309	0.64553956	0.8150596	0.9830284	1.18734147	0.8132532	1.00055225	1.25595732	0.82256623	1.16395251	1.18430478	0.87723002	1.13499732	1.22723898
Myosin-IIa_pS	MYH9	0.9344085	sin-IIa_pS1943	R	181	Myosin-IIa_pS	Normalized Li	0.55349716	0.89905852	0.9057198	0.50107956	0.99344653	0.91957791	0.59573141	0.99409821	1.07911727	0.78123512	0.97560512	1.03017452	0.8798486	1.00788674	1.00879674
N-Cadherin-R-	CDH2	0.8200906	N-Cadherin-R-V	R	182	N-Cadherin-R-	Normalized Li	0.97134079	0.95742806	0.90709079	0.90113878	0.92020815	1.02831424	0.99988998	1.03306112	1.03819792	0.99197447	1.02382606	1.09038882	0.95584805	0.97627552	1.04900976
N-Ras-M-V_GI	NRAS	0.8689763	N-Ras-M-V	M	183	N-Ras-M-V_GI	Normalized Li	0.96737792	0.98361439	1.05619406	0.93660106	1.05061771	1.11177888	1.04572854	1.01058653	1.00952701	0.8812111	0.95371892	1.00701059	0.88545457	1.01463689	1.0224366
Nanog-R-N_GI	NANOG	0.8006219	Nanog-R-N	R	184	Nanog-R-N_GI	Normalized Li	1.00027295	0.98557835	1.03179456	0.91941713	1.01790792	1.05939568	1.03163378	0.98643542	0.99941968	0.97595053	1.00111413	1.08600279	0.98626317	0.98360873	1.0149381
NAPSIN-A-R-C	NAPSA	0.9204167	NAPSIN-A-R-C	R	185	NAPSIN-A-R-C	Normalized Li	1.03902344	0.98711468	0.99750768	1.09211184	0.9857888	0.99385965	1.07161646	0.9780848	0.98090809	1.04014107	0.99631604	0.99275871	1.06676482	1.01461456	1.00001889
NDRG1_pT34(	NDRG1	0.905045	DRG1_pT346-R	R	186	NDRG1_pT34(	Normalized Li	1.09047818	1.04490308	1.39086431	1.62774756	1.55556635	1.66372082	1.07443091	1.06278811	1.22066893	0.7132832	0.66188943	0.72529852	0.74612606	0.67965612	0.70225601
NDUFB4-M-V_	NDUFB4	0.8773668	NDUFB4-M-V	M	187	NDUFB4-M-V_	Normalized Li	1.02825639	0.97207126	1.0495444	1.08520722	1.00053121	1.02059967	1.03350823	1.01764497	1.00619853	1.02452897	0.99452003	1.00358644	1.02002148	0.99697437	0.96147189
NF-kB-p65_pS	RELA	0.9325978	kB-p65_pS536-	R	188	NF-kB-p65_pS	Normalized Li	1.35905963	1.3285158	1.13300781	1.27426327	1.21807142	1.06136221	1.07896114	1.23303492	1.09716038	0.77690589	0.80972644	0.81426359	0.89127549	0.84664692	0.85655534
Notch1-R-V_G	NOTCH1	0.9463401	Notch1-R-V	R	189	Notch1-R-V_G	Normalized Li	0.94265203	1.00775863	0.94122595	0.85751793	1.04842394	0.96019048	0.87706122	1.05794564	0.98448743	0.91192561	1.0456285	1.02606606	0.99841189	1.08891497	1.06412821
Notch3-R-C_G	NOTCH3	0.8897416	Notch3-R-C	R	190	Notch3-R-C_G	Normalized Li	1.06692405	0.92299783	0.94053664	1.02863731	0.94579979	0.95309044	1.05381065	1.00542582	1.01141073	1.03903973	0.97131416	0.95371108	1.0259347	0.94480931	0.97577104
Oct-4-R-N_GB	POU5F1	0.9120765	Oct-4-R-N	R	191	Oct-4-R-N_GB	Normalized Li	1.0095027	1.041218	1.0306361	0.97043629	0.99546951	1.00268083	1.02174623	0.95385235	0.9750601	0.98061376	1.0355313	1.01980578	0.98853218	0.99542052	0.9968776
P-Cadherin-R-	CDH3	0.9087755	P-Cadherin-R-C	R	192	P-Cadherin-R-	Normalized Li	0.9848361	0.99288983	0.99701316	0.87587874	0.98486999	0.97633842	0.98043577	0.99667282	0.98858954	1.04202532	1.07596772	1.03785387	1.07970859	1.01944414	1.0428033
p16INK4a-R-V	CDKN2A	0.8459974	p16INK4a-R-V	R	193	p16INK4a-R-V	Normalized Li	1.01508664	0.92230876	1.04205806	1.03626528	1.05841404	1.05469788	1.16886103	1.17602987	1.22197001	0.85273192	0.87661974	0.88268006	0.91290281	0.93311531	0.91010007
p21-R-V_GBL1	CDKN1A	0.894747	p21-R-V	R	194	p21-R-V_GBL1	Normalized Li	1.03885697	1.09676215	1.0269015	0.99059662	1.06992056	1.09088658	1.02847168	1.10147652	1.14890513	0.98031344	0.96158154	0.98487201	0.80269918	0.93187853	0.94199635
p27-Kip-1-R-V	CDKN1B	0.8950657	p27-Kip-1-R-V	R	195	p27-Kip-1-R-V	Normalized Li	0.95803618	0.95151758	0.93329674	0.97327693	1.00114826	1.01327454	0.92527187	0.9959955	1.0113265	0.99976675	1.01800326	1.02662638	1.0470198	1.03521438	1.07308026
p27_pT157-R-	CDKN1B	0.9273574	p27_pT157-R-C	R	196	p27_pT157-R-	Normalized Li	0.97779409	0.99541377	1.01539296	0.96701267	0.99474234	1.02527736	1.0035468	0.99484748	1.00097124	0.94792118	1	1.00844981	1.0057812	1.01271266	1.04140197
p27_pT198-R-	CDKN1B	0.9245763	p27_pT198-R-V	R	197	p27_pT198-R-	Normalized Li	1.01562908	0.97787243	1.00376787	1.06889653	1.01516918	1.02969451	1.03404837	1.01168451	0.99809102	1.05853384	0.99908994	1.02462466	1.05445916	0.98680826	0.98062275
p38-R-V_GBL1	MAPK14	0.9500325	p38-R-V	R	198	p38-R-V_GBL1	Normalized Li	1.01494661	0.96870034	0.99160632	0.93414815	0.95587053	0.92608849	0.89794601	0.99154339	1.00084236	1.08121876	1.00694943	0.97267228	1.09266588	1.01967559	0.99482107
p38_pT180_Y	MAPK14	0.8932371	_pT180_Y182-	R	199	p38_pT180_Y	Normalized Li	1.06788719	0.99041889	1.02798108	1.11883761	1.07371704	1.11499889	1.01100926	1.02029129	1.00190143	0.86497605	0.99503734	0.98302831	0.87071894	0.96633352	0.98762079
p53-R-C_GBL1	TP53	0.8864584	p53-R-C	R	200	p53-R-C_GBL1	Normalized Li	0.89609125	1.15664649	1.01742658	0.87108717	0.9694183	1.03921388	0.9331072	1.02062312	1.05341217	0.91195653	0.9718176	0.96194192	1	1.00411197	1.04474356
p70-S6K_pT38	RPS6KB1	0.9301917	p70-S6K_pT389-R	R	201	p70-S6K_pT38	Normalized Li	0.88282343	0.88905126	0.90082639	1.20804684	1.09830188	1.12603271	0.88765737	0.84755068	0.85709246	1.14735905	1.01275078	0.98930568	0.94072492	0.88340094	0.89222036
p70-S6K1-R-V	RPS6KB1	0.9150121	p70-S6K1-R-V	R	202	p70-S6K1-R-V_	Normalized Li	0.94945762	0.99659791	0.94272563	0.96991748	0.98666927	0.95025118	0.94341325	1.14313329	1.1567765	0.95963471	1.01290622	0.97187205	0.99075453	1.13983906	1.13875016
p73-M-N_GBL	TP73	0.8062819	p73-M-N	M	203	p73-M-N_GBL	Normalized Li	0.97551361	1.43496565	1.02203937	1.04359504	0.98955621	0.98548414	1.0215842	0.98438272	0.98564526	0.98025272	0.99546121	0.98117834	1.00080641	0.99458571	0.95830573
p90RSK_pT57	RPS6K	0.917321	0RSK_pT573-R	R	204	p90RSK_pT57	Normalized Li	0.93563158	0.91268196	1.00684776	1.44456216	1.4461562	1.48742426	0.99311665	0.95543525	0.98838805	1.08872721	1.18362383	1.21428058	0.9128161	0.96277659	0.95506169
PAI-1-M-V_GI	SERPINE1	0.871038	PAI-1-M-V	M	205	PAI-1-M-V_GI	Normalized Li	0.98940007	0.94012676	1.02141619	1.01370032	1.00007494	1.35741139	1.0096574	1.00606508	1.04795074	0.96285961	0.97620595	0.99602524	0.97257782	1.27556006	1.28585356
PAR-R-C_GBL	PAR	0.9401991	PAR-R-C	R	206	PAR-R-C_GBL	Normalized Li	1.06403554	0.94021392	0.91793389	1.10724167	1.03208931	1.15803273	0.99967989	0.97552274	0.99518184	1.3539266	1.07485796	1.1306832	1.74649467	1.17229775	1.10615201
PARP1-R-V_G	PARP1	0.9014168	PARP1-R-V	R	207	PARP1-R-V_GI	Normalized Li	0.89144355	0.80763092	0.86985819	0.85465628	1.02013777	1.00931768	0.82398265	0.97845347	1.01746889	0.97242909	1.19887922	1.20859481	1.01891321	1.1770514	1.16119627
Paxillin-R-C_G	PXN	0.927474	Paxillin-R-C	R	208	Paxillin-R-C_G	Normalized Li	0.83891898	1.00611384	0.90320119	0.86394409	1.02631469	0.97297728	0.77184485	1.06327611	0.95163392	0.79415858	0.99200363	0.95925942	0.84826993	0.97713097	1.00883496
PCNA-M-C_GI	PCNA	0.832929	PCNA-M-C	M	209	PCNA-M-C_GI	Normalized Li	0.99791671	1.00048978	1.0105607	0.99259415	0.99609462	0.9902491	0.97374787	0.96466514	0.99612039	1.01405758	1.01292834	1.04015038	0.98484132	1.02566836	1.07908858
PD-L1-R-C_GB	CD274	0.8208714	PD-L1-R-C	R	210	PD-L1-R-C_GB	Normalized Li	0.98906325	0.94652323	1.00493034	0.94849088	1.03088823	1.02113933	1.09594344	1.02194246	1.04995352	0.82891072	0.96119086	0.97140682	0.86804067	0.97195089	0.94984901
Pdcd-1L1-G-C_	CD274	0.899863	Pdcd-1L1-G-C	G	211	Pdcd-1L1-G-C_	Normalized Li	1.04650732	0.96983406	1.04650054	1.02219914	0.98571121	1.01568109	1.0801207	1.0143141	1.01062426	1.04943519	0.96919714	1.01506126	0.99975762	0.91595878	0.94681626
Pdcd4-R-C_GI	PDCD4	0.9476379	Pdcd4-R-C	R	212	Pdcd4-R-C_GI	Normalized Li	1.11574445	0.83810052	0.88325533	1.80339259	1.67608875	1.52465291	0.59378136	0.59529528	0.61192238	1.18670702	1.00509753	1.03743065	0.68187202	0.61467291	0.60396132
PDGFR-b-R-V_	PDGFRB	0.823688	PDGFR-b-R-V	R	213	PDGFR-b-R-V_	Normalized Li	0.87879851	0.89586145	1.03670937	0.84553174	1.00792383	1.0588247	0.90984974	1.036847	1.05460895	0.86935808	0.94709577	1.00284144	0.828092	1.00120763	0.98547038
PDK1-R-V_GB	PDPK1	0.9230653	PDK1-R-V	R	214	PDK1-R-V_GB	Normalized Li	1.07432033	1.11146412	0.99600202	1.06861071	1.05609377	1.02941484	0.98489199	1.05474037	1.04970478	1.01999215	0.98288775	0.9362037	1.00757556	1.01055742	0.99572419
PDK1_pS241-I	PDPK1	0.9452849	DK1_pS241-R-	R	215	PDK1_pS241-I	Normalized Li	1.07648635	1.04140996	0.98177514	1.07219343	1.02295168	0.99785009	1.00546296	1.09045528	1.03881907	1.01736326	0.95712719	0.92975794	1.04203793	0.95408188	0.97073566
PEA-15-R-V_G	PEA15	0.9584024	PEA-15-R-V	R	216	PEA-15-R-V_G	Normalized Li	0.95145058	1.11522527	0.95191756	1.00226828	0.95785564	0.96427974	0.9294596	0.97020828	1.04078148	1.04833159	0.99469365	1.0257688	1.01611576	1.03136055	1.03195961
PEA-15_pS11	PEA15	0.8925067	A-15_pS116-R	R	217	PEA-15_pS11	Normalized Li	0.96288179	0.98455042	1.00761437	0.97189282	1.02610225	1.06077982	0.97775782	1.01235053	1.01620892	0.95072201	1.01804652	0.99266892	0.93006347	1.0180942	0.98371307
PI3K-p110-a-R	PIK3CA	0.9294384	PI3K-p110-a-R-I	R	218	PI3K-p110-a-R	Normalized Li	0.94634235	0.96146748	1.00083166	0.87502748	0.96804433	0.97178931	0.92389884	0.96884752	0.99436422	1.03129513	1.05334071	1.06270204	0.97753132	1.04851235	1.07597414
PI3K-p110-b-N	PIK3CB	0.8817389	I3K-p110-b-M-	M	219	PI3K-p110-b-N	Normalized Li	1.04872265	1.02311037	1.02757667	1.02400499	0.97861947	0.98818828	1.02613204	0.99082132	0.99644628	1.03570646	1.00658843	1.01412233	1.01425283	0.98916088	0.98968797
PI3K-p85-R-V_	PIK3R1	0.9425186	PI3K-p85-R-V	R	220	PI3K-p85-R-V_	Normalized Li	0.96210426	1.0278229	1.01606479	0.97451792	0.9930124	0.98124773	0.88917596	0.99762403	0.98214245	0.94238071	1.02587676	0.99955898	0.96760748	1.02388038	1.01904453
PKA-a-R-V_GI	PRKAR1A	0.9349144	PKA-a-R-V	R	221	PKA-a-R-V_GI	Normalized Li	0.99768482	0.93582356	0.92891256	0.9728114	0.95837618	0.9230859	0.98104846	0.94271767	0.9901553	1.1047955	1.07086595	1.06494689	1.12545388	1.05530357	1.0093533
PKC-a-M-V_GI	PRKCA	0.8676138	PKC-a-M-V	M	222	PKC-a-M-V_GI	Normalized Li	0.9781092	1.05710886	1.01998659	0.96072431	0.97707006	1.01476384	1.0197387	0.99942495	1.01328306	0.93825408	0.9764249	1	0.95418601	0.97573271	1.00505144
PKC-a_pS657-	PRKCA	0.8739173	KC-a_pS657-R-	R	223	PKC-a_pS657-	Normalized Li	0.97069714	1.00381589	1.03487086	1.02002314	1.05318074	1.29579693	0.94433803	1.09459275	1.06538777	0.94368584	0.7656564	1.3062852	0.96528326	1.30179517	1.34520915
PKC-b-II_pS66	PRKCD, PRKCE	0.9257158	C-b-II_pS660-R	R	224	PKC-b-II_pS66	Normalized Li	0.860209	0.95029279	1.01705421	1.36775646	1.29516293	1.42414572	0.75097538	0.86627606	0.90466178	1.11304207	1.18300434	1.1793737	0.82953471	0.9970096	1.02007435
PKC-delta_pS	PRKCD	0.9408669	:-delta_pS664-	R	225	PKC-delta_pS	Normalized Li	1.0162408	0.99050096	0.99374897	1.00512695	1.00423414	1.02705481	1.01435458	1.00412531	1.02556927	1.06156563	1.02010508	1.01574202	1.0039898	1.02949305	1.03358259
PKM1-R-N_GI	PKM	0.8972811	PKM1-R-N	R	226	PKM1-R-N_GI	Normalized Li	1.03719533	1.01127439	1.01903593	1.07160388	0.98578838	0.97512671	1.01917732	0.99065159	1.00116513	1.05204288	0.95915137	0.97526925	1.04657969	1.00178321	0.97643611
PKM2-R-C_GB	PKM2	0.9599441	PKM2-R-C	R	227	PKM2-R-C_GB	Normalized Li	0.9958158	1.12172635	0.96841634	1.04852147	0.99965422	0.96879198	1.02913083	1.0292959	1.03103257	1.00346492	0.92341856	0.92353853	1.01936077	1.015729	0.98227215
PLC-gamma2_	PLCG2	0.9229278	gamma2_pY75	R	228	PLC-gamma2_	Normalized Li	0.98325258	0.99743938	1.01962647	0.99184447	0.99179487	1.01102903	0.99782168	0.97881671	0.99914622	0.94866982	0.988105	0.99686904	0.98400976	0.98798165	0.97400466
PLK1-R-C_GBL	PLK1	0.9274833	PLK1-R-C	R	229	PLK1-R-C_GBL	Normalized Li	0.79862875	0.66392657	0.73442906	0.79946855	0.70799968	0.70216162	0.76183798	0.67583815	0.69414004	1.40957863	1.44110201	1.4180383	1.44626756	1.47662147	1.41730074
PMS2-R-V_GB	PMS2	0.956519	PMS2-R-V	R	230	PMS2-R-V_GB	Normalized Li	0.74216494	0.8544497	0.76902395	0.75110291	0.7598106	0.70128464	0.70215919	0.73654357	0.72316453	1.26257846	1.48411971	1.40478952	1.29413432	1.42330378	1.41881557
Porin-M-V_GI	VDAC1	0.8889724	Porin-M-V	M	231	Porin-M-V_GI	Normalized Li	1.10616744	1.04637104	1.037526	0.95801072	0.98707846	0.96376073	1.08354291	0.84951734	0.99870745	0.83278579	0.82363691	0.95702285	0.86399041	0.95465414	0.90936179
PR-R-V_GBL1	PGR	0.9261374	PR-R-V	R	232	PR-R-V_GBL1	Normalized Li	0.93052166	1.06574223	0.96447599	0.91443559	1.02893797	0.97154195	0.92396591	1.01254882	0.98586878	0.8854951	0.98949211	0.95873259	0.97887039	1.0461653	1.02957764
PRAS40-M-C_	AKT1S1	0.8395792	PRAS40-M-C	M	233	PRAS40-M-C_	Normalized Li	0.97200541	1.03326914	1.03164792	0.92913879	0.96376077	1.03071008	0.98302597	0.97032337	1.01178873	0.91501231	1.0015	1.03365511	0.90349362	0.98990784	1.01981422
PRAS40_pT24	AKT1S1	0.8819283	AS40_pT246-R	R	234	PRAS40_pT24	Normalized Li	1.07303733	0.97809413	0.96801702	1.20902114	1.09641207	1.02251739	1.07334078	0.97017933	0.96182792	1.02959138	0.93911898	0.94826835	1.0632029	0.95589917	0.9078896
PREX1-R-V_GI	PREX1	0.8948529	PREX1-R-V	R	235	PREX1-R-V_GI	Normalized Li	1.00652777	0.94922353	0.99385475	1.02880953	1.00110806	0.98465132	1.09298237	0.92857983	0.9823254	1.02546471	0.89936768	0.89608689	1.11811188	0.93628614	0.95150377
PTEN-R-V_GBI	PTEN	0.9416883	PTEN-R-V	R	236	PTEN-R-V_GBI	Normalized Li	0.9065117	1.0119111	0.94326756	0.87969225	0.94132893	0.92274493	0.83794739	0.98265084	0.95720424	1.10995045	1.03845798	1.00396634	1.00782821	1.04417362	1.14709157
Puma-R-C_GB	BBC3	0.9007408	Puma-R-C	R	237	Puma-R-C_GB	Normalized Li	0.98704274	0.999776	1.08319681	0.9917979	1.01929632	1.0150338	1.01493582	1.02178278	1.06175805	0.93201211	1.01449394	1.00044174	0.95560083	1.03823183	0.98192983
PYGM-M-C_G	PYGM	0.9485729	PYGM-M-C	M	238	PYGM-M-C_G	Normalized Li	0.98315688	1.12706686	0.97191348	1.03441854	1.32223552	0.95661938	0.96364157	0.99115664	0.988898	1.00222795	0.98794944	0.95695144	1.01393859	1.38212718	1.09207474
Rab11-R-E_GI	RAB11A,B	0.9384512	Rab11-R-E	R	239	Rab11-R-E_GI	Normalized Li	1.05904528	1.02608418	1.0045659	1.0366106	1.0087388	0.99020304	1.03322157	0.98914818	0.97996356	1.05716672	1.03267045	1.01639965	1.01831442	1.01789434	0.98691459
Rab25-R-V_GI	RAB25	0.9265424	Rab25-R-V	R	240	Rab25-R-V_GI	Normalized Li	0.91498379	1.08051777	0.91501626	0.8930406	1.10652592	0.96885854	0.88480227	0.99272606	0.9436667	0.99636901	1.04290811	1.04451613	0.92682564	0.95384304	1.07355255
Rad50-M-V_G	RAD50	0.9149197	Rad50-M-V	M	241	Rad50-M-V_G	Normalized Li	0.84330662	0.99167522	1.00841004	0.90188727	0.98594044	0.95730076	1.03833275	1.05825483	1.04009011	0.92350131	0.9604608	0.93783064	0.89445897	0.98349661	0.95686268
Rad51-R-V_GI	RAD51	0.8645531	Rad51-R-V	R	242	Rad51-R-V_GI	Normalized Li	0.9000758	0.99544712	1.01898996	0.94811286	1.04016689	1.03481698	0.96744352	0.98274342	1.01889872	0.87234783	1.02049863	1.02745884	1.00103401	1.00684249	1.03946667
Raptor-R-V_G	RPTOR	0.9494215	Raptor-R-V	R	243	Raptor-R-V_G	Normalized Li	0.95467625	1.14929933	0.99331535	0.92008113	1.00033238	0.96808175	0.89045726	0.99218544	0.94615581	0.97672423	1.05186442	1.04490919	0.98849268	1.01930401	1.05753339
Rb-M-Q_GBL1	RB1	0.9085836	Rb-M-Q	M	244	Rb-M-Q_GBL1	Normalized Li	1.01980894	1.02067012	1	0.9981729	0.96689192	0.97205027	1.06792085	0.99984066	0.99519035	0.97740004	0.9787203	0.99208319	0.98063565	0.97474725	1
Rb_pS807_S8	RB1	0.9506759	_pS807_S811-F	R	245	Rb_pS807_S8	Normalized Li	0.85423492	0.84729888	0.80476319	0.94033931	1.03236156	0.94481287	0.80427452	0.78993616	0.74889603	1.2828287	1.28901504	1.25836616	1.07080374	1.15986042	1.19421525
RBM15-R-V_G	RBM15	0.9143124	RBM15-R-V	R	246	RBM15-R-V_G	Normalized Li	0.99842001	1.00764007	0.99516643	0.99864327	0.98367163	1.10160402	1.00732622	1.01615651	0.99160923	0.87573176	0.9713267	1.01511128	0.9397177	0.97813699	1.04506189
Rheb-M-C_GB	RHEB	0.8656866	Rheb-M-C	M	247	Rheb-M-C_GB	Normalized Li	1.02647141	1.2209145	1.00478942	0.85244103	1.02214663	0.99659588	1.01040692	0.98402594	0.99043273	0.99139951	0.9985554	1.0145257	0.93068999	0.98507244	1.01839302
Rictor-R-C_GB	RICTOR	0.9512512	Rictor-R-C	R	248	Rictor-R-C_GB	Normalized Li	0.86827564	1.04439846	0.93281359	0.74737392	1.13788092	0.96275909	0.66207233	1.12514286	0.96031993	0.7518363	1.00678686	1.00646833	0.87602781	1.10244345	1.02919148
Rictor_pT113	RICTOR	0.9495518	ctor_pT1135-R	R	249	Rictor_pT113	Normalized Li	1.01348655	1.08322946	1.03780846	1.08112285	1.0570449	1.03850338	1.03258568	1.0056026	0.98559002	0.98495329	0.96780795	0.97239853	0.95837024	0.9574274	0.96946909
Rock-1-R-C_GI	ROCK1	0.9208183	Rock-1-R-C	R	250	Rock-1-R-C_GI	Normalized Li	1.01992687	0.99707274	1.0164113	1.04084266	0.97343502	0.96507346	0.9832691	0.95363962	0.99950991	0.99348049	0.98515047	0.99246364	1.02503568	1.01995629	0.9851338
RPA32-T-C_GI	RPA32	0.9004647	RPA32-T-C	T	251	RPA32-T-C_GI	Normalized Li	1.06067781	1.0022334	1.00160046	1.16045393	0.98140688	0.95826243	1.0540588	0.98174779	0.96835598	1.04185972	0.95697212	0.95191302	1.08827206	0.96910881	0.95113604
RPA32_pS4_S	RPA32	0.9429719	A32_pS4_S8-R	R	252	RPA32_pS4_S	Normalized Li	0.93747624	1.1032014	1.04168286	0.95190761	1.04197049	1.0045943	0.94414629	1.04400872	0.98193382	0.95181599	0.9670164	0.98282132	0.94142777	1.00898436	0.99401615
RSK-R-C_GBL1	1, RPS6KA2, RI	0.9463071	RSK-R-C	R	253	RSK-R-C_GBL1	Normalized Li	0.9869662	1.01676068	0.9845032	0.98735764	0.9820564	0.9674736	0.9332351	0.96500452	0.98761585	1	1.04145123	1.03173768	0.99379492	1.06063564	1.08416035
S6_pS235_S2	RPS6	0.949254	_pS235_S236-F	R	254	S6_pS235_S2	Normalized Li	0.50504681	0.51764697	0.53751614	1.4889082	1.67833688	1.58322259	0.52583538	0.36185885	0.37566042	2.13819112	1.56629195	1.52856009	0.92596438	0.53911353	0.53169229
S6_pS240_S2	RPS6	0.9475416	_pS240_S244-F	R	255	S6_pS240_S2	Normalized Li	0.49696411	0.66161282	0.6036586	1.43432449	1.44761854	1.42255584	0.52000482	0.30234183	0.32327316	2.36248763	1.44848567	1.46353417	0.94878484	0.6018681	0.62357269
SCD-M-V_GBL	SCD	0.8824772	SCD-M-V	M	256	SCD-M-V_GBL	Normalized Li	0.94925629	1.10950394	1.03943546	0.93574102	1.07927757	1.05379057	1.00302978	1.05001326	1.04674843	0.91857928	0.92124843	0.96432283	0.90623159	0.98363559	0.99696929
SDHA-R-V_GB	SDHA	0.8707488	SDHA-R-V	R	257	SDHA-R-V_GB	Normalized Li	1.01917261	1.05567561	0.99614861	0.98463814	0.98571738	0.97854564	1.01488376	0.94042956	0.97551069	0.9761502	0.99488737	0.99683233	1.02007054	0.99661903	0.98981654
SF2-M-V_GBL	SRSF1	0.9028219	SF2-M-V	M	258	SF2-M-V_GBL	Normalized Li	1.02382215	0.99804533	1.00521808	0.98944265	0.97633652	0.98923458	1.00590399	1.03324242	1.00815462	0.99947665	0.98464203	1.02704575	0.97161142	0.9789391	0.97290133
Shc_pY317-R-	SHC1	0.9048151	Shc_pY317-R-V	R	259	Shc_pY317-R-	Normalized Li	0.95200395	0.97717961	0.9941691	0.97815647	1.000136	1.02771523	0.96646906	0.99368838	0.99838696	0.99456825	1.02388666	1.02199954	0.9804801	1.01476327	1.0471827
SHP-2_pY542-	PTPN11	0.9132258	HP-2_pY542-R-	R	260	SHP-2_pY542-	Normalized Li	1.13855041	0.97723694	1.0030017	1.31463243	1.13477072	1.06752992	1.18649624	0.98966688	0.93911401	0.95876932	0.96181194	0.97807702	1.02119516	0.90480797	0.87301328
SLC1A5-R-C_G	SLC1A5	0.9253396	SLC1A5-R-C	R	261	SLC1A5-R-C_G	Normalized Li	0.94013914	1.22381409	0.94538971	0.90753692	1.06154974	0.934442	0.85440355	1.02136116	0.94229313	1.09492277	1.01456925	1.10137702	1.08463134	1.01028899	1.06205096
Smac-M-Q_GI	DIABLO	0.9300665	Smac-M-Q	M	262	Smac-M-Q_GI	Normalized Li	1.06605111	1.00563851	0.97742152	1.0683716	0.98785625	1.00858136	1.03992392	1.09099217	1.05199563	0.98908213	0.93571819	0.92844043	1.0543235	0.97263941	0.98519163
Smad1-R-V_G	SMAD1	0.947775	Smad1-R-V	R	263	Smad1-R-V_G	Normalized Li	0.97683504	0.97418005	0.99022747	1.01256823	0.98387873	0.98219138	0.96746091	0.95849323	0.95054411	1.03087805	1.02065678	0.99891523	1.08382711	1.09857273	1.05967643
Smad3-R-V_G	SMAD3	0.9411816	Smad3-R-V	R	264	Smad3-R-V_G	Normalized Li	1.01685364	1.03597056	1.00189792	1.0376768	0.98008226	0.98167002	1.01357968	0.99209339	0.98919093	1.030988	0.9862197	0.98966981	1.03898234	0.9973471	0.99244002
Smad4-M-V_(	SMAD4	0.8261259	Smad4-M-V	M	265	Smad4-M-V_(	Normalized Li	0.97774561	0.96241906	1.06215216	0.88610733	1.01778537	1.13672643	0.99815748	0.97801162	1.0747282	0.91876103	1.03299857	1.0017076	0.90009087	0.98399419	1.03305327
Snail-M-Q_GB	SNAI1	0.8668347	Snail-M-Q	M	266	Snail-M-Q_GB	Normalized Li	1.00917838	0.97585736	1.00278454	0.96041862	0.98671527	0.99692175	1.03506715	1.01691093	1.03838903	1.02529817	1.01918359	1.00965273	1.03266483	1.0245646	1.03649671
SOD2-R-V_GB	SOD2	0.8577857	SOD2-R-V	R	267	SOD2-R-V_GB	Normalized Li	0.81809138	0.88470049	0.90337055	1.12714364	0.98125439	0.95551579	1.04262972	1.05883434	1.01154539	0.77796894	0.85040619	0.91726692	1.06587792	0.96292413	0.9405936
Sox2-R-V_GBL	SOX2	0.8928132	Sox2-R-V	R	268	Sox2-R-V_GBL	Normalized Li	0.95192409	0.96352217	0.99826843	0.93179873	1.04322937	1.11955428	0.97495318	1.03162798	1.0730319	0.88727357	0.99493644	0.96401547	0.88527113	0.95882017	0.96978309
Src-M-V_GBL1	SRC	0.8856108	Src-M-V	M	269	Src-M-V_GBL1	Normalized Li	1.06469039	1.02127849	1.0050181	1.13752686	0.96295515	0.99238669	1.09808984	0.92050011	0.95803545	1.02187552	0.98725177	1.00395014	1.04151696	0.99770684	0.96256537
Src_pY416-R-	N, FYN, LCK, YE	0.9165491	Src_pY416-R-V	R	270	Src_pY416-R-	Normalized Li	0.74064468	0.96519398	1.06957797	0.7913469	1.09749607	1.23120288	0.83466848	0.978548	1.08456064	0.88399375	1.19227661	1.19922747	1.12269149	1.33194136	1.49210048
Src_pY527-R-	C, YES1, FYN, F	0.9537665	Src_pY527-R-V	R	271	Src_pY527-R-	Normalized Li	1.03223056	1.02118647	0.97500192	0.98733567	0.98916002	0.99999883	0.99719588	1.00105701	0.9860468	1.02169574	1.03604238	0.9945979	0.99244645	0.99222778	1.06707737
Stat3-R-C_GBI	STAT3	0.9440012	Stat3-R-C	R	272	Stat3-R-C_GBI	Normalized Li	1.02731474	1.02510069	0.96755052	1.07518871	0.98542958	0.92462378	1.0072962	1.01598506	1.01466472	1.06785816	0.98039172	0.99044151	1.13978827	0.9493057	0.94908467
Stat3_pY705-I	STAT3	0.8956014	tat3_pY705-R-	R	273	Stat3_pY705-I	Normalized Li	0.90735731	1.06635037	0.93701039	0.87281562	1.12477029	1.15138804	0.92145175	0.95204051	1.07676692	0.87041442	1.06937227	1.09114103	0.85876533	1.05523026	1.08589001
Stat5a-R-V_GI	STAT5A	0.9324945	Stat5a-R-V	R	274	Stat5a-R-V_GI	Normalized Li	1.03840305	1.21325473	1.07851983	0.96317043	1.16192511	1.14623524	0.92922372	1.17159268	1.09918852	0.90152306	1.00079018	0.97910366	0.89298256	0.88681584	1.00188235
Stathmin-1-R-	STMN1	0.861221	Stathmin-1-R-V	R	275	Stathmin-1-R-	Normalized Li	1.03796125	0.97871049	0.99267154	1.00150189	1.0103313	1.01026821	1.02262708	0.98008589	1.01814018	1.05051302	1.06290195	1.03126674	1.007532	1.03952651	1.03975691
Syk-M-V_GBL	SYK	0.9363853	Syk-M-V	M	276	Syk-M-V_GBL	Normalized Li	1.03723675	1.03220579	1.02686115	1.06668307	1.0078086	0.96951228	0.9942955	1.01442908	0.99889776	1.07137658	1.00063592	1.00034413	1.05944276	1.0097241	1.00116975
Tau-M-C_GBL	TAU	0.9082193	Tau-M-C	M	277	Tau-M-C_GBL	Normalized Li	1.05839166	1.01657359	1.01967261	1.07416389	1.02031344	0.98711362	1.0266385	1.01401713	0.99929964	1.01945758	1.001733	1.00232463	0.99619997	0.96889725	0.89965271
TAZ-R-V_GBL1	WWTR1	0.9013576	TAZ-R-V	R	278	TAZ-R-V_GBL1	Normalized Li	1.00015164	1.01657454	0.99812091	0.92205336	0.98378424	1.00717058	1.02602307	0.96702124	1.01007892	0.93430625	0.98740022	1.00439297	0.95703339	0.98830894	1.00025107
TFAM-R-V_GB	TFAM	0.9178457	TFAM-R-V	R	279	TFAM-R-V_GB	Normalized Li	1.00556068	0.97037229	0.98874956	0.87793715	0.99875087	0.96903475	1.00314936	1.03658743	1.02295528	0.95231318	0.97553575	0.98682267	0.81185407	0.97631374	0.94237272
TFRC-R-V_GBI	TFRC	0.9318986	TFRC-R-V	R	280	TFRC-R-V_GBI	Normalized Li	0.93751075	2.23826618	0.91521166	0.98280874	1.23943521	0.90434446	0.95091863	1.13122666	0.97319134	1.06374956	0.94382357	0.96662419	1.14370917	0.91524582	0.99801384
TIF1-beta-R-N	TRIM28	0.9405592	TIF1-beta-R-N	R	281	TIF1-beta-R-N	Normalized Li	0.83931587	0.90510071	0.88416033	0.89653854	0.98966072	0.90205938	0.80199566	0.93051656	0.90816433	1.09056786	1.22204683	1.2028479	1.26213277	1.29837323	1.20956081
TIF1-beta_pS8	TRIM28	0.865233	l-beta_pS824-I	R	282	TIF1-beta_pS8	Normalized Li	1.06326506	1.03656849	1.10526627	1.07223849	1.0445455	1.07650025	1.06828596	1.07730475	0.92605134	0.59322979	0.95034678	0.95300182	0.94926024	0.9395908	0.95543326
TIGAR-R-V_GI	C12ORF5	0.9128413	TIGAR-R-V	R	283	TIGAR-R-V_GI	Normalized Li	0.96558001	1.05740881	1.00607501	1.04564771	0.96378946	1.03625649	1.08544007	0.96393194	0.98915568	1.0432663	1.01246404	1.00969066	1.03855971	1.03899574	1.08379132
Transglutamir	TGM2	0.92235	ısglutaminase-	M	284	Transglutamir	Normalized Li	1.04040994	1.00295995	1.01421516	0.89517541	0.98044249	0.96489089	1.02857388	0.99272224	1.01020567	0.8764323	0.96864326	0.97828352	1.02436432	0.98595889	0.94898477
TSC1-R-C_GBL	TSC1	0.9515891	TSC1-R-C	R	285	TSC1-R-C_GBL	Normalized Li	0.97278869	0.93054963	0.96964529	1.04431492	1.01882199	0.97037183	0.96059854	1.02272504	1.03458872	0.96451993	0.96574045	0.95799498	1.01229444	1.04271374	1.03444913
TTF1-R-V_GBL	NKX2-1	0.8662597	TTF1-R-V	R	286	TTF1-R-V_GBL	Normalized Li	1.04919541	1.06933205	1.00105018	1.05885492	0.96169439	0.97515245	1.00624063	0.97215514	0.99414715	1.09671049	1.06347374	1.02213689	1.07621904	1.02737692	1.03612084
Tuberin-R-V_(	TSC2	0.9541622	Tuberin-R-V	R	287	Tuberin-R-V_(	Normalized Li	0.97524039	0.96490819	0.95166327	0.91589053	1.00292155	0.99389219	0.90489449	1.00767972	0.97197016	0.96182611	0.974679	0.97810433	0.95713361	0.98517584	1.0465306
Tuberin_pT14	TSC2	0.8637062	berin_pT1462-I	R	288	Tuberin_pT14	Normalized Li	0.97573287	1.09118854	1.0099061	0.9853185	1.00484394	1.00173872	0.98589309	0.97337993	0.98535002	0.97976626	1.03608313	1.04699805	0.98574628	1	1.0266109
TWIST-M-C_G	TWIST2	0.8029286	TWIST-M-C	M	289	TWIST-M-C_G	Normalized Li	1.01294368	0.99827517	1.03674969	1	0.98502767	1.04323297	1.01918164	1.01475611	1.05896225	0.96161327	0.99362766	1.00644737	0.94896889	1.0080435	0.99431417
Tyro3-R-V_GB	TYRO3	0.8942839	Tyro3-R-V	R	290	Tyro3-R-V_GB	Normalized Li	0.99253776	0.97757175	1.04743864	1.00859628	0.99046555	1.03023998	0.9724616	1.00937499	1.03814301	0.93952043	0.9970694	0.98942435	0.97033424	0.97067963	0.97361603
UBAC1-R-V_G	UBAC1	0.94289	UBAC1-R-V	R	291	UBAC1-R-V_G	Normalized Li	1.07565591	1.12671134	0.99413652	1.07508346	0.9679641	0.94168025	1.0347059	1.01485159	0.99478347	1.0996741	0.99678229	1.01661259	1.05394694	0.98894018	1.01996727
Ubq-Histone-I	H2BFM	0.8631441	-Histone-H2B-	M	292	Ubq-Histone-I	Normalized Li	1.01873323	0.9664606	1.0529949	1.03177267	0.96366414	1.0186885	1.06667126	0.98885335	1.01188665	1.01334973	1.0250721	1.02165635	1.00392527	0.99574007	0.97063618
UGT1A-M-V_(	UGT1A1	0.8727862	UGT1A-M-V	M	293	UGT1A-M-V_(	Normalized Li	1.02728563	1.04662454	1.01910829	0.9902913	0.98961293	0.99499508	1.01612601	0.97825661	0.98558778	1.00858381	0.98462567	0.99572309	1.02638306	0.99984372	0.99960318
VEGFR-2-R-V_	KDR	0.940908	VEGFR-2-R-V	R	294	VEGFR-2-R-V_	Normalized Li	0.92938298	0.96576007	0.98458314	0.96849572	0.9832208	0.93477439	0.94672155	1.02099876	1.00447599	0.93038285	0.96208331	0.9975396	0.97226779	1.02031323	0.95969246
VHL-M-E_GBL	VHL	0.8842429	VHL-M-E	M	295	VHL-M-E_GBL	Normalized Li	1.88803702	0.73507908	0.7470201	1.74253282	0.99480779	0.9031059	1.02244569	0.95995767	1.01289865	1.50692698	0.71360908	0.7401885	1.13648604	0.74730159	0.74017689
Vimentin-M-C	VIM	0.8244129	Vimentin-M-C	M	296	Vimentin-M-C	Normalized Li	1.01337838	0.82935326	1.01666822	1.03004461	1.03741264	1.00205429	0.94546371	1.11683605	1.11501084	0.97236802	1.0316949	1.05642836	1.05149324	0.99602297	0.9768889
XBP1-G-C_GBI	XBP1	0.9168504	XBP1-G-C	G	297	XBP1-G-C_GBI	Normalized Li	1.01658382	1.11234186	1.00284674	1.01060699	0.99065895	1.02066809	1.01407945	0.97753036	0.99236251	1.02180391	1.01928581	1.01067825	1.01159731	0.99243956	1.02520448
XIAP-R-C_GBL	XIAP	0.9053317	XIAP-R-C	R	298	XIAP-R-C_GBL	Normalized Li	0.82507708	0.89443861	0.8943217	0.81343262	0.84926411	0.93870824	0.81724158	0.88976124	1.02058343	1.09108465	1.13847286	1.1328213	1.04037365	1.12063224	1.15334544
XPA-M-V_GBL	XPA	0.9241323	XPA-M-V	M	299	XPA-M-V_GBL	Normalized Li	1.00102144	0.98790452	0.97534512	0.98137139	0.99279266	0.99112387	0.93899013	0.98775165	0.98470997	1.03130654	1.07302783	1.00721087	1.00208185	1.05603959	1.05698134
XPF-M-C_GBL	XPF	0.9431391	XPF-M-C	M	300	XPF-M-C_GBL	Normalized Li	1.04808192	1.01266876	0.96682751	1.02265338	1.0144633	0.98475538	1.00665441	0.99547324	0.98829829	1.07507992	1.00221181	0.95752553	1.07409898	1.04244427	1.04355731
XRCC1-R-C_GI	XRCC1	0.9312436	XRCC1-R-C	R	301	XRCC1-R-C_GI	Normalized Li	0.97516031	0.887853	0.98952149	0.99457788	0.98057186	1.03082193	1.01075654	0.98019812	0.99475083	0.93786858	0.99349252	1.02794706	1.02655385	1.04858163	1.00299074
YAP-R-E_GBL1	YAP1	0.915059	YAP-R-E	R	302	YAP-R-E_GBL1	Normalized Li	0.9532485	1.08581598	1.0280982	0.99077884	1.03210365	1.05080152	0.96680487	0.97939185	1.00002185	0.93659632	0.97093702	0.9270257	0.94743203	1.01613139	1.00663274
YAP_pS127-R-	YAP1	0.9467449	YAP_pS127-R-E	R	303	YAP_pS127-R-	Normalized Li	1.06900305	1.06197118	1.00740745	0.99360973	1.08730811	1.01879664	0.83031649	1.01922751	0.95997878	0.87252845	0.8902873	0.8567411	0.81910776	0.93133366	0.86598613
YB1-R-V_GBL1	YBX1	0.901612	YB1-R-V	R	304	YB1-R-V_GBL1	Normalized Li	1.01825021	0.98007431	0.94063916	1.04573191	0.9641446	0.97233096	1.08365233	0.99230346	0.97507488	1.03902943	1.01784474	1.01467345	1.10569211	1.05762002	1.02398363
YB1_pS102-R-	YBX1	0.9241755	YB1_pS102-R-V	R	305	YB1_pS102-R-	Normalized Li	0.91699211	1.03036486	0.99314771	1.12797359	1.15748873	1.16332063	0.93648975	0.86948411	0.89536948	1.01541148	1.0422984	1.05989604	0.97769309	0.93694288	0.93137948
						V306																
						CF		0.93539503	0.99765639	0.99908042	0.85816182	1.01932608	1.07415407	0.9643216	0.98119395	1.00575692	0.90889623	1.03754238	1.05063742	0.91998689	1.07558912	1.09477117
